# Phototherapy in cancer treatment: strategies and challenges

**DOI:** 10.1038/s41392-025-02140-y

**Published:** 2025-04-02

**Authors:** Yeyu Cai, Tian Chai, William Nguyen, Jiayi Liu, Enhua Xiao, Xin Ran, Yuping Ran, Dan Du, Wei Chen, Xiangyu Chen

**Affiliations:** 1 Department of Radiology, The Second Xiangya Hospital of Central South University, Changsha, Hunan Province China; 2 Department of Radiology, Functional and Molecular Imaging Key Lab of Shaanxi Province, Tangdu Hospital, Fourth Military Medical University (Air Force Medical University), Xi’an, Shanxi Province China; 3 School of Chips, XJTLU Entrepreneur College (Taicang), Xi’an Jiaotong-Liverpool University, Taicang, Suzhou China; 4 Department of Oncology, The Second Xiangya Hospital of Central South University, Changsha, Hunan Province China; 5 Department of Dermatovenereology, The West China Hospital, Sichuan University, Chengdu, Sichuan Province China; 6 Laboratory of Dermatology, Clinical Institute of Inflammation and Immunology, Frontiers Science Center for Disease-related Molecular Network, West China Hospital, Sichuan University, Chengdu, China

**Keywords:** Translational research, Cancer therapy

## Abstract

Phototherapy has emerged as a promising modality in cancer treatment, garnering considerable attention for its minimal side effects, exceptional spatial selectivity, and optimal preservation of normal tissue function. This innovative approach primarily encompasses three distinct paradigms: Photodynamic Therapy (PDT), Photothermal Therapy (PTT), and Photoimmunotherapy (PIT). Each of these modalities exerts its antitumor effects through unique mechanisms—specifically, the generation of reactive oxygen species (ROS), heat, and immune responses, respectively. However, significant challenges impede the advancement and clinical application of phototherapy. These include inadequate ROS production rates, subpar photothermal conversion efficiency, difficulties in tumor targeting, and unfavorable physicochemical properties inherent to traditional phototherapeutic agents (PTs). Additionally, the hypoxic microenvironment typical of tumors complicates therapeutic efficacy due to limited agent penetration in deep-seated lesions. To address these limitations, ongoing research is fervently exploring innovative solutions. The unique advantages offered by nano-PTs and nanocarrier systems aim to enhance traditional approaches’ effectiveness. Strategies such as generating oxygen in situ within tumors or inhibiting mitochondrial respiration while targeting the HIF-1α pathway may alleviate tumor hypoxia. Moreover, utilizing self-luminescent materials, near-infrared excitation sources, non-photoactivated sensitizers, and wireless light delivery systems can improve light penetration. Furthermore, integrating immunoadjuvants and modulating immunosuppressive cell populations while deploying immune checkpoint inhibitors holds promise for enhancing immunogenic cell death through PIT. This review seeks to elucidate the fundamental principles and biological implications of phototherapy while discussing dominant mechanisms and advanced strategies designed to overcome existing challenges—ultimately illuminating pathways for future research aimed at amplifying this intervention’s therapeutic efficacy.

## Introduction

Phototherapy, a therapeutic approach leveraging exogenous agents to enhance the efficacy of light irradiation, has emerged as a promising method for cancer treatment, boasting attributes such as minimal invasiveness, high effectiveness, selectivity, and low toxicity.^1–3^ Phototherapy primarily encompasses Photodynamic therapy (PDT), photothermal therapy (PTT), and photoimmunotherapy (PIT). The former two utilize light to generate reactive oxygen species (ROS) or induce localized temperature increases for antitumor effects. PIT integrates the advantages of localized phototherapy and immunotherapy, which are capable of selectively killing cancer cells while activating polyclonal tumor-specific immune responses.^4^ Currently, phototherapy has been applied in clinical treatments for various cancers, including but not limited to skin cancer, colon cancer, prostate cancer, and breast cancer.^5–15^

The general mechanism of PDT involves three primary elements, namely light with a specific wavelength, a photosensitizer (PS), and molecular oxygen.^16–19^ Upon irradiation with specific-wavelength light, PSs generate ROS, which may contribute to cell death, microvascular system destruction, and immune responses, via two (Type-I and Type-II) routes. In PTT, after excitation by light at specific wavelengths, the photothermal agent (PTA) undergoes oscillatory relaxation of electron-excited energy, releasing decay in the form of nonradiative transitions (i.e., thermal energy). Subsequently, this process leads to the heating of the surrounding environment. In PIT, phototherapeutic agents (PTs) are combined with various immunotherapeutic drugs to not only induce necrosis of tumor cells but also trigger immunogenic cell death (ICD), thereby promoting a durable antitumor host immune response and addressing issues of immune suppression.^20^ PIT has reached critical preclinical and clinical stages and has rapidly evolved in recent years.

Despite the rapid advancement of phototherapy in cancer research, the majority of these methods have not yet been translated into clinical practice. This limitation is primarily due to the inherent constraints associated with different phototherapeutic approaches. In PDT, several challenges hinder its widespread application, including the low water solubility and poor tumor-targeting efficacy of PSs, which limit their accumulation and penetration in tumor tissues. The production of ROS is often hindered by several factors, including tumor hypoxia, the aggregation-caused quenching (ACQ) effect of photosensitizers, and the insufficient penetration depth of light within tissues. The risk of skin and ocular damage post-PDT, due to exposure to indoor or sunlight, further complicates its use.^21–25^ In PTT, the limitations include insufficient light penetration depth, which restricts the therapeutic reach to tumors outside the irradiated area. Additionally, thermal radiation can cause collateral damage to surrounding normal tissues. To address these challenges, the research community has implemented several significant efforts: (1) The development of novel nanoparticle PSs or PTAs, harnessing the unique properties of different materials to achieve superior physicochemical properties and enhanced stability, leading to higher efficiency rates of ROS or heat generation. Advances in nanomaterial research within the healthcare sector, particularly in theranostics, have identified several inorganic nanomaterials capable of high ROS generation rates and exceptional photothermal conversion efficiencies. Notably, precious metal materials, due to their inherent surface plasmon resonance (SPR) effect, can facilitate energy transfer to generate ROS and heat. Additionally, these materials can act as nanodiagnostic sensors by undergoing changes in the refractive index on the sensor surface due to alterations in surface mass, thereby allowing for interaction with various targets.^26–29^ (2) Enhancement of intratumoral oxygen content through endogenous oxygen production or exogenous oxygen delivery to enhance the effectiveness of PDT against tumors. (3) Augmentation of light utilization within tumors through the use of self-luminescent materials or NIR-I, NIR-II excited PTAs materials. (4) Amplification of the immune response post-phototherapy by integrating immune adjuvants and other agents. (5) Integration of different therapeutic approaches to achieve a synergistic amplification of therapeutic effects. So far, considerable efforts have been devoted to overcoming these limitations, as discussed in various reviews. Some of these reviews predominantly concentrate on nanoparticle-based solutions,^30–33^ while others provide overviews of diverse technologies aimed at addressing light penetration limitations.^34^ Some reviews focus on strategies to tackle hypoxic limitations within tumor tissues.^35–37^ Notably, certain excellent reviews comprehensively summarize the clinical progress of PDT in cancer treatment. For instance, Li et al. conducted a detailed review of advancements in PDT and PTT for the clinical treatment of various cancers. They also briefly outlined how emerging preclinical biomedical engineering approaches are addressing these phototherapy limitations.^13^ To the best of our knowledge, prior reviews have not systematically summarized the principles, biological effects, inherent limitations, and the most recent strategies to mitigate these limitations for PDT, PTT, and PIT. Given the critical importance of understanding these mechanisms for developing effective treatments, this review comprehensively explores the biological underpinnings of the antitumor effects of these phototherapies. It also examines the potential mechanisms contributing to their respective challenges, categorizing these into: (1) PTs; (2) hypoxia; (3) light penetration; and (4) inadequate immune response activation. Importantly, this review focuses on elucidating the emerging research strategies to address these limitations, along with their underlying mechanisms.

## Principles, biological effects, and application of phototherapy

### Principles of phototherapy

#### PDT

**Fig. 1:**
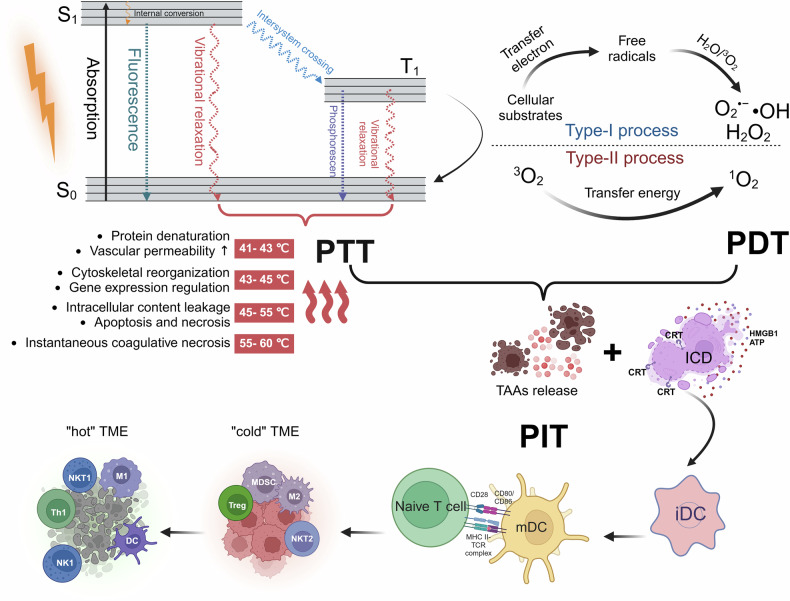
Schematic illustration of PDT, PTT, and PIT including Jablonski diagram, Type-I and Type-II mechanism of PDT, and ICD and reversal of TME in PIT. The interaction between incident photons and chromophores leads to an electron in the ground state (S0) being excited to a transient high-energy singlet state (S1). Subsequently, the electron in the excited S1 state undergoes intersystem crossing (ISC), forming a more stable and longer-lived triplet state (T1). This T1 state interacts with various substances through Type I and Type-II pathways, generating reactive oxygen species (ROS), which is the principle of PDT. Internal conversion (IC), the primary mechanism of PTT, involves the electron in the S1 state relaxing non-radiatively back to S0, releasing part of its energy as heat, causing a sharp increase in local tumor temperature. Both PDT and PTT can initiate an antitumor immune response via the mechanism of ICD. This process involves the release of a series of danger-associated molecular patterns (DAMPs) and cytokines, promoting the recruitment and maturation of APCs, cross-presentation, and phagocytosis. The tumor antigens are then presented to T cells, ultimately activating the antitumor immune response. This is the main mechanism of PIT. PTT photothermal therapy. PDT photodynamic therapy. PIT photoimmunotherapy. ICD immunogenic cell death. iDC immature dendritic cell. mDC mature dendritic cell. M1 type 1 macrophages. M2 type 2 macrophages. NKT2 naturalkiller T. MDSC myeloid-derived suppressor cells. NK1 natural killer 1. The figure was created with BioRender.com

Phototherapy operates through the illumination of PTs. During this process, incident photons collide with chromophores, resulting in scattering, transmission, or absorption. Only absorbed photons can effectively contribute to phototherapy. Absorption occurs when the energy of a photon matches the energy difference between two electronic states, causing an electron in the ground state (S_0_) to interact with the photon and transition to a transient higher-energy excited singlet state (S_1_). Following absorption, the photon’s energy is transferred to the electron, elevating it to S_1_ and ultimately returning to the ground state (S_0_) through a combination of radiative and nonradiative deactivation pathways. Radiative transitions refer to the process of energy dissipation through photon emission, including fluorescence and phosphorescence emissions. Nonradiative transitions consist of vibrational relaxation (VR), internal conversion (IC), and intersystem crossing (ISC), which constitute the primary mechanisms of PDT and PTT (Fig. 1). In PDT, electrons in the excited S_1_ undergo intersystem crossing (ISC) to form a more stable and longer-lived triplet state (T_1_) that can return to S_0_ through light energy release (fluorescence/phosphorescence) or vibrational relaxation. Importantly, T_1_ can interact with various substances through Type-I and Type-II routes to generate ROS. In the Type-I route, T_1_ engages in electron transfer with surrounding cellular substrates, forming free radicals capable of generating superoxide anions (O_2_^•−^), hydroxyl radicals (•OH), and hydrogen peroxide (H_2_O_2_).^38^ In the Type-II route, which is the primary mechanism contributing to the antitumor efficacy of PDT and is highly reliant on tissue O_2_, T_1_ transfers energy to nearby triplet oxygen (^3^O_2_) to generate cytotoxic singlet oxygen (^1^O_2_).^39^ Although Type-I and Type-II routes can occur simultaneously, the latter route is believed to be dominant for clinically proven PSs.^40^ In general, most PSs, such as organic dyes, generate ROS through a similar mechanism. However, as research progresses, an increasing number of inorganic nano-based materials have been found to produce ROS through different mechanisms. For instance, noble metal materials generate high-energy hot electrons through the localized surface plasmon resonance (LSPR) effect, which subsequently release energy through electron-phonon relaxation processes, thereby exhibiting high photothermal conversion efficiencies. Semiconductor materials generate ROS by forming electron-hole pairs that react with adjacent O_2_. The “Overcoming the phototherapeutic agents limitations” section provides a comprehensive review of the mechanisms of action of different inorganic nano-based photothermal agents.

#### PTT

In the above process, internal conversion (IC) serves as the principal mechanism in photothermal conversion. During this process, electrons in an excited state relax to the ground state through nonradiative means, causing collisions between the chromophore and its surrounding environment. Consequently, part of this energy is released as heat. The generated heat then diffuses into the surrounding medium, precipitating a sharp increase in the local temperature of the tumor. The goal is to elevate the temperature within the tumor region to inflict damage on tumor cells without causing immediate irreversible harm to the surrounding normal tissues^41,42^ (Fig. 1).

The effects of PTT on tumors are temperature-dependent. Light-induced heating can compromise cell membrane integrity, leading to chemical damage from Ca^2+^ influx.^30^ At low to moderate temperatures (approximately 41–43 °C), the heating, although not sufficient to directly kill tumor cells, can cause protein aggregation and denaturation. This can temporarily increase vascular permeability in the tumor area, improving the transport of drugs and oxygen. Furthermore, this temperature range may activate HSPs, which to some extent protect cells from thermal damage but also enhance the selectivity and efficacy of other treatments such as chemotherapy or radiotherapy by inducing heat shock proteins and altering tumor perfusion and metabolic status.^43^ At moderate temperatures (approximately 43–45 °C), PTT begins to have significant biochemical and morphological impacts on tumor cells, including partial protein denaturation, cytoskeletal reorganization, and gene expression regulation, generating ROS.^30^ As temperatures rise further to 45–55 °C, tumor cells undergo significant thermal stress with comprehensive protein denaturation accelerating, compromised cell membrane integrity, and intracellular content leakage, thereby promoting programmed cell death (such as heat-mediated apoptosis and necrosis). Additionally, such temperatures can also cause the destruction of structural components like collagen fibers in the tumor stroma, increasing drug diffusion and improving therapeutic outcomes.^44^ PTT at temperatures ranging from 55 to 60 °C can lead to the instantaneous coagulative necrosis of tumor cells, akin to the localized ‘cauterization’ of tumor tissues. However, such high temperatures may also cause severe damage to surrounding normal tissues, including vascular injury and inflammatory responses, and may lead to the overstimulation of heat shock proteins, suppressing the immune system’s anticancer activity. It is noteworthy that the location of PTAs—whether in the extracellular space, on the cell membrane, or inside the cell—significantly affects the efficacy of PTT. When PTAs are in the extracellular space, light energy is directly absorbed and converted into thermal energy, contributing maximally to the external thermal effect; however, the rapid dispersion of heat can prevent the accumulation necessary for effective cytotoxic action. When PTAs are on the cell membrane, their low thermal conductivity prevents easy heat dispersion, resulting in higher thermal gradients and more significant membrane damage, which can directly induce cell death. Thus, compared to the extracellular location, PTT targeted at the cell membrane is more direct and lethal. In contrast, PTAs within the cell, although potentially leading to heat accumulation, often have less effective photothermal effects due to uneven distribution within the cell and distance from the excitation source.^30^

#### PIT

Both PDT and PTT can initiate antitumor immune responses through the mechanism of ICD, which is the primary mechanism of PIT. ICD is defined as a form of regulated cell death (RCD) sufficient to activate an adaptive response in immunocompetent syngeneic hosts. This can be initiated by various stressors, including PDT and PTT. Healthy cells have limited capability to drive ICD, whereas tumor cells, due to the expression of a set of highly immunogenic antigenic epitopes, exhibit sufficient antigenicity to drive immune responses.^45^ Upon exposure to stressors, tumor cells release a large amount of damage-associated molecular patterns (DAMPs) and cytokines, which are associated with the initiation of adaptive immunity, including but not limited to calreticulin (CALR), high mobility group box 1 (HMGB1), extracellular adenosine triphosphate (ATP), and heat shock proteins (HSP70/90).^46^ The release of these DAMPs and cytokines can promote the recruitment and maturation of Antigen-Presenting Cells (APCs), their cross-presentation, the phagocytosis of dying cells, and the recruitment of T cells.^47^ Activated APCs migrate to lymphoid tissues, where they present tumor antigens to T cells capable of mediating tumor destruction, ultimately activating antitumor adaptive immune responses.^48^ Additionally, the tumor cell fragments released during phototherapy-induced cell death act as substrates for an in situ autovaccine, enhancing antitumor immunity,^49^ reversing the immunosuppressive ‘cold’ TME to an immune-activated ‘hot’ TME^50^ (Fig. 1).

### Antitumor biological effects of phototherapy

#### PDT and PTT-induced cell death

**Fig. 2:**
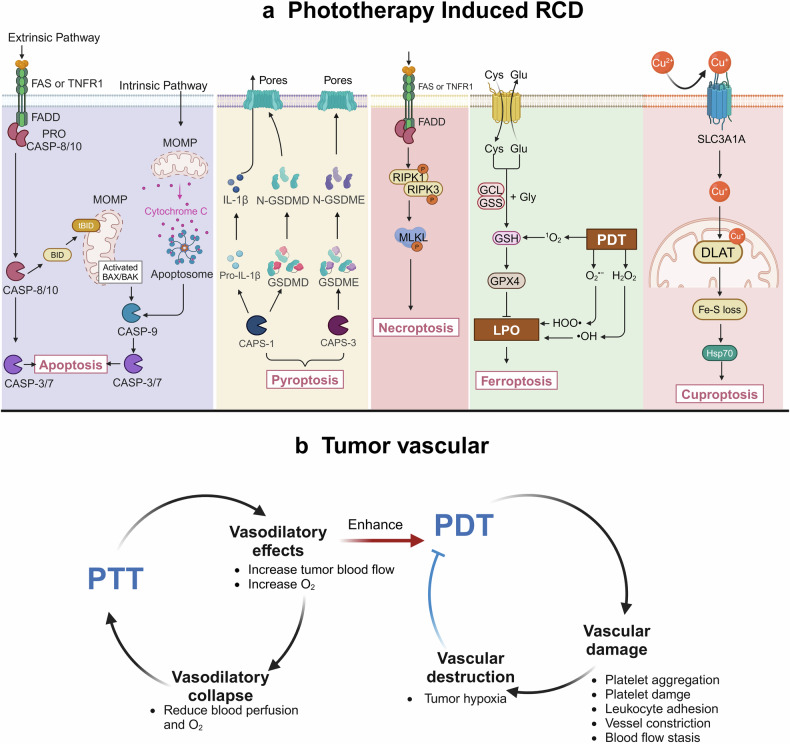
Scheme of the biological effects of phototherapy. **a** Major mechanisms of PDT and PTT-induced regulated cell death modalities, including apoptosis, pyroptosis, necroptosis, ferroptosis, and cuproptosis. **b** PDT and PTT affect the vascular system during the process. Low-dose or short-duration PTT can transiently increase blood flow and oxygenation levels within tumors, thereby enhancing the antitumor efficacy of PDT. However, high-intensity or prolonged PTT can cause thermal damage and collapse of the tumor vasculature, reducing blood perfusion and oxygen saturation within the tumor, which may diminish the therapeutic effects of PDT. PDT induces vasoconstriction, vascular damage, and inhibition of tumor angiogenesis through the release of various vasoactive compounds. This vascular damage can exacerbate tumor hypoxia, further reducing the efficacy of PDT. RCD regulated cell death. MOMP mitochondrial outer membrane permeabilization. LPO lipid peroxidation. The figure was created with BioRender.com

Recent studies have broadened the scope of PDT to encompass both accidental cell death (ACD) and RCD. ACD refers to an uncontrolled process where cells undergo death due to accidental, injurious stimuli that exceed the cell’s capacity for regulation, such as necrosis. In contrast, RCD is characterized by a structured cascade of signaling events that regulate orderly cell death, including apoptosis, pyroptosis, ferroptosis, necroptosis, and ICD among others. Within the context of PDT-induced cell death, these modes of cell death may occur independently or in combination.^51^ Furthermore, there is interconnectivity among different types of RCD. Although studies focusing on the cell death mechanisms in PTT are limited to date, the antitumor process of PTT similarly involves multiple forms of RCD. The exploration of these emerging cell death mechanisms offers new insights into the efficacy of PDT and PTT, as well as strategies for their enhancement. Moreover, recent research suggests that RCD may serve as an additional target for cancer therapy. This perspective underlines the importance of a comprehensive understanding of RCD mechanisms in enhancing the therapeutic efficacy of PDT and PTT against cancer. This review will primarily discuss five types of RCD induced by PDT and PTT: apoptosis, pyroptosis, necroptosis, ferroptosis, and cuproptosis (Fig. 2a). Given that ICD is a primary biological mechanism in PhotoImmunotherapy (PIT), it will be discussed in detail in “Phototherapy effects on the immune microenvironment” section.

### Apoptosis

Apoptosis is orchestrated through intricate pathways that are broadly categorized into two primary types: the intrinsic (mitochondrial) and extrinsic (death receptor) pathways. The intrinsic apoptosis can be triggered by various cellular alterations including, but not limited to, DNA damage, endoplasmic reticulum stress, ROS overload, and mitochondrial damage. A pivotal step in this pathway is the irreversible and extensive mitochondrial outer membrane permeabilization (MOMP), which is stringently regulated by the BCL-2 family of proteins, encompassing both pro-apoptotic and anti-apoptotic members.^52^ Following MOMP, apoptogenic factors normally residing in the mitochondrial intermembrane space, such as cytochrome c, somatic (CYCS), are released into the cytosol. CYCS then associates with apoptotic peptidase activating factor-1 (APAF1) and pro-caspase 9 (CASP-9) to form a supramolecular complex known as the apoptosome, which activates CASP-9.^53^ The activated CASP-9 cleaves and activates CASP-3 and CASP-7, enzymes considered to be executioner caspases responsible for the myriad of morphological and biochemical phenomena associated with apoptosis.^54^ Extrinsic apoptosis is initiated by disturbances in the extracellular microenvironment, driven by one of two types of plasma membrane receptors: death receptors (including Fas cell surface death receptor, FAS, and tumor necrosis factor receptor superfamily member 1A, TNFR1) and dependence receptors (including over twenty members such as netrin 1 (NTN1) receptors, DCC netrin 1 receptor (DCCN1)).^55,56^ Death receptors regulate the activation of CASP-8 (or to a lesser extent CASP-10) through the assembly of their homologous ligands into complexes, subsequently driving extrinsic apoptosis through two distinct pathways: (1) In “Type I cells”, the activated CASP-8 drives apoptosis through the cleavage and maturation of CASP-3 and CASP-7.^57^ (2) In “Type-II cells”, where the activation of CASP-3 and CASP-7 is inhibited, the cell undergoes apoptosis through the cleavage of BID by CASP-8, translocation to the mitochondrial outer membrane (OMM), and participation in BAX/BAK-dependent MOMP followed by CASP-9-driven apoptosis.^58^ Dependence receptors promote cell survival in the presence of their homologous ligands; however, caspase signaling cascades leading to apoptosis are activated once ligand levels fall below a certain threshold.^59^

Since the first report of PDT-induced apoptosis in tumor cells, apoptosis has emerged as the predominant type of RCD induced by PDT. This may be attributed to the common localization of PSs within mitochondria, which play a crucial role in apoptosis.^60^ Studies demonstrated that apoptosis induced by PDT is largely mediated through the activation of the intrinsic apoptotic pathway, such as the upregulation of CAPS-3 and CAPS-9,^61^ increased secretion of CYCS,^62^ and downregulation of Bcl-2 to stimulate the intrinsic apoptotic pathway. However, some research has shown that targeting PSs to mitochondria can induce extrinsic apoptosis through DNA fragmentation caused by PDT, activating CASP-8.^63^ Similarly, the conventional view holds that the primary mode of cell death caused by PTT is apoptosis. Several studies have confirmed that PTT can induce classical intrinsic pathway apoptosis by increasing the Bax/Bcl-2 ratio and activating CASP-3.^64–66^ However, to date, there are no studies on PTT-induced apoptosis through the extrinsic pathway.

### Pyroptosis

The term “pyroptosis” refers to a regulated cell death modality activated by inflammasomes and was coined to describe CAPS-1-dependent programmed cell death in macrophages associated with the release of IL-1β but distinct from apoptosis.^67^ Its characteristics include cellular swelling with the appearance of vacuoles, DNA fragmentation, chromatin condensation, and the formation of pores in the cell membrane, leading to the leakage of cellular contents.^68^ The main differences between apoptosis and pyroptosis: (1) During apoptosis, the cell membrane remains intact, whereas in pyroptosis, the cell membrane is damaged. (2) Different caspases regulate the two types of cell death. Pyroptosis involves two pathways: the canonical CAPS-1 inflammasome pathway and the noncanonical pathway triggered by CAPS-1 and CAPS-4/ CAPS-5. Gasdermin D (GSDMD) is believed to be the key in both pathways. Specifically, GSDMD can be cleaved by CAPS-1 or CAPS-4/ CAPS-5 to form GSDMD-C and GSDMD-N, with GSDMD-N subsequently binding to the inner plasma membrane and specifically interacting with phosphatidylinositol, generating a pore that rapidly permeabilizes the plasma membrane, thus inducing its lysis.^69^ Additionally, recent studies have reported that within the GSDM family, GSDMA and GSDME, similar in structure to GSDMD, also exhibit pore-forming and pyroptotic activities. Although the mechanism of GSDMA remains unclear, pyroptosis dependent on GSDME has been confirmed by research to be triggered by various inducers, including TNF and DNA damage. In this process, CAPS-3 is responsible for the proteolytic cleavage of GSDME.^70,71^

In recent years, there has been an increasing number of reports on PDT-inducing pyroptosis in tumor cells, broadly categorized into two main pathways: through GSDMD and GSDME. Li et al. revealed that PDT induces pyroptosis by downregulating pyruvate kinase M2 (PKM2) and subsequently activating CAPS-8 and CAPS-3, resulting in the production of GSDMD-N.^72^ Research indicates that pyroptosis induced by metal-based PS occurs through the GSDMD pathway, with no instances yet discovered of metal-based PS inducing pyroptosis through the GSDME pathway. For example, Wu et al. constructed nanoparticles TiO_2_@Ru@siRNA by coupling Ru with TiO_2_ and discovered that the PDT mediated by these nanoparticles upon irradiation causes cell pyroptosis through a CASP-1-dependent GSDMD pathway by damaging lysosomes.^73^ Additionally, Zhou et al. reported that a photosensitizer targeting mitochondrial translocase (IR700DX-6T), upon excitation, generates ROS that promotes downstream p38 phosphorylation and active CASP-3 cleavage of GSDME, thereby mediating cell pyroptosis.^74^ The PDT-induced pyroptosis faces certain limitations due to insufficient GSDM expression. Ding et al. utilized a photosensitizer (TBE) mediated PDT to cause mitochondrial damage in tumor cells, releasing DNA fragments, enhancing the cGAS-STING pathway, and promoting CASP-3 activation mediated cell pyroptosis; concurrently, using the STING agonist (Decitabine) to restore the expression of GSDME and STING, overcoming the limitations of PDT-mediated pyroptosis.^75^ Zhao et al. co-encapsulated indocyanine green (ICG) and decitabine, mediated a PTT effect after low-dose light activation, inducing CASP-3 activation. The released decitabine upregulates the expression of GSDME, synergistically inducing cancer cell pyroptosis.^76^

However, pyroptosis plays complex roles in tumor evolution; besides its antitumor effects, the inflammatory cytokines produced by pyroptosis may promote tumor progression and metastasis.^77^ Other studies suggest that pyroptosis can act as an ICD modality, increasing immune system response via the release of ICD hallmarks such as DAMPs and similar molecules.^78^ This character provides a new avenue for enhancing the efficacy of immune checkpoint blockade. Therefore, the balance between tumor promotion and inhibition by PDT and PTT-induced pyroptosis needs better understanding and further research.

### Necroptosis

Necroptosis is a programmed necrotic cell death modality that shares morphological characteristics with necrosis.^79^ It is characterized by cell swelling, plasma membrane permeabilization, and cellular content release but lacks several apoptosis hallmarks such as nuclear fragments, DNA cleavage, and caspase activations.^80^ Similar to apoptosis, necroptosis is also triggered by disturbances in the intracellular and extracellular microenvironment detected by specific death receptors (including, but not limited to, FAS, TNFR1) or pathogen recognition receptors (including TLR3, TLR4, and Z-DNA binding protein 1, ZBP1).^81,82^ Various signaling pathways, including receptor-interacting serine/threonine kinase 3 (RIPK3), activate mixed lineage kinase domain-like pseudokinase (MLKL), leading to the formation of specific signaling complexes. RIPK3 catalyzes the phosphorylation of MLKL, resulting in the formation of MLKL oligomers. Subsequently, these oligomers translocate to the plasma membrane, where they trigger plasma membrane permeabilization by binding to specific phosphoinositides.^83^ Notably, necroptosis also can act as an ICD modality, enhancing tumor-associated antigenicity and then provoking an antitumor immunogenic response.^84^

Some studies have reported that 5-aminolevulinic acid–based PDT produces singlet oxygen and can thus induce RIPK3-dependent necroptosis.^85^ However, the occurrence of necroptosis was shown to depend on tumor type, PS concentration, and irradiation dose. For instance, necroptosis and non-necroptotic necrosis were observed at low and high PS concentrations, respectively.^86^ Niu et al. developed a cell membrane-targeting photosensitizer with aggregation-induced emission (AIE) tendencies (TBMPEI) that can selectively accumulate on the cell membrane and induce necroptotic cell death upon illumination, accompanied by membrane rupture and DNA degradation.^87^ Han et al. developed a novel photosensitizer (Acy-5F) capable of rapidly enriching in the endoplasmic reticulum and initiating PDT under hypoxic conditions, causing DNA damage in cells. This leads to increased levels of phosphorylated RIPK1, RIPK3, and MLKL while targeting endoplasmic reticulum-induced autophagy limits the synthesis of CASP-8 and the degradation of phosphorylated proteins, ultimately inducing necroptotic cell death rather than apoptosis.^88^ Chen et al. designed a novel copper-based chalcogenide compound (CuS-NiS_2_) that, upon PTT activation by light irradiation, can also mediate necroptotic cell death in tumor cells through the MLKL/CAPG pathway. Research demonstrated that PTT mediated by CuS-NiS_2_ decreased the expression of Bcl-2, and increased the expression of Bax and phosphorylated MLKL, thereby inducing a dual mode of cell death characterized by both apoptosis and necroptosis.^89^ Also, Moros et al. discovered that gold nanoparticles, when used as photothermal agents, are capable of inducing necroptotic cell death through the RIPK1 pathway.^90^

### Ferroptosis

Ferroptosis is an RCD that depends on iron and lipotoxicity. It occurs through iron-catalyzed lipid peroxidation via Fenton reactions and lipoxygenases, without displaying hallmarks of apoptosis and necroptosis.^91^ Ferroptosis is independent of caspases which are characterized by morphological features of necrosis, including mitochondrial shrinkage, loss or disappearance of mitochondrial cristae, and cell rupture.^92^ Ferroptosis is primarily triggered via two pathways: the extrinsic (transporter-dependent) pathway and the intrinsic (enzyme-regulated) pathway. One key mechanism within the extrinsic pathway involves the membrane exchange transporter, such as the cystine/glutamate transporter (also known as system xc⁻), which facilitates the exchange of glutamate for cystine. Cystine is then reduced to cysteine, which, through the action of glutamate-cysteine ligase (GCL) and glutathione synthetase (GSS), is converted into glutathione (GSH). GSH acts as a reducing cofactor, and the GSH-glutathione peroxidase 4 (GPX4) antioxidation system plays a crucial role in protecting cells from ferroptosis. Therefore, inhibiting the xc⁻ system can prevent the synthesis of GSH, decrease the activity of GPX4, and thereby induce ferroptosis.^93^ The classical ferroptosis inducer (erastin) induces ferroptosis by inhibiting the system xc⁻, thereby reducing the intracellular levels of cysteine and GSH.^94^ The intrinsic pathway of ferroptosis involves the inhibition of intracellular antioxidant enzymes, most notably GPX4. GPX4 functions to reduce lipid hydroperoxides to their corresponding alcohols, thereby preventing oxidative damage to cellular membranes. Consequently, inhibiting GPX4 leads to the accumulation of lipid hydroperoxides on cell membranes.^95^

Research demonstrates that PDT induces ferroptosis through the generation of various ROS in distinct manners: (1) H_2_O_2_ generated by PDT can lead to the formation of •OH via the Fenton reaction, resulting in the oxidative modification of cell membrane phospholipids.^96^ (2) O_2_^•−^ generated by PDT can react to form HOO•, initiating the chain oxidation of polyunsaturated phospholipids.^97^ (3) O_2_^•−^ can further be converted into H_2_O_2_, which then continues to contribute to the induction of ferroptosis as described in the first mechanism. (4) ^1^O_2_ generated by PDT can react with unsaturated lipids to produce lipid hydroperoxides (LOOHs).^98^ (5) ^1^O_2_ can deplete GSH, thereby inducing ferroptosis.^99^ Recent nanoparticle-based PDT studies have emphasized PDT-induced ferroptosis, aiming to increase O_2_ levels to simultaneously enhance PDT efficacy and reinforce ferroptosis.^100^ Furthermore, combining ferroptosis with other cell death modalities induced by PDT may prove valuable in addressing tumor resistance and recurrence.^101^

The primary mechanism by which PTT induces ferroptosis may be attributed to PTT-induced mitochondrial dysfunction, which promotes the generation of mitochondrial reactive oxygen species (mitoROS), subsequently leading to ferroptosis. Current research on PTT-induced ferroptosis is limited, with most studies attempting to combine PTAs with other drugs to leverage the different mechanisms of action of these drugs to amplify ferroptosis induced by PTT. For instance, Chen et al. combined a PTA (TTHM NPs) with a non-steroidal anti-inflammatory drug (Sulfasalazine, SUZ), utilizing SUZ’s ability to inhibit the Glu/Cys reverse transport system xc⁻ to promote PTT-induced ferroptosis. Additionally, PTT was used to induce ICD. promoting DC maturation and CD^8+^ T cell aggregation. This approach, by inhibiting system xc⁻ and stimulating Acyl-CoA synthetase long-chain family member 4 (ACSL4), enhances ferroptosis.^102^ Ma et al. developed a multifunctional polydopamine (PDA)-coated manganese sulfide (MnS) nanocluster, harnessing PDA to endow MnS with an excellent photothermal conversion efficiency for effective PTT. Additionally, MnS releases Mn^2+^ in the acidic tumor microenvironment, exhibiting efficient peroxidase and glutathione oxidase-like activities, effectively inducing ferroptosis in tumor cells.^103^

### Cuproptosis

The latest research has uncovered a novel programmed cell death modality known as copper-dependent cell death, also referred to as cuproptosis, challenging our understanding of cell death mechanisms. Cuproptosis is a mitochondria-induced form of cell death distinct from apoptosis, ferroptosis, or necroptosis, but its sensitivity mechanisms remain unclear.^104^ Tsvetkov et al. explained the relationship between mitochondrial metabolism and cuproptosis. Specifically, in cells with active tricarboxylic acid (TCA) cycles, the levels of lipoylated TCA enzymes, particularly dihydrolipoamide S-acetyltransferase (DLAT), increased. Xu et al. proposed the use of glucose oxidase in combination with copper nanomaterials to enhance cuproptosis and PDT efficacy by increasing cellular copper levels and reducing glucose levels in cancer cells. They successfully developed the Gox@[Cu(Tz)] nanoplatforms, which, upon cellular uptake, catalyze the depletion of glucose and GSH in cancer cells. Subsequently, Cu(I) within the nanomaterial more effectively binds to lipoylated mitochondrial enzymes, leading to DLAT aggregation and inducing copper-dependent cell death. Additionally, glucose oxidation results in an elevation of H_2_O_2_, thereby activating Type-I PDT effects. This, in turn, generates a substantial amount of •OH through Fenton-like redox reactions.^105^

Wang et al. synthesized a photothermally active Cu-PDA nanomaterial by coordinating PDA with Cu2+, and then loading it with AuPt nanoparticles (NPs) to further endow it with catalytic activity. The resultant AuPt@Cu-PDA nanocomposite exhibited exceptional photothermal and catalytic efficiencies. Additionally, the accumulation of Cu^2+^ disrupted copper homeostasis, promoted the aggregation of DLAT, impaired the TCA cycle, and ultimately led to copper death.^106^ Wu et al. developed a unique Cu-C_3_ coordination structure, sputtered Cu single-atomzymes (CuSA), which not only possess excellent photothermal properties for precise PTT of tumors but also can release Cu ions in the presence of GSH to induce copper death.^107^

#### The impact on tumor vasculature

Different phototherapies variably affect and influence tumor vasculature during the process of combating tumors. The majority of studies indicate that the effects of PDT on tumor vasculature include: (1) inducing vascular damage. During the initial stages of PDT, platelet aggregation, platelet damage, and leukocyte adhesion to the vessel wall are observed, subsequently triggering a series of physiological responses, including vessel constriction, blood flow stasis, and thrombus formation.^108^ Additionally, PDT results in the release of various vasoactive compounds (e.g., eicosanoids, cytokines, clotting factors, and histamine), which primarily induce vessel constriction and increase vessel permeability. Specifically, the release of eicosanoids after irradiation increases the physiological ratio of proaggregatory-constricting compounds (such as thromboxane), leading to platelet aggregation and vessel constriction. Meanwhile, the cytokines released during PDT enhance vascular damage and potentiate leukocyte-endothelial binding.^48^ (2) Anti-angiogenesis. Lee et al. demonstrated that PSs, by accumulating in the Golgi apparatus and endoplasmic reticulum regions, reduce the expression of vascular endothelial growth factor (VEGF), thereby hindering tumor angiogenesis. Photoacoustic microscopy confirmed that within 3 h post-PDT treatment, both vascular strength and density significantly decreased.^109^ (3) Normalization of tumor vasculature. Research by Cavin et al. confirmed that low-dose PDT treatment (L-PDT) generates ROS, which through the Rho/ROCK kinase signaling pathway, leads to phosphorylation of myosin light chain and focal adhesion kinase (MLC-P, FAK-P). This results in the formation of actin stress fibers and pericyte contraction, enhancing pericyte-endothelial cell adhesion, thereby reducing intrinsic vascular permeability and normalizing tumor vasculature. The normalization of tumor vasculature can facilitate the delivery and distribution of large-molecule drugs within the tumor, enhancing therapeutic efficacy.^110^

Low-dose or short-duration PTT can transiently increase intratumoral blood flow and oxygenation levels due to the vasodilatory effects of thermotherapy, enhancing blood perfusion in the tumor region. However, high-intensity or prolonged PTT results in thermal damage and collapse of tumor vasculature, reducing blood perfusion and oxygen saturation within the tumor. This contributes to the effective eradication of tumor cells, particularly those in hypoxic zones that are more resistant^111^ (Fig. 2b).

#### Phototherapy effects on the immune microenvironment

According to the principles of PIT, phototherapy primarily induces ICD in tumor cells by affecting endoplasmic reticulum (ER) homeostasis, leading to ER stress.^112^ Specifically, following stimulation by phototherapy, cells experience ER stress and rapidly initiate the unfolded protein response (UPR) to maintain endoplasmic reticulum homeostasis. The UPR aims to restore the ER protein folding capacity by increasing ER volume, expression of ER-associated molecular chaperones, and attenuating protein translation. It is controlled by three ER stress sensors: inositol-requiring enzyme 1 alpha (IRE1α), protein kinase R-like ER kinase (PERK), and activating transcription factor 6 (ATF6).^113^ Mild ER stress tends to maintain endoplasmic reticulum homeostasis, promoting tumor survival. However, when the intensity of ER stress is excessive, the UPR initiates signaling pathways, releasing immunostimulatory factors and DAMPs, leading to ICD.^114^ PIT-induced ICD and the release of DAMPs involve multiple mechanisms, including PERK, Bax, Bak, and the secretory pathway. For instance, following PIT treatment, PERK is associated with an increase in intracellular Ca2+ and changes in the actin cytoskeleton (the specific mechanisms remain unclear); CALR is exposed on the cell surface by binding to the CD91 receptor; the secretion of ATP follows a pathway highly overlapping with that of CRT and is partially involved with CASP-8.^115^ As dying tumor cells release various DAMPs through processes such as the recruitment and activation of APCs in the tumor microenvironment, the maturation of DCs, and antigen presentation to cytotoxic T cells, an effective antitumor immune response is ultimately triggered.^47^ Moreover, phototherapy can generate a highly immunogenic TME, including increasing the expression of programmed death-ligand 1 (PD-L1) and tumor-infiltrating lymphocytes (TILs), reprogramming M2 tumor-associated macrophages (TAMs) into M1 TAMs, and thus reversing the immunosuppressive ‘cold’ TME into a ‘hot’ TME, making it more responsive to immunotherapy.^116^

However, both PDT and PTT also encompass mechanisms that could potentially lead to immunosuppression. Some clinical cases suggest that the immune activation induced by PDT occurs predominantly in the early stages. As the treatment progresses, PDT exerts an immunosuppressive effect on the TME, though the mechanism remains unclear.^117^ L et al. suggest that transforming growth factor-beta 1 (TGF-β1) plays a key role in mediating immunosuppression. Specifically, in the later stages of PDT, the production of ROS induces local inflammation, recruits immune cells, and upregulates the expression of the CD44 receptor, thereby recruiting MMP-9 and TGF-β1. Moreover, coupling PDT with a TGF-β inhibitor significantly improves tumor cure rates.^118^ Additionally, some research suggests that the immunosuppression triggered by PDT is associated with the dynamic impact on TAMs. Research indicates that during PDT treatment, the tumor lesion undergoes intense acute inflammation, marked by rapid and substantial infiltration of neutrophils, mast cells, and newly recruited monocytes. At this stage, the resident TAMs, predominantly of the tumor-promoting M2 macrophages, are replaced by newly differentiated antitumor M1 macrophages derived from infiltrating monocytes. This is why, in the early stages of PDT treatment, there is a predominant induction of an antitumor immune-activated tumor microenvironment. However, in the later stages of PDT treatment, to address the induced inflammation, regulatory mechanisms are activated, resulting in the construction of a significant number of anti-inflammatory, tumor-promoting M2 macrophages. These macrophages release mediators that inhibit inflammation and immune responses, promoting tumor angiogenesis and tumor recurrence.^117^

Compared to research on the immune response activated by PDT, there is relatively less study on the immunosuppression induced by PTT and its associated molecular mechanisms. PTT may inhibit the immune components and functionalities within the TME through several mechanisms: During PTT, hyperthermal effects cause constriction and rupture of tumor vasculature, potentially reducing the effective infiltration of immune cells into the tumor area. Hyperthermal damage to vascular endothelial cells impairs the adhesion and transvascular migration capabilities of leukocytes, limiting their access to the tumor core.^42^ Additionally, PTT-induced overexpression of HSPs includes some with immunosuppressive functions, such as HSP70, which protects tumor cells from heat-induced cell death and contributes to the establishment of immune evasion mechanisms.^119^ Moreover, excessively high temperatures may also impair the functionality of immune cells.

### Application of phototherapy

#### PDT

**Fig. 3:**
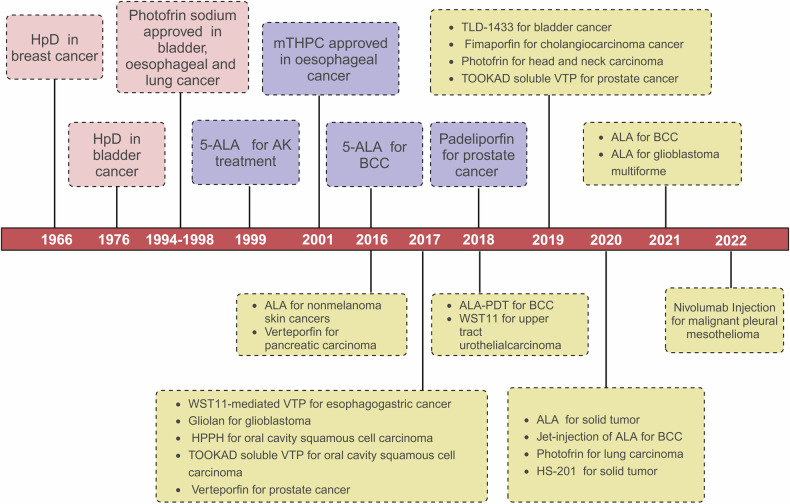
The timeline of photosensitizers used in PDT for cancer treatment. The timeline encompassing first-generation and second-generation PSs, as well as those currently under clinical trials. HpD hematoporphyrin derivative. mTHPC temoporfin. ALA 5-Aminolevulinic acid. AK actinic keratosis. BCC basal cell carcinoma. VTP vascular-targeted PDT. HPPH photochlor. The figure was created with BioRender.com

Since the first discovery of hematoporphyrin in human blood in 1841, it was purified and chemically modified for several decades and applied to clinical diagnosis and treatment. In 1966, hematoporphyrin derivative (HpD), the first-generation PSs, was reported to use in breast cancer treatment.^120^ After that, Kelly and Snell reported HpD in bladder cancer treatment in 1976.^121^ Until 1993, Photofrin (porfimer sodium), a purified component of HpD, was first approved for bladder cancer treatment in Canada.^13^ From then on, the curtain was raised for the application of PDT in cancer treatment. Subsequently, between 1994 and 1998, Photofrin was successively approved for clinical application in the treatment of esophageal and lung cancer in multiple countries.^122^ In 2003, HpD was approved for precancerous high-grade dysplasia in patients with Barrett's esophagus.^123^ Despite these approvals, the first-generation PSs didn’t widely used in the treatment of solid tumors because of important disadvantages. The second-generation PSs are then developed to overcome the disadvantages associated with the first-generation PSs. In 1999, the second-generation PSs, Porphyrin precursor of 5-Aminolevulinic acid (5-ALA) was approved for non-hyperkeratotic actinic keratosis (AK) in USA, Korea, Mexico, Brazil, Argentina, Chile, and Colombia.^124^ 5-ALA was also approved to the treatment of basal cell carcinoma (BCC) and daylight PDT by the EMA in 2016.^13^ In 2018, Padeliporfin was approved for prostate cancer treatment in Mexico and EMA.^125^ Although, lots of PSs have been approved by the FDA for PDT, many preclinical and clinical trial are still ongoing now. According to the ClinicalTrials.gov database, in 2016, a trial of ALA for nonmelanoma skin cancer and a phase 2 clinical trial of Verteporfin for pancreatic carcinoma were recruited. In 2017, WST11-mediated vascular-targeted PDT (VTP) for esophagogastric cancer (phase 1), Gliolan (5-ALA-protoporhyrin IX, 5-ALA-PpIX) for glioblastoma, HPPH (photochlor) for oral cavity squamous cell carcinoma (phase 2), TOOKAD® soluble VTP for oral cavity squamous cell carcinoma (phase 2) and Verteporfin for prostate cancer (phase 1) were recruited. ALA-PDT (Ameluz-PDT) for BCC (phase 3) and WST11 for upper tract urothelial carcinoma (phase 1) in 2018, TLD-1433 (Ru (II) polypyridyl complex) for bladder cancer (phase 2), Fimaporfin for cholangiocarcinoma cancer (phase 2), Photofrin for head and neck carcinoma (phase 2) and TOOKAD® soluble VTP for prostate cancer (phase 4) in 2019 were activated and recruited. In 2020, Jet-injection (AirGent2.0) of ALA (levulan® kerastick®) for BCC (phase 2), HS-201 for solid tumor (phase 1) were recruited. In 2021, ALA for basal cell carcinoma (phase 2), ALA for glioblastoma multiforme (adult) (phase 2) were recruited. And then, in 2022, Nivolumab injection for malignant pleural mesothelioma (phase 2) was recruited. The timeline of the clinical trials of PDT is summarized in Fig. 3.

#### PTT

The extensive research on nanomaterials capable of converting external photons into thermal energy for PTT applications is primarily categorized into organics, metals, carbon, and semiconductors. Organic photothermal agents mainly include organic dyes, polymeric nanoparticles, and porphysomes. Among all organic dyes, ICG is the only FDA-approved photothermal agent.^126^ Despite its drawbacks such as unstable optical properties, rapid circulation kinetics, and non-selectivity for tumors, ICG has still made considerable progress in research.^127–129^ Li et al. reported the clinical translation of ICG PTT for the local ablation treatment of refractory metastatic breast cancer patients.^130^ In metal photothermal agents, gold nanoparticles are widely utilized due to their surface plasmon resonance (LSPR) properties, providing strong absorption in the NIR window. The absorption peak of gold nanoparticles can be tuned by adjusting their size, shape, and shell thickness to achieve optimal photothermal conversion. Various forms of gold nanostructures, such as nanoparticles,^131^ nanorods,^132^ nanoshells,^133^ Au NCs,^134^ and hollow nanospheres,^135^ have been developed for PTT treatment, each with its own characteristics. In 2019, a phase I clinical trial demonstrated the feasibility of using sterile nanoshells with a silica core and gold shell for local PTT treatment of prostate cancer.^136^ Semiconductor photothermal agents are favored in PTT research due to their highly efficient photothermal conversion rate, high NIR absorption capacity, and resistance to reshaping or bleaching under NIR. Copper sulfide (CuS) is a crucial sulfur-based semiconductor material that exhibits broad absorption ranging from 700 to 1100 nm. Its interaction with NIR can generate heat for PTT treatment.^137^ However, CuS PTT efficiency is relatively low, and researchers have made various efforts, such as utilizing the LSPR effect of metal nanoparticles (Ag, Au, etc.) to enhance the optical absorption of incident photons near the nanoparticles, thereby enhancing the PTT conversion efficiency of CuS.^138,139^ Although PTT holds significant potential in cancer therapy, challenges such as the lack of thermal control, difficulty in activating deep-seated agents effectively with light, and potential biotoxicity of photothermal agents significantly hinder its clinical translation.

#### PIT

The concept of PIT, integrating immunotherapy with phototherapy, was initially proposed in 1983, representing a form of targeted photodynamic therapy. This therapy involves the use of antibodies conjugated with traditional photosensitizers, such as hematoporphyrins, to induce cytotoxicity in target cells via the generation of ROS. However, it failed to elicit an effective systemic antitumor immune response.^140^ Preclinical studies have confirmed the efficacy of PIT using a variety of photosensitizers, such as mTHPC, pheophorbide a (PPa), and chlorin e6 (Ce6), conjugated with multiple monoclonal antibodies (mAbs).^141,142^ In 2011, Kobayashi discovered that an epidermal growth factor receptor (EGFR) mAb conjugated with the phthalocyanine dye (IRDye 700DX) was found to immediately induce cell death in EGFR-expressing cells upon irradiation.^143^ In 2014, Spring and colleagues combined the photosensitizer benzoporphyrin derivative (BPD) with cetuximab (an FDA-approved anti-EGFR monoclonal antibody) for selective treatment of micrometastases in an advanced ovarian cancer model in vivo.^143^ Subsequently, a multitude of studies emerged, exploring the combination of various antibodies, such as the Human epidermal growth factor receptor 2 (HER2)-specific antibody trastuzumab, delta-like protein 3 (DLL3) monoclonal antibody rovalpituzumab, anti-podoplanin antibody NZ-1, and anti-PSMA monoclonal antibody, with IRDye 700DX for the treatment of different tumors.^144–147^ In 2015, a Phase I clinical trial (NCT02422979) was officially launched to evaluate the safety and antitumor activity of RM-1929, a conjugate of IR700 with an EGFR-targeting antibody, in patients with advanced head and neck cancer. This was followed by a Phase II clinical trial evaluation in 2016 and a Phase III clinical trial in 2019. Consequently, NIR-PIT received approval from Japanese regulatory authorities in 2020 for official clinical use.^148^

The earliest proposition of combining PTT with immunotherapy was introduced in 1997 with the advent of Laser Immunotherapy (LIT). This innovative approach combined PTT, using ICG, with the in situ application of an immunological adjuvant (N-dihydrogalactochitosan, GC) to induce an antitumor immune response in the host.^149^ Currently, there has emerged a therapeutic strategy for breast cancer that employs the selective thermal effect of ICG in combination with an immunological adjuvant (glycol chitosan) for immunostimulatory treatment.^130^ Several clinical trials have employed imiquimod, a Toll-like receptor agonist, as an immunological adjuvant for photothermal immunotherapy in melanoma.^150^

## Overcoming the phototherapeutic agents limitations

As primary components in phototherapy, phototherapeutic agents are crucial in the therapeutic framework. Phototherapy is categorized based on its mechanism into PDT using photosensitizers (PSs) and PTT utilizing photothermal agents (PTAs). Currently, clinically approved photosensitizers are predominantly used in PDT. As mentioned previously, first-generation PSs encountered significant challenges upon development and clinical application: (1) Limited penetration due to their excitation by short wavelengths (UV-Visible light), restricting effectiveness on deep-seated tumors. (2) Low molar extinction coefficient (~1.17 × 10^3^ mol/L cm), necessitating larger dosages. (3) Weak penetration and accumulation rates into tumor tissues. (4) Insolubility in water and low solubility in common solvents, leading to intermolecular aggregation and reduced optical properties. (5) Long half-life and skin accumulation, causing skin phototoxicity.^151–153^ With the advent of second-generation PSs, researchers have identified compounds with high singlet oxygen yield, including porphyrin-based (chlorins,^154^ phthalocyanines,^155^ texaphyrins,^156^ and similar macrocyclic molecules) and non-porphyrin-based PSs (phenothiazinium dyes^157–159^ and cyanines^123^). Several strategies to enhance the water solubility of PSs have been developed, including (1) Introduction of functional groups (nitrogen, carboxyl, ethylenediaminetetraacetic acid, etc.) to the main skeleton.^160,161^ (2) Conjugating with water-soluble moieties (amino acids, peptides, or metals).^155,162^ (3) Incorporating metal ions (zinc, platinum, indium, etc.).^163^ However, second-generation photosensitizers still face challenges that distance them from being ideal photosensitizers, including (1) Poor active targeting. (2) Less selective accumulation in target tissues. (3) Singlet oxygen quenching effects caused by π–π accumulation (known as ACQ). (4) Skin phototoxicity. (5) Rapid excretion and metabolic inactivation. There is still a pressing need to develop new PSs with more favorable photophysical properties for clinical applications. Additionally, ideal PTAs for PTT should exhibit higher photothermal conversion efficiency (PCE), absorption profiles that do not overlap with the tumor background and effective accumulation within tumors. These are among the factors limiting the clinical effectiveness of PTT.

**Fig. 4:**
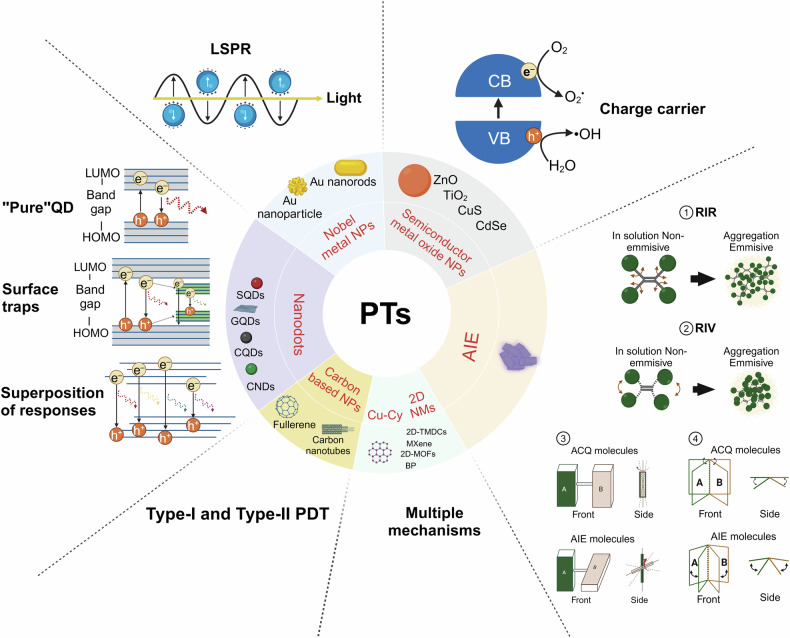
Classification of seven different types of nano-phototherapeutic agents, specific examples, and their corresponding mechanisms of action illustrated. LSPR localized surface plasmon resonance. CB conduction band. VB valence band. QD quantum dot. LUMO lowest unoccupied molecular orbital. HOMO highest occupied molecular orbital. PDT photodynamic therapy. ACQ aggregation-caused quenching. AIE aggregation-induced emission. RIR restriction of intramolecular rotation. RIV restriction of intramolecular vibrations. The figure was created with BioRender.com

With the advancements in nanotechnology, the utilization of nanomaterials, either as carriers or as PTs, has significantly propelled the development of phototherapy. Nanoparticles have gained widespread attention due to their ability to address the inherent drawbacks of traditional PTs: (1) Diverse properties: Nanomaterials vary in optical and electrical properties, with significant tunability based on size and shape, making nano-PTs promising for cancer therapy. (2) Near-infrared excitation: Nano-PTs, in native or modified states, can be stimulated by near-infrared light, which addresses limited light penetration in deep tissue tumor treatment. The NIR-II window surpasses the NIR-I in terms of deeper tissue penetration and reduced light scattering. Additionally, the skin’s maximum permissible exposure to NIR-II window light is significantly higher, making NIR-II excitation a current focal point of research in phototherapy.^164,165^ (3) Enhanced photostability: Nano-PTs display improved photostability, minimizing photobleaching over time. (4) Surface functionalization: Physical and chemical surface functionalization enhances water solubility, stability, drug-binding capacity, precise tumor targeting, and dispersibility of nanomaterials. (5) Passive and Active Targeting: Nanomaterials utilize passive accumulation in tumors via the enhanced permeability and retention effect (EPR), surpassing the targeting limitations of traditional PT. Through biological ligand-receptor interactions, nanoparticles actively target tumor or tumor microenvironment (TME) cells, augmenting PTs targeting and retention in tumors. (6) Drug delivery: Nanomaterials’ high specific surface area enables effective drug delivery. When combined with phototherapy and chemotherapy drugs, they enhance antitumor efficacy compared to phototherapy alone.^166–170^ Due to the overlapping mechanisms of action in PDT and PTT, the development of nanomaterial-based phototherapies has revealed that most materials not only serve as effective PSs but also as potent PTAs. This chapter delineates, based on their operational principles: (1) Semiconductor oxide nanomaterials that achieve phototherapeutic effects through band-edge electron-hole recombination; (2) Carbon-based nanomaterials; (3) Various types of nanodots; (4) Noble metal nanoparticles primarily utilizing the LSPR effect; (5) Two-dimensional (2D) nanomaterials, noted for their unique advantages; (6) Aggregation-induced emission (AIE) nanomaterials that utilize photoluminescence for energy transfer; and (7) Novel copper-cysteamine nanoparticles that exert photodynamic therapy (PDT) effects through multiple mechanisms (Fig. 4). Additionally, this section will explore the advantages of nanocarrier systems and their design for targeted delivery.

### Semiconductor-based nanoparticles

Semiconductor photocatalytic technology has garnered significant attention in PDT for cancer, particularly in hypoxic tumors, as it can decompose H_2_O into ROS under UV irradiation. Various semiconductor photocatalysts have been developed, including TiO_2_,^171^ ZnO,^172^ g-C_3_N_4_,^173^ bismuth-based compounds,^174^ and others. When a semiconductor material is irradiated with light exceeding its bandgap energy, electrons (e^−^) transition from the valence band (VB) to the conduction band (CB), leaving behind a positive electron-hole (h^+^) in the VB. Some of the electron-hole charge carrier pairs (h^+^_VB_ + e^−^_CB_) can recombine and undergo charge annihilation to dissipate excess energy, thereby reducing the efficiency of the photocatalytic process.^175^ The remaining charge carriers that do not undergo annihilation can migrate to the catalyst surface and undergo a secondary reaction with reactants adsorbed on the surface. The e^−^_CB_ reacts with O_2_ to generate superoxide radicals, while the h^+^_VB_ reacts with water to produce hydroxyl radicals^176^ (Eqs. 1 and 2).

1 $${e}_{{CB}}^{-}+{O}_{2}\to {O}_{2}^{\cdot }$$

2 $${h}_{{VB}}^{+}+{H}_{2}O\to \,\cdot\, {OH}+{H}^{+}$$

This typical Type-I PDT reaction, independent of oxygen molecules, renders it potentially widely applicable in hypoxic tumors. However, TiO_2_ has a large bandgap (3.2 eV), and only UV light (<380 nm) can activate it. The limited penetration of UV light restricts its effectiveness in treating deep tumors. Additionally, the recombination rate of electron-hole charge carrier pairs also hampers overall efficiency. Researchers have enhanced its physicochemical characteristics by modifying its surface, primarily through two strategies: photosensitization and doping.

Photosensitization involves the technique where photoinduced electrons are directly or indirectly injected from the surface of a complex onto the CB of a semiconductor material, or where holes are directly or indirectly injected onto the VB. Inorganic sensitizers, organic dyes, and coordination metal complexes are commonly used sensitizers in research. Studies have shown that multi- and co-sensitization (i.e., coupling a semiconductor material with two or more sensitizers) can maximize the efficiency compared to single sensitization.^177^ For instance, Xie et al. significantly improved charge separation by coupling CdSe and BiVO_4_ with TiO_2_.^178^ Chang et al. loaded the methylene blue dye into TiO_2_ nanocrystals, successfully expanding its photo-responsive region to 660–900 nm and significantly suppressing the recombination rate of electron/hole pairs.^179^

Doping refers to the process of introducing appropriate atoms/ions into the host lattice to create a hybrid material with new properties. Nanomaterials with optical activity are highly sensitive to doping. TiO_2_ is an effective host that can be doped with various metal ions, non-metal dopants, etc. Doping not only allows dopants to capture excited electrons, reducing the electron-hole recombination rate, but also decreases the TiO_2_ bandgap. Bai et al. utilized the reduced bandgap characteristic of iron-doped TiO_2_ to prepare Fe-TiO_2_-Polyethylene glycol nanodots (Fe-TiO_2_-PEG), significantly improving the tumor suppression effect.^180^

Semiconductor-based nanomaterials have also become a research focus as PTAs due to their strong absorption in the NIR region. Examples include metal sulfides (e.g., CuS, CdS) and transition metal oxides (e.g., MoO_3-x,_ WO_3-x_), as well as transition metal selenides (e.g., Cu_2-x_Se, CdSe). CuS, a p-type semiconductor with a bandgap ranging from 1.1 to 2.0 eV depending on its composition and crystallinity, exhibits strong NIR absorption capabilities due to LSPR effects.^181^ For instance, Wei and colleagues engineered a biomimetic nano-immunostimulator by integrating CuS nanodots with 4T1 tumor cell membranes onto a zeolitic imidazolate framework-8 (ZIF-8) structure. Leveraging the NIR-II photothermal effect of CuS and the acid-responsive nature of ZIF-8, this complex rapidly induced Zn^2+^ overload within tumor cells, disrupting their metabolic flux and alleviating resistance to PTT. Concurrently, this approach induced immunogenic cell death and initiated an immune cascade response, achieving synergistic photothermal immunotherapy.^182^

Transition metal oxides, characterized by their unique outer-d electron properties, have also emerged as promising candidates for PTT due to their LSPR capabilities.^183^ For example, Guo et al. synthesized amorphous MoO_3-x_ nanosheets with LSPR features by introducing Mo atoms into monolayers of MoO_3_ through a hydrothermal process, achieving an exceptional photothermal conversion efficiency of 61.79%.^184^ The d–d transitions of Cu ions confer strong NIR absorption to Cu_x_Se nanoparticles, which remain unaffected by the surrounding environment even during in vivo delivery. Under NIR irradiation, Cu_x_Se nanoparticles generate localized high temperatures, suitable for PTT applications.^185^ Wang and colleagues developed a Cu_2-x_Se/Bi_2_Se3@PEG (CB3@PEG) nano-heterostructure via a cation exchange process. Under NIR excitation, this structure produces hydroxyl radicals and singlet oxygen, while achieving a high photothermal conversion rate (60.4%), facilitating synergistic therapy.^186^

### Carbon-based nanomaterials

Carbon-based nanomaterials, encompassing fullerene, 0D carbon nanodots, 1D carbon nanotubes, 2D graphene, and oxidized graphene, have garnered extensive research interest in the field of both PDT and PTT for cancer, owing to their unique optical, mechanical properties, diverse chemical functionalities, and excellent biocompatibility.^187,188^ Fullerene, a molecule composed entirely of carbon, exists in various forms such as hollow spheres and ellipsoids, including C60 and C70, characterized by high symmetry.^189^ Compared to traditional tetrapyrrole-based photosensitizers, fullerene is noted for its high photostability, minimal photobleaching, and highly efficient ROS generation.^190^ Studies have demonstrated that fullerene can generate singlet oxygen via an energy transfer mechanism (Type-II) and produce superoxide radicals, hydroxyl radicals, and other ROS through an electron transfer mechanism (Type-I). It predominantly produces Type-I photoproducts, suggesting its potential efficacy in hypoxic tumors.^191^ However, pristine C60 is not an ideal PS due to its weak visible light absorption, high hydrophobicity, and inherent toxicity. These limitations can be addressed through two main modification strategies: cyclopropanation (Bingel reaction) and cycloaddition azomethine ylides (Prato reaction). The Prato reaction can introduce peptides or PEG conjugates onto fullerene, yielding water-soluble fullerene derivatives.^192^ Conversely, the Bingel reaction can functionalize fullerene with two different groups, allowing it to be derivatized with two different molecules.^193^ Carbon nanotubes (CNTs), composed of single or multi-walled tubes of sp^2^-bonded carbon sheets, are highly efficient absorbers in the NIR spectrum, rapidly converting electron excitation into molecular vibrational energy to generate heat.^194^ Consequently, they were among the first carbon-based nanomaterials utilized for PTT and have since been extensively studied. Zhao et al. reported a functionalized CNT delivery system loaded with therapeutic siRNA for tumor-targeted PTT and concurrent gene therapy. Under NIR excitation, this system exhibited excellent photothermal effects and high antitumor activity, completely suppressing tumor growth.^195^

### Nanodots

It is important to note that, aside from the distinct optical performance advantages associated with different material types, the size of nanoparticles also imparts varied optical properties. In recent years, quantum dots (QDs) with tunable photoluminescence (PL) properties have found extensive applications in areas including drug delivery, bioimaging, photodynamic therapy, photothermal therapy, and photocatalysis. Initially, QD referred to semiconductor nanoparticles confined within quantum dimensions (typically a few nanometers, less than the Bohr exciton radius), meaning excitons are confined in a dimension that results in quantized energy states.^196^ To avoid terminological confusion, current research collectively refers to all types of nanodots exhibiting quantum confinement as QDs, including semiconductor-based dots (SQDs, typically 2–10 nm in size) and carbon-based dots (CDs, generally less than 10 nm in size with a thickness around 0.5–5 nm, depending on the preparation method). Carbon-based dots are further subdivided based on the arrangement of carbon atoms and crystal structure into carbon nanodots (CNDs), carbon quantum dots (CQDs), and graphene quantum dots (GQDs). SQDs are perfect spherical nanocrystals of metal atoms. GQDs are π-conjugated carbon nanosheets derived from graphene-based materials, ranging from 2 to 20 nm in size. Based on a crystalline structure of carbon materials (including carbon nanotubes) as precursors, nuclei composed of sp2 and sp3 hybrid carbon, known as CQDs, have been developed. Amorphous quasi-spherical nanodots primarily composed of sp^3^-bonded carbon are referred to as CNDs.^197^

Based on their distinct structural cores, these nanoparticles exhibit unique properties, yet also share many common features. The PL mechanisms of such dots can generally be categorized into three major types: (1) “Pure” QD, devoid of defects or impurities, where PL originates from electron-hole pair recombination, also known as the highest occupied molecular orbital-lowest unoccupied molecular orbital (HOMO-LUMO) transition. Here, the PL characteristics are entirely determined by quantum confinement effects, exhibiting size dependency with generally narrow PL emission bands (FWHM < 40 nm). It is currently believed that SQDs fall into this category of PL origin. (2) In the presence of trap states within the bandgap—due to impurities, surface defects, functional groups, or adsorbed molecules—excited electrons and/or holes may be captured, with subsequent recombination releasing lower energy. This type of PL emission is influenced both by intrinsic quantum effects and by surface trap states, leading to excitation-dependent characteristics. Most CQDs and GQDs fall under this category. (3) The third PL mechanism, which is not governed by quantum confinement, arises from the superposition of responses of fluorescent groups or emitting functional groups located on the surface of the nanoparticles. This fluorescence behavior is more akin to what is observed in metal nanoclusters. Typically, CNDs follow this PL mechanism, where the overlapping of multiple emission centers results in very broad emission bands. When the surface-emitting functional groups are quenched, their PL can be completely suppressed.^197–199^

#### SQD

SQD materials are typically categorized into cadmium chalcogenides (II–VI group semiconductors), lead chalcogenides (IV–VI group semiconductors), non-heavy metal compounds (III–VI, I–III–VI, and I–VI group semiconductors), and silicon (IV group semiconductors).^200^ The selection of different material particles is based on the required optical properties, with CdSe^201^ and CdTe^202^ being notably prominent in life science research. The distinctive optical properties of SQDs mainly include: (1) Large transition dipole moment, providing strong light absorption capability, enabling them to act as energy donors, transferring energy to oxygen molecules and generating ROS or heat. (2) Broad excitation spectra, narrow emission spectra, and large stokes shifts. (3) Tunable absorption and emission spectra based on the size of the SQDs, known as the quantum size effect,^203^ allowing precise tuning from UV to NIR spectra. (4) Larger molar extinction coefficient compared to traditional organic photosensitizers^204^; (5) Enhanced photochemical stability with almost complete suppression of photobleaching when adequately surface-passivated.^205^ (6) Functionalization of the surface coating, conferring excellent water solubility and biocompatibility. While studies have shown the capability of QDs to undergo triplet energy transfer (TET) with directly contacting ground-state triplet oxygen (^3^O_2_) to generate ^1^O_2_, their ^1^O_2_ generation is relatively low. This may be attributed to carrier trapping and nonradiative carrier recombinations. The spin statistics also limit the fraction of SQD-^3^O_2_ contacts, contributing to the low ^1^O_2_ generation.^206^ Currently, the primary application of SQDs in PDT involves sensitizing the PSs through Förster resonance energy transfer (FRET) and/or electron transfer (eT) processes. For example, Martynenko et al. synthesized nanocomposites containing ZnSe/ZnS SQDs and the PS Ce6. In these nanocomposites, approximately 50% intracomplex FRET was detected, indicating an effective PS. In Erlich ascite carcinoma cells, these nanocomposites exhibited twice the cancer cell destruction ability compared to Ce6 alone.^207^ SQDs, such as those synthesized by Chen et al., exhibit outstanding photothermal conversion efficiencies. Specifically, the biologically synthesized Ag_2_Se quantum dots (bio-Ag_2_Se-CAT) demonstrate efficiencies of 75.3% at 808 nm and 51.7% at 1064 nm. These high efficiencies confirm their potential for PTT, which can alleviate thermal-induced oxidative stress.^208^

#### CDs

CDs offer several advantages over traditional SQDs, including better photostability, reduced photobleaching, and diminished blinking. They also boast lower toxicity and improved biocompatibility. Theoretical studies on the electronic structure of CDs reveal that due to the quantum confinement effect (QCE), as the size of the CDs increases, the HOMO and LUMO shift to higher and lower energies, respectively, narrowing the HOMO-LUMO gap. Moreover, covalent or non-covalent modifications introducing surface functional groups and heteroatom doping (including metal and non-metal ions) can enhance the photoluminescence performance of CQDs. Different functional groups can variably alter the energy levels of the HOMO and LUMO, thereby impacting the energy gap of CDs in distinct ways.^209,210^ Non-metal ion doping, such as S-doped CDs, effectively modifies the electronic structure by introducing S-related energy levels between the π-π* orbitals, altering the electron transition pathways and interband crossings.^211^ Metal ion doping enhances the photoluminescence quantum yield through the creation of emissive energy traps, which facilitate electron-hole recombination,^212^ or due to the presence of surface plasmon resonance (SPR) effects in metal nanoparticles.^213^ Collectively, the unique electronic and chemical structures of CDs can be finely tuned through adjustments in size, shape, surface functional groups, and heteroatom doping, opening broad prospects for their applications in biomedicine and optoelectronics.

Zhang et al. synthesized hRCDs using derivatives extracted from Hypericum perforatum, which have been demonstrated to generate superoxide anions through type-I PDT reactions and singlet oxygen through type-II PDT effects, inducing programmed cell death in tumor cells by activating the mitochondrial-mediated apoptotic pathway.^214^ Liu et al. developed a novel in situ immune-inducing hydrogel based on mannose-modified aluminum-doped amino carbon quantum dots (M/A-CD@Gel), designed for tumor PTT and delivery of immunoadjuvants (CpG-ODN). Studies confirm that M/A-CD exhibits excellent photothermal conversion rates and also serves as a carrier for immunoadjuvants, targeting the maturation of dendritic cells and enabling synergistic PTT and immunotherapy.^215^

### Noble metal nanoparticles

Noble metal nanomaterials, such as gold (Au) and silver (Ag), exhibit unique optical properties that enable the absorption of laser light, thereby exciting electrons from the ground state to an excited state. These electrons can release energy through nonradiative decay, manifesting as heat for PTT effects, or through radiative decay via electron transfer, generating ROS for PDT.^216^ The optical characteristics of these noble metals are highly tunable, influenced by their size, shape, and atomic configuration, offering significant advantages for cancer therapy applications utilizing PDT and PTT. In noble metal materials, larger nanoparticles (3–100 nm) exhibit strong LSPR effects. Upon irradiation with specific wavelengths of light, an electromagnetic field induces coherent oscillations of conduction band electrons and dipolar oscillations of the electric field, leading to energy transfer.^166^ The LSPR effect enables microstructures to generate enhanced photo-induced electric fields locally and absorb more incident photons. This process results in the production of high-energy hot electrons and an enhanced electromagnetic field. Hot electrons release their energy through electron-phonon relaxation processes, demonstrating exceptional photothermal conversion efficiency for effective PTT. The energy from LSPR can also be transferred to molecular oxygen to form singlet oxygen, thereby contributing to the PDT effect. Studies have demonstrated that for noble metal nanomaterials to effectively generate ^1^O_2_, it is essential to adsorb O_2_ on the surface of the noble metal materials. Furthermore, research suggests that the low-energy surface states of noble metal nanoparticles can efficiently transfer energy to molecular oxygen, while the high-energy surface states of the nanoparticles exhibit lower efficiency in transferring LSPR energy to molecular oxygen.^166^ The position and bandwidth of the LSPR resonance peak can be adjusted by altering the size, shape, and crystallinity of the nanomaterial.^217^ For example, gold nanorods are excited under near-infrared light (915 nm λ1) irradiation^218^; gold nanoshells can be excited under ultra-low dose (w150 mW/cm^2^) NIR-I (980 nm) irradiation^133^; and gold nanoechinus at wavelengths covering both NIR-I (650–950 nm) and NIR-II (1000–1350 nm), with extinction coefficients at both wavelengths greater than those of traditional organic PSs.^219^ A high extinction coefficient implies that less PTs, lower light intensity, and shorter irradiation time are required for deeper tissue cancer treatment. In contrast to large nanoparticles, ultrasmall gold nanoclusters (Au NCs) (less than 3 nm, 25–100 gold atoms) are precise atomic particles protected by metal-ligand (M-L) complexes. While they do not exhibit SPR, they achieve electron transitions through HOMO-LUMO.^220^ They are typically represented by [Au*n*(L)*m*]*q*, where *n*, *m*, and *q* represent the number of gold atoms, the number of ligand atoms, and the net charge of a single cluster, respectively.^221^ The optical properties of Au NCs strongly depend on parameters such as the number of atoms, type and number of ligands, and their length.^222^ In summary, the high tunability of noble metal materials positions them as promising candidates in phototherapy applications. Additionally, compared to traditional organic PTs, noble metal nanoparticles exhibit greater stability under irradiation (as organic dyes tend to decompose under strong irradiation, leading to a decrease in reaction rate) and demonstrate good biocompatibility. Tao et al. synthesized gold nanoparticles (AuNO) by adjusting the ratio of cetyltrimethylammonium chloride (CTAC) to cetyltrimethylammonium bromide (CTAB), demonstrating significant LSPR effects and high photothermal conversion efficiency (PCE of 47.68%) within the NIR-II window. After coating with mesoporous polydopamine (mPDA), the PCE was further increased to 66.17%.^223^ Meanwhile, Yang et al. developed a theranostic probe based on ultrasmall gold nanoclusters (Au44MBA26-NLG) capable of performing NIR-II photoluminescence imaging for deep tissue analysis. This probe also leverages its photothermal properties for synergistic PTT and PDT treatment of tumors. Additionally, the probe is conjugated with an immune checkpoint inhibitor, 1-cyclohexyl-2-(5H-imidazo[4,5-c]pyridine), thereby facilitating T cell proliferation and activation. This enhancement in systemic antitumor T cell immunity effectively suppresses both primary tumor growth and distant metastases.^224^

### 2D nanomaterials

Two-dimensional (2D) nanomaterials (2DnMat) exist in a sheet-like structure with a thickness of a single or a few atoms, and lateral dimensions ranging from a few nanometers to several hundred nanometers. This unique structure endows them with enhanced chemical, electronic, and optical properties.^225^ Studies have demonstrated that 2D nanomaterials can improve the pharmacokinetic properties of drugs, increase their accumulation in tumors, and enhance their physiological utilization.^226^ In recent years, various distinctive 2D nanomaterials have shown tremendous potential in energy storage, catalysis, biomedical applications, and environmental applications. Current advancements in 2D nanomaterials include graphene-based materials (GBM), 2D layered transition metal dichalcogenides (2D-TMDCs), 2D layered double hydroxides (2D-LDH), 2D metal-organic frameworks (2D MOFs), transition metal oxides (TMOs), transition metal carbides, nitrides, and carbonitrides (MXene), and black phosphorus (BP). Since graphene-based materials, along with carbon quantum dots and related nanomaterials, have been extensively discussed in the previous section, they will not be elaborated further here.

2D-TMDCs structurally consist of a layer of transition metal atoms sandwiched between two layers of chalcogen atoms, typically represented by the formula MX_2_, where M denotes a transition metal (such as Ti, Re, Pd, or Pt) and X represents a chalcogen element (S, Se, or Te).^227^ TMDCs possess an exceptionally high surface area-to-volume ratio and outstanding optical properties, including excellent fluorescence, strong NIR absorption, and high photothermal conversion efficiencies, making them suitable for photothermal applications.^228,229^ Representative 2D-TMDCs include MoS_2_, WS_2_, and MoSe_2_, among which MoSe_2_ nanostructures are currently regarded as some of the most promising NIR photocatalysts due to their appropriate bandgap (1.33–1.72 eV) and substantial NIR absorption. For instance, Wang et al. utilized the superior NIR absorption properties of MoSe_2_ to construct MoSe_2_/Bi_2_Se_3_ nanosheets, achieving a narrower bandgap (1.17 eV) and stronger NIR absorption. This enhancement in NIR absorption facilitated the increased generation of ROS through the electron-hole principle, simultaneously improving the photothermal conversion efficiency (59.3%) and achieving synergistic photodynamic and photothermal antitumor effects.^230^

2D-LDHs are characterized by their pH-sensitive biodegradability, outstanding biocompatibility, and tailor-made chemical composition and structure, offering vast application prospects.^231^ The general formula for 2D-LDHs is [M^2+^_1-x_M^3+^_x_(OH)_2_]^x+^(A^m^^−^)_x/m_∙nH_2_O, where M represents a divalent or trivalent metal ion in oxidation state, and A denotes an interlayer anion.^232^ For instance, Yang et al. demonstrated that by loading 2D CoCuMo LDH nanosheets on Lactobacillus acidophilus probiotics (LA), they could enhance the production rate of singlet oxygen under 1270 nm laser irradiation, leading to complete apoptosis and eradication of tumor cells.^231^

2D MOFs represent a novel category of porous nanomaterials composed of metal nodes connected by organic ligands. Due to their superior functional groups, adjustable porous structure, and efficient biodegradation coupled with rapid renal clearance, 2D MOFs ensure excellent biocompatibility and are thus advantageous in PDT, PTT, drug delivery, and imaging applications.^233^ The functionalities of 2D MOFs in phototherapy can be categorized into two types: intrinsic phototherapy MOFs, which directly serve as PS or PTA without the need for additional PS or PTA, and MOFs modified by phototherapeutic agents. An example of an intrinsic phototherapy MOF is the Prussian blue-based MOF (PB), which is one of the oldest synthetic MOFs and has been extensively studied for its PTT applications. Its structure features iron ions (Fe(II) and Fe(III)) coordinated with carbon and nitrogen atoms, forming a face-centered cubic structure.^234^ Alternatively, MOFs can serve as carriers for various PS and PTA, synthesized into composites through methods such as polystyrene encapsulation, surface attachment, and core-shell structuring. Zheng et al. provided a comprehensive and insightful review of the developments of MOFs in phototherapy applications.^235^

MXenes are composed of transition metal carbides, nitrides, or carbonitrides, typically represented by the formula M_n+1_X_n_T_x_ (*n* = 1–4), where M denotes a transition metal (e.g., Ti, Sc, Zr, Mo), X is carbon or nitrogen, and Tx indicates surface terminations bonded to the external layers of M, such as O, OH, F, Cl. Since their initial isolation in 2011, MXenes have demonstrated promising prospects in biomedical applications due to their excellent optical, mechanical, and metallic conductive properties, inherent magnetism, and the presence of functional groups on their surfaces.^236^ Carbon doping in the transition metal lattice imparts MXenes with ultrahigh conductivity and semi-metal-like characteristics, leading to the generation of LSPR effects under incident light stimulation. Unlike other plasmonic materials, the LSPR effect in MXenes is not size-dependent but rather related to the surface termination groups.^237^ For example, the 2D Ti_3_C_2_ MXene nanosheets designed by Zhao et al. enable synergistic PDT and PTT, amplifying the cascading catalytic treatment effects to inhibit tumor growth.^238^

Another current hotspot for 2D materials in PDT is BP. BP as a new type of 2D semiconductor material, is an ultra-thin 2D nanosheet composed of folded layers of phosphorus under weak van der Waals forces.^239,240^ Its bandgap is strongly thickness-dependent (ranging from 0.3 eV for bulk to 2.0 eV for monolayer), and BPs with different lateral dimensions can be obtained through strategies such as liquid exfoliation, making BP exhibit a wide range of optical absorption.^241^ In addition, the strong near-infrared light absorption and high photothermal conversion efficiency of BP make it highly attractive for cancer PTT^242^ and PDT^243^ therapy. Unlike other nano-PT, BP NPs can degrade into harmless phosphate in a physiological environment, thus having high biocompatibility and low cell toxicity. Moreover, this process can be regulated by light of different wavelengths.^244^ Wang et al. first demonstrated that BP NPs undergo type-II PDT reaction under irradiation of 660 nm laser, consuming surrounding oxygen and generating singlet oxygen, thereby inhibiting tumor growth both in vitro and in vivo.^243^ Meanwhile, as a 2D nanomaterial, its ultrahigh surface area-to-volume ratio makes it an ideal candidate for drug delivery. When combined with its photodynamic properties and drug-carrying capabilities, it can more effectively kill cancer cells. However, BP is prone to reacting with oxygen and water, which leads to poor stability. Physicochemical modification of its surface is an effective method to prevent degradation.

In addition, researchers in the field of catalysis have shifted their focus from nanoparticle catalysts to single-atom catalysts (SACs). SACs are composed of single atomic motifs and support substrates, representing a current research hotspot in the fields of photo-, electro-, thermo-, and enzyme catalysis. With low coordination numbers, unique coordination environments, high atomic efficiency, and highly uniform catalytic centers, SACs exhibit exceptional properties in catalysis.^245,246^ Differing from conventional catalysts featuring nanoparticles and nanoclusters, single-atom motifs offer higher precision and flexibility in regulating the crystalline, coordinative, and electronic structures of catalysts. The support substrate not only stabilizes the single-atom motifs but also plays a crucial role in determining the catalytic activity and selectivity of the system.^247^ Among the existing SAC systems, notable examples include Xenes-SACs based on 2D monoelemental materials,^248^ such as phosphorene, arsenene, bismuthene, selenene, and others (mentioned before). Additionally, SACs have been developed using 2-dimensional transitional metal dichalcogenides (2D-TMDs).^247^ Currently, there is limited research on the application of SACs in PDT. Wang et al. reported a novel system comprising a Fe (III) porphyrin-containing metal-organic framework with single-atom Fe sites for both PDT and PTT. This system generates sufficient energy and ^1^O_2_ after being excited by NIR.^249^

### AIE

AIE refers to a unique phenomenon where a molecule exhibits no fluorescence in its dispersed state but emits intense fluorescence in its aggregated or solid state. This is opposite to the ACQ phenomenon, where fluorescence is quenched upon aggregation.^250^ Luminogens possessing AIE properties are referred to as AIEgens. Therefore, AIEgens has become a key solution to overcome the ACQ in traditional PTs. The current mainstream view is that the AIE phenomenon is mainly attributed to the restriction of the intramolecular motion (RIM) process.^251–253^ According to the different structures of the AIE system, RIM can be divided into the restriction of intramolecular rotation (RIR) occurring in helical-shaped molecules through single bonds (Fig. 3) and the restriction of intramolecular vibrations (RIV) occurring in shell-shaped molecules through bendable flexure (Fig. 3). Generally, AIE arises because rotor-carrying luminogens undergo low-frequency twisting and torsional motion in dilute solutions, and these rotation or vibration results the very fast non-radiatively decay of singlet excited states. However, in the aggregate state, these kinds of intramolecular motions are restricted due to the physical contact, resulting in the opening of the radiative decay pathway.^254^

In fact, any luminescent molecular cluster undergoes intramolecular rotation, but not all luminescent molecular clusters exhibit AIE characteristics. This is because, unlike traditional ACQ PTs, AIEgens possess different geometric planarity, conformational flexibility, and intramolecular motion. For helical-shaped molecules in traditional ACQ PTs, the chromophoric units are aligned in an almost parallel fashion, allowing the π-electron clouds of the chromophores to overlap and cross each other, giving the molecule a pseudo-double bond character. This results in the maximum electronic conjugation and minimum potential energy, as well as reduced intramolecular rotation due to the presence of rotatable C-C bonds. In contrast, the chromophoric units in AIE molecules are twisted out of plane due to steric effects, leading to reduced overlap of the π-electron clouds of the chromophores. In this case, the C-C bonds between the units do not restrict intramolecular rotation, and the strong vibrational motion dissipates a large amount of energy, resulting in reduced emission from the luminogens in the solution. When the luminescent molecules aggregate, the restriction on intramolecular vibrations leads to highly efficient emission from the aggregates. The probability of ISC from the singlet state to the lowest excited triplet state increases in the aggregates, leading to the manifestation of the AIE effect and subsequent photodynamic reactions (Fig. 3). Similar to the rotational model, whether molecular vibration models of other molecules exhibit AIE characteristics largely depends on their conceptual flexibility and vibrational amplitude. Figure 3 illustrates that in ACQ molecules, two luminescent units A and B are coplanar, and the A-B system exhibits maximum π-electron conjugation, resulting in a rigid conformation and suppressed internal molecular vibrations. Although A and B still undergo small oscillations, they are insufficient to prevent radiation decay. In contrast, in AIE molecules, the connection between A and B is non-planar, with minimal overlap of their π electron clouds. The π-electron conjugation weakens, allowing almost unrestricted internal molecular vibrations and greater vibrational amplitudes. Consequently, AIE effects can be observed (Fig. 3).^254^

Currently, the design of AIE-based PTs focuses mainly on two approaches: (i) enhancing the performance of AIEgens themselves to increase their chance of sensitizing surrounding oxygen molecules to generate singlet oxygen, and (ii) introducing AIE moieties into other PTs to transform them into AIE PTs. Due to space limitations, we will focus on the first approach, which involves improving the performance of AIEgens. Promoting the ISC of AIEgens is the key to designing AIE PS while promoting ISC depends on reducing the energy gap between the singlet and triplet states (i.e. decreasing ΔEST). Introducing donor (D) and acceptor structures (A) into AIEgens is the main strategy to improve AIE PS performance, and the type, quantity, distance, and torsion angle of the D-A group can all alter the photosensitization of AIE PTs.^255^ For example, the acceptor TCAQ has a stronger electron-withdrawing ability than DC, resulting in a lower ΔEST.^256^ Introducing a benzene ring (TPEDC2)^256^ or a thiophene group (TP8)^257^ into TPE-red can increase the D-A distance and thus reduce ΔEST. Studies on the D-A even-odd effect have found that molecules with more A units than D units are more suitable for efficient PTs.^258^ It is worth mentioning that two major factors, poor light penetration, and tumor hypoxia, continue to limit the clinical application of AIE PSs. In contrast to light, microwaves exhibit strong tissue penetration and can utilize the microwave thermal ablation effect to increase tumor blood flow, thereby enhancing tumor oxygen content. Therefore, Nil Kanatha Pandey and colleagues used two AIEgens, TPEPy-I and TPEPy-PF6, as microwave sensitizers to generate ROS and effectively kill tumor cells under microwave irradiation.^259^ Both TPEPy-I and TPEPy-PF6 share common characteristics, consisting of a TPE segment working as a donor (D), a thiophene fragment working as a π-bridge, and a cationic pyridinium moiety working as an acceptor (A). Consequently, they possess a strong charge-transfer feature and efficient intersystem crossing (ΔEST S1-T3: −0.22 eV), enabling excitation by relatively low-energy microwaves (10^−^^3^ eV).^260^ Research demonstrated that under microwave irradiation (2w-10w), both TPEPy-I and TPEPy-PF6 can significantly produce singlet oxygen (^1^O_2_) in a dose-dependent and irradiation time-dependent manner. In Hela cells, under 10w microwave irradiation for 1.5–2 min, TPEPy-I and TPEPy-PF6 exhibit significant cytotoxic effects. Cui and colleagues meticulously controlled intramolecular interactions and molecular structural distortions to successfully synthesize AIEgens-DHTDP. This compound concurrently achieves NIR-II fluorescence emission and high-efficiency photothermal conversion, guiding PTT through combined NIR-II fluorescence and photoacoustic imaging.^261^

### Copper-cysteamine (Cu-Cy)

Cu-Cy represents a prototypical metal complex structure where copper binds with sulfur ligands in proteins and is widely present in various enzymes. Cysteamine, a non-toxic and water-soluble small molecule, facilitates the formation of this classical Cu-Cy complex, which serves as an accessible model compound for advancing fundamental knowledge of copper-containing enzymes, despite its lack of luminescence.^262,263^ In a groundbreaking development, the research Chen Wei team introduced a novel type of Cu-Cy complex, specifically Cu_3_Cl(SR)_2_ (R=CH_2_CH_2_NH_2_).^264^ In this innovative complex, both the thiol and amine groups bond with copper ions, leading to strong luminescence.^264,265^ This luminescent Cu-Cy complex has been experimentally verified to generate ROS under various stimuli, including UV radiation,^264,266^ X-rays,^267–269^ microwave radiation,^270,271^ and ultrasound.^272^ This discovery provides a fresh perspective on the development of new-generation PSs.

The primary advantage of this PS lies in its capability to generate ROS upon activation by X-rays, ultrasound, or microwave radiation. This unique property facilitates the selective eradication of cancer cells or viruses, making it suitable for the treatment of both superficial cancers and deep-seated tumors.^266,269,273,274^ Chen Wei’s team has substantiated the efficacy of Cu-Cy-mediated X-ray-induced photodynamic therapy (X-PDT) across various cell lines, including HepG2, Li-7, SK-HEP-1, and 4T1. Furthermore, they have demonstrated its significant anticancer effect in mouse and rabbit models, while also establishing the safety of Cu-Cy under non-intervention conditions. In a more comprehensive exploration of the molecular mechanisms underlying Cu-Cy-mediated X-PDT in treating tumor phenotypes, Chen Wei’s team uncovered noteworthy findings. They observed that, during x-PDT treatment, the expression of the proliferating cell nuclear antigen (PCNA), associated with the proliferative phenotype, was minimized, while the expression of E-cadherin (E-cad), linked to the cell migration phenotype, was elevated. This suggests a substantial inhibitory effect on tumor proliferation and metastasis. Additionally, their research revealed that Cu-Cy promotes the formation of the body’s antitumor immune response. In particular, the Cu-Cy+X-ray treatment group exhibited a significant increase in CD^4+^ and CD^8+^ T cells in the spleen. Moreover, the proportion of CD^8+^ T cells and NK cells in the tumor tissue saw a notable elevation, and M2 macrophages were significantly reduced. In summary, Cu-Cy-mediated PDT induces a robust antitumor immune response by stimulating DC cell maturation, activating CD^4+^ T, CD^8+^ T, and NK cells, and inhibiting M2 macrophages in the TME.^275^

Cu-based materials are recognized as catalysts with the ability to undergo Fenton-like reactions with H_2_O_2_, and their reaction rate is notably higher than that of Fe-based materials.^276^ Furthermore, research has indicated that the reaction rate of Cu^1+^ is nearly 22 times greater than that of Cu^2+^ (as shown in equations 3 and 4^277^).

3 $${{Cu}}^{1+}+{H}_{2}{O}_{2}\to {{Cu}}^{2+}+\,\cdot\, {OH}+{{OH}}^{-}\left(k=1\times {10}^{4}{M}^{-1}{S}^{-1}\right)$$

4 $${{Cu}}^{2+}+{H}_{2}{O}_{2}\to {{Cu}}^{1+}+\,\cdot\, H{O}_{2}^{-}+{H}^{+}\left(k=460{M}^{-1}{S}^{-1}\right)$$

Cu-Cy NP, as a novel structure that replaces Cu^2+^ with Cu^1+^, possesses an additional advantage. In addition to activation by various irradiation methods, it has been demonstrated that Cu-Cy NP can leverage the elevated H_2_O_2_ levels within the tumor microenvironment to undergo Fenton-like reactions. This process results in highly efficient ROS, enabling the selective targeting and eradication of tumor cells while causing minimal harm to normal cells.^278^ This dual activation mechanism enhances the specificity of Cu-Cy NP in targeting cancerous tissues, presenting a promising avenue for effective and selective cancer treatment strategies.

### Synthetic nanocarriers system

Thus far, we have introduced seven developed nano-PTs with unique physicochemical properties.

These nano-PTs can serve as PS themselves, overcoming various limitations of traditional PTs, and thereby enhancing the efficacy of phototherapy for cancer. However, with the development of nanomaterials and synthesis technologies, constructing complex and efficient PT delivery systems has also provided a breakthrough in overcoming the limitations of traditional PDT. In this section, we will discuss the synthetic nanocarrier delivery systems and the current major strategies for tumor targeting. Nanocarrier systems can be improved in their physicochemical properties, reduced side effects, and enhanced efficacy through design, synthesis, and modification. The main advantages of nanomaterial drug loading and delivery systems include: (1) Large surface area-to-volume ratio: This makes nanocarriers highly suitable for drug loading and delivery; (2) Protection from enzymatic degradation. (3) Tumor targeting potential: Nanocarriers have significant tumor targeting potential, including passive and active targeting abilities, as well as bioorthogonal effect. This can significantly enhance the accumulation of PTs in tumors and reduce unnecessary damage to healthy cells. (4) Controlled and continuous delivery: Nanocarriers can be designed to deliver PTs continuously and in a controlled manner to the target site, for example, by designing a tumor response release system. (5) Enhanced optical and electrical properties: The optical and electrical properties of the nanocarriers themselves can enhance the PDT and PTT of the PTs they carry.

The commonly encountered synthetic nanocarrier systems include: (1) PEG: PEG enhances the solubility of PTs, reduces uptake of PTs by macrophages, diminishes the interaction of PTs with lipoproteins in the bloodstream, and increases stability. For instance, in preclinical studies with the PS mTHPC, its coupling with PEG resulted in a two-fold extension of half-life in mouse plasma compared to free mTHPC. It exhibited better accumulation in colon cancer xenografts and lower concentration in the liver.^279^ (2) Macromolecular-based nanocarriers: These include solid polymeric nanoparticles, which are colloidal-stable nanostructures typically composed of biocompatible and biodegradable components. After cellular uptake, they can release drugs through biodegradation. The most commonly used polymer is Poly(lactic-co-glycolic acid) (PLGA), which, upon hydrolysis, produces lactic acid and glycolic acid, both exhibiting low systemic toxicity.^280^ (3) Protein-based nanoparticles: Human serum albumin (HSA) is one of the most renowned natural carriers for PTs due to their superior biocompatibility, biodegradability, and non-antigenicity.^281^ (4) Lipid-based nanoparticles: Classic liposomes, composed of phospholipids, are lipid bilayer nanocapsules capable of loading hydrophobic compounds within the lipid bilayer. Being a major component of biological membranes, they easily integrate into membrane structures, facilitating drug release upon fusion with biological membranes.^282^ (5) Extracellular vesicles (EVs): These are natural nanoparticles released by prokaryotic and eukaryotic cells. EVs resemble liposomes in size, morphology, and structure but possess a more complex bilayer structure, containing hundreds of lipid, protein, and carbohydrate types, as well as surface-related molecules. In various pathological and physiological processes, EVs primarily play a role in long-distance communication. Therefore, some suggest using EVs as natural nanocarriers for PTs.^283^ (6) Natural cell membrane (CM): Studies have demonstrated that natural CM can aid nanocarriers not only in evading the immune system but also in bypassing immune cells to exert effectiveness. Among them, cancer cell membrane (CCM) has become a hotspot due to its ability to target homologous tumors, with characteristics such as infinite replicative potential and immune evasion. Studies have shown that, after co-cultivation with tumor cells, CCM NPs exhibit uptake rates by tumor cells several times higher than ordinary nanoparticles.^284^ Additionally, CCM possesses a series of tumor-associated antigens (TAA) that can induce a specific immune response against homologous tumor antigens, thereby enhancing the effectiveness of immunotherapy.^285^

### Tumor targeting

Synthetic nanocarrier systems not only optimize the water solubility, stability, biocompatibility, and cellular uptake of traditional PTs but also enhance the tumor-targeting properties of PTs. This is a significant and important topic in the field of cancer therapy. Tumor targeting generally involves both passive targeting and active targeting strategies.

### Passive targeting

Passive targeting refers to the enhanced permeability and retention (EPR) effect, which results from the physiological differences between tumor and normal tissues. The malformed tumor vasculature with enhanced permeability and the lack of functional lymphatic drainage allow for the extravasation and retention of nanocarriers within the tumor.^286^ However, this passive EPR is influenced by various factors. For example, extracellular matrix components such as collagen and hyaluronic acid can form barriers that prevent nanocarriers from entering the tumor interstitium from the blood vessels, resulting in an inhomogeneous distribution of drugs within the tumor.^287^ The heterogeneity of the types of tumors, different individuals, and even between primary and metastatic tumors, results in varied clinical effectiveness of the EPR effect.^288^ Additionally, the size, shape, and surface physicochemical properties of nanoparticles can also affect the EPR effect: (1) Inorganic nanoparticles: These have higher drug delivery efficiency than organic nanoparticles. (2) Particle size: Within a particle size of 100 nm, smaller nanoparticles have higher drug delivery efficiency than larger ones. (3) Surface charge: Neutral surface-charged nanoparticles have higher drug delivery efficiency than nanoparticles with positive/negative surface charges. (4) Shape: Rod-shaped nanoparticles have higher drug delivery efficiency than spherical or plate-type nanoparticles.^289^

In recent years, there have been several studies on the use of pharmacological and physical strategies to enhance the EPR effect of nanomedicines. Pharmacological strategies include the use of drugs such as angiotensin agonists and antagonists,^290^ tumor necrosis factor-α (TNF-α),^291^ and nitric oxide-producing agents^292^ to regulate the VEGF signaling pathway, thereby enhancing the EPR-mediated drug accumulation in tumors. Physical strategies include: (1) Radiotherapy: This increases vascular leakiness via upregulation of VEGF and FGF expression.^293^ (2) Hyperthermia: Using radiofrequency, microwave, and focused ultrasound-induced hyperthermia to increase the tumor blood flow and vascular permeability.^294^ (3) Ultrasonic wave: Including microbubble oscillation and implosion to increase vessel permeability.^295^

### Active targeting

Active targeting refers to the molecular-level interaction between a nanosystem and target cells, usually mediated by specific biological ligand-receptor interactions.^296^ The advantages of active targeting include: (1) Increased accumulation: Enhanced accumulation of nanomedicines within the tumor; (2) Receptor-mediated endocytosis: Increased intracellular delivery through receptor-mediated endocytosis, which is crucial for therapeutic efficacy.^297^ (3) Target delivery: Delivery of the nanocarrier to cancer cells and achieving different therapeutic goals by targeting specific cells within the tumor microenvironment. The ligands currently used in research mainly include: (1) Ligands: such as folate (FA) that target growth factor receptors overexpressed in different cancers have become routine targets, and targeting FA has been shown to significantly enhance the internalization of cancer cells^298^; (2) Antibody engineering: Technologies such as monoclonal antibodies (mAbs), antibody fragments, and nanobodies have been used for targeting cancer cells with higher specificity. For example, studies have shown that mAb cetuximab has successfully achieved targeting of the EGFR, a kinase that is widely overexpressed in epithelial cancers and highly associated with tumor growth, invasion, and metastasis^299^; (3) Peptides: For example, peptide GE11 also has the ability to target EGFR. Yu et al. reported that GE11-modified nano-photosensitizer showed internalization of the complex and enhanced toxicity in EGFR-positive cells, while no internalization was observed in EGFR low-expressed cells^300^; (4) Other targeting strategies include: vitamins,^301^ steroid-targets,^302^ and carbohydrates,^303^ etc.

### Bioorthogonal click reactions

Beyond the passive and active targeting effects, biorthogonal click reactions also be utilized to improve the targeting of tumors for nanomedicines. Click chemistry refers to a set of chemical reactions with high yield, fast reaction rates, and non-toxic byproducts, which can achieve artificial chemical conjugation on the cell surface and cytoplasm.^304^ Therefore, its research is currently widely applied in targeted imaging, drug delivery, and other fields. Its main mechanism is: that artificial chemical groups are introduced into tumor cell surfaces through metabolic glycoengineering firstly, with the most common being bioorthogonal azide groups, which are used for click chemistry with subsequently injected nanoparticles, enabling the nanoparticles to target tumor cells without any biological targeting moieties.^305^ For example, Hong et al. reported that the active targeting of tumors by FA ligand-mediated nano-PTs significantly improved the tumor targeting of the subsequently injected rose Bengal PSs through bioorthogonal click reaction, thereby significantly improving the inhibitory effect of the two PSs on tumors.^306^

### Summary

The advent of the nanotechnology era has provided multiple effective solutions to address the deficiencies of traditional PTs. Different types of nanomaterials, as well as different shapes and sizes of nanoparticles, possess unique optical and electrical properties. The highly tunable nature of nanophotonic materials significantly enhances their ROS generation efficiency. The characteristic of easy surface functional modification of nanomaterials significantly improves the physical properties of PTs, such as water solubility. The unique aggregation-induced emission effect of AIEgens addresses the low efficiency of traditional PTs due to aggregation quenching. Additionally, the volume and high surface area-to-volume ratio of nanomaterials makes them perfect drug carriers that can be combined with traditional PTs to achieve synergistic antitumor effects. The strengths and limitations of different PSs are summarized in Table 1.

**Table 1:** A comprehensive overview of the advantages and disadvantages associated with various generations of phototherapeutic agents

Categories	PTs	Strengths	Limitations
Nobel metal NPs	Au nanorods	1). High tunability 2). The optical properties depend on the shapes, sizes, and atomic quantities. 3). High surface area 4). Good biocompatibility 5). Stable under irradiation 6). Facile surface modification 7). High extinction coefficient	1). The long-term toxicity and systemic toxicity of nanomaterials require further investigation. 2). The penetration ability of nanomaterials within heterogeneous tumor tissues needs more exploration. 3). Nanosensitizers have yet to effectively address the reduced efficacy of PDT caused by the tumor’s hypoxic microenvironment and inadequate light penetration in deep tissues. 4). Composite nanomaterials with multiple strategies require complex synthesis processes, have low reproducibility rates, and cannot be mass-produced.
	Au nanoshells		
	Au nanoechinus		
	Au nanoclusters		
Semiconductor photocatalyst nanoparticles	TiO_2_	1). NIR light excitation, providing enhanced tissue penetration. 2). Good biocompatibility. 3). Flexible optical properties	
	ZnO		
	BiVO_4_		
	g-C_3_N_4_		
Carbon-based nanomaterials	Carbon nanotubes	1). Generation of ROS even in hypoxic TME. 2). Ease of surface functionalization. 2). Good biocompatibility. 3). High stability and low photobleaching.	
	Graphene		
	Oxidized graphene		
	Fullerene		
2D nanomaterials	2D-LDH	1). Enhanced optical and electrical properties. 2). Large surface area-to-volume ratio. 3). Good biocompatibility. 4). Tunable bandgap with layers independence	
	2D-TMDCs		
	TMOs		
	MXene		
	2D-MOF		
	BP		
	SACs		
AIEgens	CdSe	Addressing the low ROS production caused by the aggregation quenching of traditional PSs.	
	CdTe		
QD	SQDs	1). Strong light absorption capacity and high ROS generation ratio. 2). Broad excitation spectra, narrow emission spectra, and large Stokes shifts. 3). Size and composition tunable emission. 4). Ease of surface modification. 5). Great molar extinction coefficients. 6). Good stability	
	CQDs		
	CND		
	GDQs		
Cu-Cy	**Cu-Cy**	1). can be excited by X-rays, ultrasound, microwaves. 2). can undergo Fenton-like reactions with H_2_O_2_ to produce ROS.	

*Au* aurum, *TiO*_*2*_ titanium dioxide, *ZnO* zinc oxide, *BiVO*_*4*_ bismuth vanadium oxide, *g-C*_*3*_*N*_*4*_ graphitic carbon nitride, *2D* two-dimensional, *LDH* layered double hydroxides, *TMDCs* transition metal dichalcogenides, *TMOs* transition metal oxides, *MXene* transition metal carbides, nitrides, and carbonitrides, *MOF* metal-organic framework, *BP* black phosphorus, *SACs* single-atom catalysts, *AIEgens* aggregation-induced emission agents, *CdSe* cadmium selenide, *CdTe* cadmium telluride, *QD* quantum dots, *SQDs* semiconductor-based dots, *CQDs* carbon quantum dots, *CND* carbon nanodots, *GDQs* graphene quantum dots, *Cu-Cy* Cu-cysteamine, *ROS* reactive oxygen species, *TME* tumor microenvironments, *H*_*2*_*O*_*2*_ hydrogen peroxide

With the rapid advancement of nanotechnology and increasing nanoparticle exposure, concerns about their biological impact have escalated. Nanoparticles can traverse biological barriers, accumulate in various organs, and cause damage, raising significant toxicity issues. Current research on nanomedicine toxicity remains limited, highlighting a need for a deeper understanding of nanomaterials’ potential health risks.^307^ Current knowledge indicates that the toxicity of NPs is influenced by particle size, shape, morphology, composition, surface area, and surface chemistry. From a pharmacokinetic perspective, the absorption, distribution, metabolism, and excretion processes of therapeutic nanoparticles in the body are all related to their toxicity. Oral NPs may face absorption challenges, accumulating in the intestines and potentially harming the gut. Intravenously administered NPs can aggregate in the bloodstream, interact with serum proteins, and may cause capillary blockages and thrombosis.^308^ The administration route, size, and surface properties of nanomedicines can also influence the biodistribution of nanoparticles, thereby affecting their toxicity. Nanoparticles with sizes between 5 and 200 nm are less likely to be quickly cleared and tend to have prolonged circulation in the bloodstream. They may also extravasate through the fenestrations of liver endothelial cells.^309^ Moreover, an increasing number of reports suggest the ability of nanomaterials to penetrate the placental membrane, and intrauterine exposure can lead to fetal toxicity.^310^ Engineered nanomaterials can cross the blood-brain barrier to enter the central nervous system, and their neurotoxicity strongly depends on the material’s properties.^311^ Nanomedicines are primarily metabolized in the liver, and inorganic nanoparticles exhibit high accumulation in the liver, which may be associated with their chronic hepatotoxicity.^312^ Moreover, the acidic pH in cellular lysosomes may lead to the degradation of inorganic nanoparticles, releasing metal particles. The reaction of free metal ions with biomolecules can potentially induce toxicity.^313^ The size and charge of nanoparticles influence their excretion pathways, thereby affecting their toxicity. However, increasing efforts are being made to reduce or avoid the potential toxicity of nanomaterials. For example, positively charged nanoparticles can enhance interactions with intestinal mucus, reducing their retention.^314^ The active targeting technique using targeting ligands can reduce the distribution of nanoparticles in other tissues. Implementing a protective layer on the surface of nanomaterials can reduce their interaction with proteins in the bloodstream, minimizing recognition and clearance by the immune system, and so on. Future research efforts are still needed for more in-depth studies to reveal the toxicity of nanomaterials, providing a theoretical foundation for the better design of nanoparticles for human use.

## Overcoming the hypoxia limitation for PDT

Oxygen, one of the three main elements, plays a very crucial role during the antitumor process of PDT. However, the hypoxic environment within a tumor remains a significant obstacle to its widespread clinical application. Most solid tumors are hypoxic, primarily due to the imbalance between oxygen supply and consumption within the tumor, a consequence of rapid growth.^315^ In normal tissue, the oxygen level (*p*O_2_) typically ranges from 4 to 7.5%, while solid tumors exhibit values of 0.3–4.2%.^316^ The rapid growth of tumors results in incomplete, collapsed, and disordered tumor vascular systems, severely limiting tumor perfusion to inducing acute hypoxia. As tumors progress, O_2_ diffusion is further restricted by the increased distance from vessels, while inadequate oxygenation within tumor cells generates extensive CO and causes the so-called chronic hypoxia.^317^

Moreover, the hypoxic TME induces resistance to PDT through several mechanisms: (1) Oxygen is essential for generating ROS by most PSs. The hypoxic TME directly diminishes ROS generation during PDT. Additionally, oxygen consumption during PDT exacerbates hypoxia within tumors, establishing a detrimental cycle. (2) The hypoxia-activated hypoxia-inducible factor-1 (HIF-1) dimer upregulates VEGF and other angiogenesis factors to shield vascular endothelium from PDT-induced damage.^318^ (3) Tumor cells predominantly rely on glycolysis as the primary energy generation pathway due to hypoxia. Evidence suggests that certain glycolysis intermediates, such as pyruvate and lactate, can scavenge PDT-generated ROS by elevating the levels of the cellular redox system components (reduced GSH/oxidized glutathione(GSSG)).^319^ (4) Studies have demonstrated that tumor cells acquire various adaptive abilities in the hypoxic microenvironment, such as inducing HIF-1α to bind to the hypoxia response element in vacuole membrane protein 1 (VMP1) promoter, triggering VMP1-related autophagy, and thus reducing the cell death rate after PDT treatment.^320^ Hypoxia also activates the NF-кB signaling pathway to sustain cell survival by preventing apoptosis and promoting angiogenesis.^321^ (5) Lastly, hypoxia induces the formation of a tumor immune suppressive microenvironment through a series of steps, including upregulating the expression of chemotactic cytokines 22 (CCL22) and 28 (CCL28), accumulating myeloid-derived suppressor cells and regulatory T cells (Tregs),^322^ facilitating the conversion of macrophage and neutrophil into tumor-promoting phenotypes, and inhibiting T cell and NK cell activity.^323^

To date, strategies to alleviate tumor hypoxia can be mainly divided into two categories: increasing the oxygen content within the tumor and utilizing oxygen-independent PDT. Increasing the oxygen content within the tumor includes: (1) Exogenous delivery of oxygen. (2) Endogenous production of oxygen. (3) Normalizing tumor vasculature or stroma to improve oxygen transport and distribution within the tumor. (4) Inhibiting cellular respiration to reduce oxygen consumption. (5) Suppressing the HIF-1 signaling pathway. Oxygen-independent PDT primarily includes: (1) Fenton-reaction-like oxidative therapy. (2) Type-I PDT.

### Enhancement of oxygen content in tumors

#### Endogenous O_2_ generation in situ

The in situ generation of endogenous O_2_ stands out as the most direct and effective approach to counteracting hypoxia in tumors and alleviating tumor hypoxia. Compared to the delivery of exogenous O_2_ into tumor sites, endogenous O_2_ generation mitigates the risk of O_2_ leakage during transport.^324–326^ Common methods for achieving in situ endogenous O_2_ generation include: (1) Decomposition of H_2_O_2_. (2) Splitting of water.^327–332^

### Decomposition of H_2_O_2_ to O_2_

**Fig. 5:**
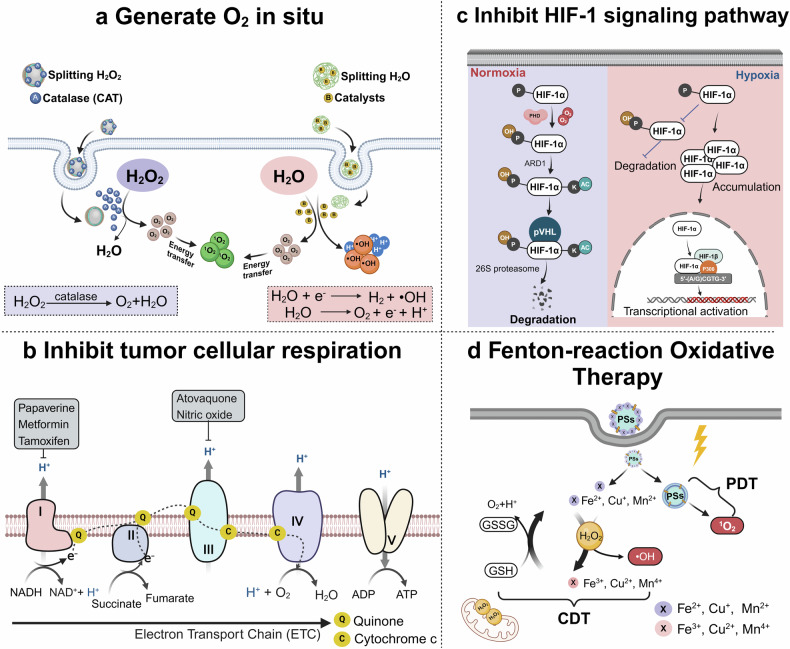
Overview of several strategies for overcoming hypoxia limitations. **a** Overview of Decomposition of H_2_O_2_ to O_2_ and Splitting water to O_2_ within the tumor. **b** Mechanisms of inhibition of tumor mitochondrial oxidative phosphorylation. **c** Overview of HIF-1 pathway under normal and hypoxia conditions in cellular. **d** Illustration of the chemical reactions and related biochemical effects in tumor cells induced by PDT + CDT. NADH nicotinamide adenine dinucleotide. NAD nicotinamide adenine dinucleotide. ADP adenosine diphosphate. ATP adenosine triphosphate. HIF-1 hypoxia-inducible factor-1α. PHD prolyl hydroxylase domain. pVHL von Hippel–Lindau. PDT photodynamic therapy. PS photosensitizers. CDT Chemodynamic therapy. GSH glutathione. GSSG oxidized glutathione. The figure was created with BioRender.com

The overexpression of H_2_O_2_ within tumor cells is widely acknowledged and may be associated with DNA damage, abnormal proliferation, and tumor metastasis.^36^ Catalase (CAT) is one of the fundamental oxidoreductases in the human body, and possesses the capability to decompose H_2_O_2_ into O_2_ and H_2_O (seen in Eq. 5). Therefore, researchers have proposed using CAT in high H_2_O_2_ concentration tumors to generate O_2_ in situ. This approach enhances the production of ROS during PDT (Fig. 5a).

5 $$2{H}_{2}{O}_{2}\to 2{H}_{2}O\left(l\right)+{O}_{2}\left(g\right)$$

Zhou et al. developed a CatCry-MB platform using CAT crystals as a scaffold for PS, methylene blue (MB), enhancing prolonged H_2_O_2_ breakdown. Its nanoporous structure efficiently confined O_2_ and MB, optimizing O_2_’s utilization for MB. The platform showed superior phototoxicity compared to free MB and Cat-MB under hypoxic conditions, with reduced in vivo HIF-1α fluorescence in CatCry-MB-treated subjects, indicating effective hypoxia alleviation.^333^ Huang et al. reported the development of ultra-pH-sensitive polymer complex nanomicelles composed of CAT and albumin. The incorporation of albumin mitigated the immunogenicity of CAT, thereby reducing its crosslinking with immune cells. The ultra-pH-sensitive polymer enabled effective evasion of lysosomal degradation, ultimately ensuring efficient delivery of CAT to the tumor site. Experimental evidence demonstrated that in breast cancer, nanomicelles loaded with a photosensitizer effectively accumulated and penetrated throughout the tumor, generating sufficient O_2_ to reverse hypoxia and enhance the efficacy of PDT.^334^

Natural CAT is limited by its high cost, instability, and sensitivity to environmental conditions. The emergence of CAT-like nanozymes can address these issues. These nanozymes combine CAT’s catalytic functions with nanomaterial properties, offering versatile applications in therapy, drug delivery, and detection. Various types of nanomaterials have been identified to exhibit CAT-like activity, falling into three categories: (1) Inorganic nanomaterials: metal, metal oxides, MOF, etc. (2) Organic nanomaterials: carbon-based, aryl boronic ester-based,^325^ and sixth main group element-based.^335^ (3) CAT-based biomaterials: CAT-based liposomal materials^50^ and CAT-integrated hyaluronic acid.^336^ Different nanozymes operate through distinct catalytic mechanisms, broadly classified into two major mechanisms: (1) Heterogeneous cleave catalysis, which preferentially breaks the H–O bond in H_2_O_2_. (2) Homolytic catalysis that preferentially breaks the O–O bond.^337^ Taking CeO_2_, a widely used metal oxide nanomaterial, as an example, it has demonstrated high intrinsic catalase activity, catalyzing the production of H_2_O and O_2_ from H_2_O_2_ through heterogeneous cleave catalysis. The catalytic reaction can be summarized by Eqs. 6–11.^338^

6 $${H}_{2}{O}_{2}\left(g\right)\to {H}_{2}{O}_{2}\,*$$

7 $${H}_{2}{O}_{2}* \to H* +{HOO}\,*$$

8 $$H* +{HOO}\,* \to {H}^{+}+{H}^{+}+{OO}\,*$$

9 $${H}^{+}+{H}^{+}+{OO}\,* \to {H}^{+}+{H}^{+}+{O}_{2}\left(g\right)$$

10 $${H}_{2}{O}_{2}* +2{H}^{+}\to {H}_{2}O* +{H}_{2}O\,*$$

11 $${H}_{2}O* +{H}_{2}O\,* \to {H}_{2}O\left(g\right)$$

Chen et al. developed a CeO_2_-based core-shell nanoplatform with enzymatic activity and high photothermal efficiency, loaded with the photosensitizer ICG. Their research demonstrated in vitro and in vivo oxygen generation, significantly reducing tumor hypoxia and achieving effective tumor ablation under 808 nm laser exposure.^339^ Furthermore, several metal-based nanozymes, such as Mn (Eq. 12)^340^ and Pt (Eq. 13)^341^ based nanozymes, possess CAT-like catalytic capabilities (Eqs. 12–13).

12 $${{MnO}}_{2}+{H}_{2}{O}_{2}+2{H}^{+}\to {{Mn}}^{2+}+{O}_{2}+2{H}_{2}O$$

13 $$2{H}_{2}{O}_{2}\overline{\to }2{H}_{2}O+{O}_{2}$$

Chudal et al. reported the enhanced therapeutic efficacy of PpIX for breast cancer treatment using a liposome bilayer structure (PpIX-Lipo-MnO2), leveraging MnO2’s reaction with H_2_O_2_. Under hypoxic conditions, PpIX-Lipo-MnO2 showed significant, concentration-dependent cytotoxicity against MCF-7 cells upon UV exposure, outperforming the PpIX-Lipo group in therapeutic effects.^342^

### Splitting H_2_O to generate O_2_

Water splitting, a key process, generally requires three primary elements: an external energy stimulus, a catalyst, and water. In line with the mechanism described in “Overcoming the phototherapeutic agents limitations” section semiconductor PTs, the absorption of a photon with energy exceeding the catalyst’s bandgap excites an electron from the CB to the VB, creating an electron-hole pair. These generated electrons and holes then separate, migrate to their respective end-points, and engage in redox reactions with the surrounding water molecules.^343^ Specifically, the reduction of water by electrons (hydrogen evolution reaction) generates H_2_,^344^ while the oxidation of water by holes (oxygen evolution reaction) affords O_2_ and •OH^345^ (Fig. 5a).

Inorganic semiconductor materials with photocatalytic effects have been comprehensively discussed in Chapter 5; thus, they will not be reiterated here. However, it is worth mentioning that in addition to inorganic water-splitting materials, microorganisms capable of utilizing thylakoid membrane-bound chlorophyll molecules for oxygen-evolving photosynthesis (e.g., cyanobacteria) can also be harnessed to increase O_2_ levels in tumors.^346,347^ Currently, commonly used photosynthetic microorganisms include chlorella,^348^ cyanobacteria,^349^ and spirulina.^350^ Wang et al. devised a light-controlled durable PDT based on chlorella and perfluorocarbons (PFCs) to achieve the simultaneous production of O_2_ (by chlorella) upon irradiation and its collection and enrichment (by PFCs).^351^ PFCs have been demonstrated to be high-safety materials facilitating the extraction of O_2_ from water.^352^ Light exposure enables the photosynthetic microorganisms (chlorella) to produce oxygen and enhance the oxygen level around the PS to maintain continuous oxygen production. Additionally, chlorella was shown to activate dendritic cells and stimulate antitumor immunity. Overall, this sustainable PDT represents an advancement in current PDT and holds potential for treating advanced cancer patients in the future.

#### Exogenous O_2_ delivery into tumors

In addition to in situ O_2_ generation, delivering exogenous O_2_ into tumors shows great promise for overcoming hypoxia. Hyperbaric oxygen (HBO) therapy can independently increase O_2_ delivery, thus enhancing PDT efficacy through a “carrier-free” method.^353^ However, the applications of HBO are limited by its toxicity to the central nervous system and the pulmonary system.^354^ Therefore, the precise delivery of O_2_ to the tumor without causing systemic side effects has become a mainstream solution strategy. The carriers used mainly include: (1) Hemoglobin (Hb). (2) PFCs. (3) MOFs. (4) Micro-/nanomotors-based Carriers.^355–357^

### Hb-based carriers

Hb serves as a natural oxygen carrier in red blood cells (RBCs), responsible for binding and delivering O_2_ in the body.^358^ Hb can reversibly bind with four oxygen molecules to form HbO_2_. Utilizing the Bohr effect, Hb-carried oxygen can easily be released in tumor microenvironments rich in H^+^ and CO_2_.^359^ However, the auto-oxidation of free Hb during circulation reduces its oxygen-carrying capacity, leading to severe renal toxicity and cardiovascular complications.^360^ Given the challenges related to its low stability and short circulation half-life, free Hb is not an optimal candidate for delivering oxygen to tumors.^360,361^ Consequently, researchers typically construct Hb-based nanocarriers, such as liposomes, to overcome limitations. Luo et al.^358^ and Wang et al.^362^ used poly(lactide-*co*-glycolide) and fusogenic liposomes, respectively, to mimic Hb-carrying cell membranes. Beyond liposomes, various protein hybridization approaches have been developed to design Hb nanocarriers. Liu et al. developed an aggressive man-made RBC (AmmRBC) system^363^ containing Hb, enzyme-like PDA, PS (MB), and vesicles. In this system. In this system, which is highly compatible with the parent RBCs due to their identical membranes, PDA acts as an antioxidative enzyme to protect Hb from auto-oxidation during circulation.^364^ Additionally, the capability of PDA to engage in strong adhesion allows the accommodation of aromatic compounds such as PDT agents or antitumor drugs.^365,366^

### PFC-based carriers

Unlike Hb, which can only bind with four oxygen molecules, O_2_ has a higher solubility in PFCs, approximately 40–50 ml O_2_ per 100 ml liquid, equivalent to the solubility of 200 ml O_2_ under the conditions of 25 °C and 1 atm. Therefore, PFCs have been considered a crucial material for O_2_ delivery systems.^36^ PFCs do not form a chemical bond with O_2_, instead, they utilize their own weak intermolecular interactions to physically dissolve. As a result, the release of O_2_ does not require an allosteric factor. Therefore, the O_2_ dissolved in PFC can be fully utilized by tissues, with a utilization rate of up to 90%. The release of O_2_ from PFC mainly occurs through tension gradient diffusion. Additionally, PFC nanodroplets are numerous, small in volume, and easy to transport through capillaries to deliver oxygen.^367^ In hamster experiments, injection of 4.2 g/kg body weight of PFC NE increased oxygen supply to the whole body and microvasculature by 25%.^368^

Various PFC-based nanomaterials have been developed to eliminate tumor hypoxia, such as perfluorohexane,^369^ perfluorooctyl bromide,^370^ and perfluoro-15-crown-5-ether^371^ have been extensively used to design O_2_-transporting nanoformulations. In addition, studies found that specific stimulation can accelerate the release of O_2_ from PFC, for example, Song et al. developed an ultrasound-based system using PFC nanodroplets stabilized with human serum albumin.^372^ This system demonstrated that oxygen was absorbed in the lung, carried through the blood to the tumor, and efficiently released within the tumor. In the tumor, oxygen levels rapidly increased from 17 to 49%, enhancing the antitumor efficacy of the PDT protocol. However, researchers have also indicated that O_2_ in PFC is released through a tension gradient, this process is uncontrollable. To address this problem, Zhang et al. fabricated a controllable carrier-and-trigger “oxygen bomb” system, PFC/SiPc@PSt@PNIPAM-Au_980_-DOX (PSPP-Au_980_-D), by encapsulating a PFC core in a functionalized bilayer polymer shell and evaluated the ability of this system to enhance the performance of PDT, photothermal therapy (PTT), and chemotherapy.^373^ This system triggers the release of O_2_ and the chemotherapy drug DOX from PFC nanodroplets through photothermal effects under 980 nm excitation, followed by PDT effects excited under 680 nm laser irradiation. This stepwise treatment strategy enables oxygen to be released in a controlled and effective manner, providing a new approach for the treatment of hypoxic tumors.

### MOF-based carriers

Microporous solids have high internal surface area and pore volume, and they can concentrate gas molecules through adsorption at specific temperatures and pressures. The gas density in microporous solids is much higher than that dissolved in typical liquid solvents.^374^ Metal-organic frameworks (MOFs) are open porous crystalline structures with permanent porosity created through reticular synthesis using strong bonds by connecting metal-containing units (secondary building units) with organic linkers.^375^ The unique permanent porosity of MOFs makes them promising candidate materials for many industrial applications as an alternative to traditional adsorbents. Proper selection of metals and organic linkers can result in materials with open metal centers that can selectively capture oxygen through electron transfer chemistry. These framework structures containing coordinatively unsaturated redox-active metal centers with reversible binding and reduction of oxygen have stronger selectivity and total adsorption capacity than traditional adsorbents.^376^ In addition, MOFs can be further modified by synthesis and functionalization techniques, which allow for the incorporation of organic linkers or metal-organic complexes, and thus improve the oxygen adsorption efficiency, making them a promising gas carrier for cancer therapy. For example, a Zr-based MOF (UiO-66) with high surface area and tunable pore size has been utilized for the storage of a large amount of O_2_.^377^. Gao et al. utilized UiO-66 as an O_2_ carrier and grafted it with a commercial PS (ICG) to form UiO-66@ICG. Experimental results confirmed that the O_2_ adsorption capacity of UiO-66@ICG could reach 500 μmol/g, and the oxygen concentration in the O_2_@UiO-66@ICG solution rapidly increased under laser irradiation, indicating its effective oxygen release rate.^378^

### Micro-/nanomotors-based carriers

Micro-/nanomotors are a type of nanoparticles that achieve autonomous propulsion by converting various forms of energy (chemical or other external energy) into mechanical energy. In recent years, they have received attention in drug delivery, overcoming hypoxia, improving PDT, and other fields. The motion characteristics of these motors promote their penetration in blood vessels and tissues, while also facilitating the diffusion of carried oxygen and PS within the tumor, thereby improving the antitumor efficacy of PDT that is limited by hypoxia and heterogeneous PS distribution.^379^ These micro-/nanomotors can be classified into several types according to different driving mechanisms: (1) Catalytically powered motions. For example, catalyzing the generation of oxygen and water from H_2_O_2_, where the oxygen bubbles propel the motor to move,^380^ or utilizing enzymes to catalyze urea and glucose to provide power^381^; (2) Alternative fuels powered motions. For example, using bodily fluids (such as gastric acid) as fuel. When the nano-motor is immersed in a strongly acidic medium, a redox reaction occurs involving the generation of hydrogen gas, which is then utilized to propel the motor using hydrogen bubbles^382^; (3) Magnetic micro/nanomotors. These micromotors can move in various biofluids under an external magnetic field without requiring fuel. The non-harmful nature of magnetic fields to the human body makes magnetic micro/nanomotors suitable for in vivo applications^383^**;** (4) Ultrasound-powered micro/nanomotors. Similar to magnetic power, Garcia-Gradilla et al. have demonstrated the use of ultrasound-driven nanomotors based on nanoporous gold fragments to increase drug or gas payload.^384^

These motors could facilitate the penetration of oxygen and PSs into hypoxic areas as well as promote oxygen and PS diffusion within tumors.^385^ Gao et al. constructed an acoustically powered magnetically navigated red blood cell-mimicking (RBCM) micromotor capable of autonomously moving in whole blood by converting ultrasonic energy into movement energy.^386^ Specifically, the PS (ICG) was wrapped by a magnetic Hb core in the shape of a double-concave erythrocyte, and a natural erythrocyte membrane shell was attached to the motor. When exposed to an acoustic field, the motors were able to move in the biological medium at a speed of ~56.5 μm s^−1^. Thus, the RBCM micromotor provides an innovative, rapid, and biocompatible method of delivering active oxygen and PSs for future PDTs.

#### Modulating tumor vasculature and stroma

As previously mentioned, one of the mechanisms contributing to the formation of the hypoxic microenvironment in tumors is the irregular morphology of tumor vasculature, characterized by irregular shapes, high degrees of curvature, increased vascular permeability, and slow blood flow. The aberrant tumor vasculature inevitably leads to abnormal tumor perfusion, limiting the delivery of oxygen and therapeutic agents. Additionally, abnormal vascular structures, such as the detachment of pericytes surrounding endothelial cells and increased vascular permeability, can result in the leakage of proteins from the vessels and the accumulation of interstitial fluid within the tumor matrix. This, in turn, elevates tumor interstitial fluid pressure (TIFP) and hinders the penetration of oxygen and photosensitizers, posing significant challenges to cancer treatment.^387^ Elevated TIFP exacerbates the hypoxic state of tumors, creating a vicious cycle. Normalization of tumor vasculature can increase intratumoral blood perfusion, restore endothelial cell junctions to reduce metastasis and lower TIFP. This, in turn, enables more effective delivery of oxygen and therapeutic agents to tumor cells and has become one of the promising strategies to enhance the efficacy of PDT.^388^ Strategies employing drugs to normalize vasculature can be categorized into three main types: (1) Traditional antiangiogenic drugs, such as the multi-kinase inhibitor (Regorafenib),^389^ the multi-targeted tyrosine kinase inhibitor (Lenvatinib),^390^ and the EGFR inhibitor (Erlotinib).^391^ For example, Wan et al. created a conjugated polymer delivery system combining a NIR-II excitable photosensitizer with regorafenib. Upon irradiation with an 808 nm laser, regorafenib was released, significantly alleviating tumor hypoxia through vascular normalization and generating more ROS to eradicate the tumor.^389^ (2) Drugs capable of inhibiting angiogenesis include: ^1^O_2_, which activates the TRPV4-endothelial nitric oxide synthase (TRPV4-eNOS) signaling pathway,^392^ the glucocorticoid dexamethasone,^393^ and histidine-rich glycoprotein (HRG), which interacts with myosin.^394^ (3) Gas therapy, including CO, NO, and H_2_S, can reshape the TME by improving tumor vasculature and inducing vascular normalization.^395,396^ Wu et al. reported a lipid nanoparticle designed to target the TME that can specifically deliver and release H_2_S and a photosensitizer within tumors. Utilizing high concentrations of H_2_S, this approach reduces TIFP, promotes angiogenesis, increases vascular permeability, and ameliorates hypoxia to reprogram the TME. This significantly enhances the uptake and therapeutic efficacy of the photosensitizer.^397^

However, there are limitations to be addressed in alleviating tumor hypoxia through anti-angiogenesis. On one hand, one of the antitumor biological effects of PDT is to induce vascular occlusion and thrombosis, but such vascular occlusion also hinders the penetration and distribution of drugs and oxygen within the tumor. Therefore, the combined use of PDT and antiangiogenic drugs requires careful coordination to balance the pros and cons. On the other hand, the mechanisms of tumor angiogenesis and the action mechanisms of antiangiogenic drugs are not yet fully understood. More basic research is needed to explore the relationship between tumor vasculature and anti-angiogenesis during different stages of tumor progression.

#### Inhibition of tumor cellular respiration

Tumor cells predominantly rely on glycolysis to generate ATP, known as the “Warburg effect”,^398,399^ whereas normal cells depend on mitochondrial oxidative phosphorylation (OXPHOS). However, cancers exhibit heterogeneity in their respiration lines. The transcription of OXPHOS genes is downregulated in some tumor types but upregulated in others.^400^ In solid tumors, the expression of the OXPHOS gene also shows heterogeneity based on their vasculature. For example, cells located far from tumor vessels mainly rely on glycolysis for energy, while those close to tumor vessels mainly rely on OXPHOS.^401^ Current consensus suggests that both glycolysis and OXPHOS are crucial for providing the energy for macromolecule synthesis and cell proliferation in tumors.^402^ When O_2_ demand exceeds supply due to the increased cell respiration, tumor hypoxia ensues. Thus, inhibiting the OXPHOS pathway in cancer cells preserves oxygen for the PDT reaction^331^ (Fig. 5b). Several inhibitors of the mitochondrial respiratory chain have been explored, including atovaquone (ATO),^403–406^ papaverine (PPV),^407,408^ 3-bromopyruvate,^409,410^ tamoxifen,^411,412^ metformin (MET),^413–416^ and nitric oxide (NO).^417–420^ Among them, PPV, MET, and tamoxifen reduce the O_2_ consumption rate by inhibiting the electron-transport-chain complex I in mitochondria, whereas ATO and NO inhibit mitochondrial respiration by suppressing complex III.

For instance, Yang et al. designed a two-stage antitumor therapy based on the inhibition of mitochondrial respiration followed by an attack on mitochondria and tumor cells. A PS (IR780) and OXPHOS inhibitor (MET) were coloaded into an amphipathic nanocarrier, poly(ε-caprolactone)-poly (ethylene glycol) (PEG-PCL), to afford PEG-PCL-IR780-MET (P-P-I-M) nanoparticles.^413^ When the tumor was irradiated with an 808-nm laser for 1 min, the rapidly generated ROS disintegrated PEG-PCL to release MET and IR780. MET was proven to inhibit the mitochondrial electron-transport-chain complex I and thus effectively suppress cellular respiration. To verify that P-P-I-M can inhibit cellular hypoxia, the study used a hypoxia detection kit to confirm the presence of hypoxia in tumor cells, and then subsequently found that tumor cells treated with P-P-I-M and activated showed no obvious hypoxia red fluorescence, but produced significant ROS green fluorescence. In addition, in vitro and in vivo experiments confirmed that P-P-I-M had a significant inhibitory effect on tumors.

#### Inhibition of the HIF-1 signaling pathway

HIF is a heterodimer consisting of an O_2_-sensitive α-subunit (HIF-α) and a shared β-subunit (HIF-β).^421^ Under normoxic conditions, HIF-1α undergoes rapid degradation through interaction with the von Hippel–Lindau (VHL) protein, a process regulated by the prolyl hydroxylase domain (PHD) protein and factor-inhibiting HIF (FIH).^422^ As O_2_ is required for PHD and FIH activities, hypoxic conditions inhibit the hydroxylation of HIF-1α, enabling it to avoid degradation, translocate to the nucleus, and be dimerized with HIF-1β.^326,423^ This dimerization activates downstream genes involved in glucose metabolism, cell proliferation, migration, and angiogenesis (Fig. 5c).^315,422,424,425^ Therefore, inhibiting the HIF-1 signaling pathway is a potential strategy for reducing tumor hypoxia and enhancing PDT efficacy.

Zhang et al., for instance, utilized curcumin (Cur), a natural bioactive compound with significant antitumor activity, to design and construct NIR-triggered core-satellite upconverting nanoparticles (Cur-CSNPs).^426^ Cur can significantly downregulate HIF-1α levels and generate ROS upon laser irradiation.^427–429^ In Cur-CSNPs, Cur was encapsulated in satellite-like upconverting nanoparticles (UCNPs) linked by a ROS-responsive thioketal (TK)-containing PEG unit. PDT-generated ROS promoted the rapid release of Cur from the nanoparticles to inhibit HIF-1α activation through the breakage of the TK-containing PEG unit. Experimental demonstrated that the expression of HIF-1α was significantly inhibited in treated 4T1 cells with a dose-dependent relationship.

While antiangiogenic drugs directly target the VEGF to reduce the tumor vascular supply, which may exacerbate hypoxic TME, Ang-II receptor blockers were shown to decrease the expression of VEGF, reduce abnormal vessel density, increase vessel wall thickness, and relieve hypoxia.^430–433^ Chen et al. designed an integrated system (RSCDs) containing candesartan (CD), an Ang-II receptor (AT_1_R) blocker, for delivering HIF-1α siRNA to achieve synergistic effects on tumor reconstruction.^434^ After assembly with HIF-1α siRNA, RSCDs were coated with modified hyaluronic acid (HA-SS-COOH) to afford HA-RSCD/siRNA. HA-RSCD/siRNA reduced HIF-1α expression in vitro and in vivo, alleviating hypoxia-induced tumor sensitivity and inhibiting vascular growth by 60%. Furthermore, the combination of HA-RSCD/siRNA with Ce6 liposomes resulted in the best inhibition effect (inhibition efficiency = 63%) significantly superior to that observed for Ce6-liposome-only treatment (inhibition efficiency = 26%).

### Oxygen-independent PDT

#### Fenton-reaction-like oxidative therapy

Chemodynamic therapy (CDT) is an oxidation therapy that has garnered significant attention due to its ability to generate ROS via Fenton-type and Fenton-like reactions.^435,436^ Specifically, CDT employs transition metal catalysts (e.g., Fe, Mn, Cu, Ni, and Co) to convert H_2_O_2_ into cytotoxic •OH, thereby causing substantial oxidative damage to tumor cells.^437–442^ Unlike O_2_, H_2_O_2_ is highly abundantly present in the TMEs, making CDT unaffected by hypoxia.^443,444^ Hence, it holds promise as a strategy to enhance the antitumor efficacy of PDT. The classical Fenton reaction utilizes iron-based catalysts to generate ∙OH through the following equation (equations of 10–12) (Fig. 5d):

14 $${{Fe}}^{2+}+{H}_{2}{O}_{2}\to {{Fe}}^{3+}+\,\cdot\, {OH}+{{OH}}^{-}$$

15 $${{Fe}}^{2+}+\,\cdot\, {OH}\to {{Fe}}^{2+}+\,\cdot\, {OOH}+{H}^{+}$$

16 $${{Fe}}^{3+}+\,\cdot\, {OOH}\to {{Fe}}^{2+}+{O}_{2}+{H}^{+}$$

Unlike PDT, which induces apoptosis, Fe-based CDT treatment inflicts iron-dependent LPO-associated oxidative damage that induces regulated cell death, which can also be denoted as ferroptosis.^96,445–447^ However, the clinical translation of this approach faces limitations due to the insufficient generation of •OH and the low rate of Fe-based Fenton reactions.^448^ Cu-based Fenton-like reactions have a greater CDT potential than Fe-based Fenton reactions because of the adaptability to weakly acidic TMEs, high rate of •OH generation, and greater rate of the former. The redox properties of Cu are strikingly similar to those of Fe, e.g., both Cu^+^ and Cu^2+^ easily react with H_2_O_2_^277^(seen in equations 13 and 14):

17 $${{Cu}}^{2+}+{H}_{2}{O}_{2}\to {{Cu}}^{+}+{{HO}}_{2}^{\cdot }+{{OH}}^{-}$$

18 $${{Cu}}^{+}+{H}_{2}{O}_{2}\to {{Cu}}^{2+}+\,\cdot\, {OH}+{{OH}}^{-}$$

The Cu^2+^/H_2_O_2_ Fenton-like system is applicable over a broader pH range than the Fe^3+^/H_2_O_2_ system in view of the higher solubility.^449^ In addition, Cu^2+^ complexes are more easily decomposed by •OH than Fe^3+^ complexes, which precludes the deactivation of Fenton reactions.^450^ Furthermore, Cu^2+^ can be reduced by GSH to increase the concentration of redox-active species (Cu^+^) used to generate •OH (Cu^2+^ + GSH → Cu^+^ + GSSG).^451^ Such characteristics highlight the favorable properties of the Cu^2+^/H_2_O_2_ Fenton-like system for enhancing the efficacy of antitumor therapies. Combining PDT and CDT can achieve high ROS production under low oxygen conditions (Fig. 5b). The nano-photosensitizer Cu-Cy reported by the Wei Chen team has been proven to be a special material that can be excited by UV light to generate ^1^O_2_ through Type-II reaction and can also undergo a Fenton-like reaction with H_2_O_2_ to produce •OH.^278^ Li et al. achieved Cu^2+^-mediated protein self-assembly (named C-m-Abs) by integrating copper with a photosensitizer (ICG). It can effectively consume intracellular GSH, and degrade H_2_O_2_ to generate O_2_, thereby counteracting hypoxic conditions and enhancing ICG-induced PDT effects.^452^ The study observed accelerated O_2_ production and significant •OH production when C-m-Abs were mixed with H_2_O_2_.

#### Type-I PDT

PSs can be converted from the ground state to the singlet state via two routes: Type-I and Type-II. Compared to the commonly used Type-II PDT, Type-I PDT is less affected by oxygen and can be activated under hypoxic conditions. Therefore, oxygen-independent Type-I PDT provides a novel approach for treating hypoxic tumors. The free radicals generated during the Type-I process instantaneously interact with H_2_O or O_2_ to produce H_2_O_2_, O_2_^•−^, and •OH.^453^ Since Type-I PDT was first defined in 1991, researchers have developed various Type-I PSs, which can be categorized into nanomaterials and small-molecule materials.^454^ Nanomaterial-based Type-I PSs include metal oxides (e.g., TiO_2_) capable of electron reactions,^455–458^ carbon-based nanomaterials (e.g., carbon dots or C_3_N_4_),^459–461^ organic-inorganic hybrid nanomaterials (e.g., MOFs) formed by the covalent linking of organic and inorganic fragments with unique physicochemical properties,^462,463^ supramolecular nanomaterials,^464^ and Z-scheme heterostructured nanomaterial.^465^ TiO_2_ and ZnO exhibit particularly excellent Type-I ROS generation capabilities, showing promising prospects in Type-I PDT.

Supramolecular materials are typically formed by non-covalent forces, including hydrophobic-hydrophobic interactions, π–π interactions, electrostatic interactions, and van der Waals forces.^466^ The weak interactions of these non-covalent forces make supramolecular materials often prone to reorganization and disassembly, making them responsive to internal stimuli (such as pH, enzymes, redox agents) or external stimuli (such as temperature, light, etc.) with rapid reactions, for example, the generation of ROS.^467^ Another type of nanomaterial-based Type-I PS is composed of a Z-scheme heterostructured nanomaterial with narrow bandgaps, typically consisting of two photochemical reaction systems composed of two types of semiconductors. Although semiconductors have proven effective in photocatalytic conversion, their actual performance can be unsatisfactory because a relatively narrow bandgap is needed for the separation of electrons and holes. However, efficient ROS generation requires a more negative CB potential and a more positive VB potential (i.e., a wider bandgap). This contradiction makes it difficult for a single photocatalyst to simultaneously meet the requirements of efficient light utilization and powerful ROS generation.^468^ Therefore, Z-scheme heterostructured nanomaterials consisting of two semiconductor photocatalytic systems can facilitate the separation of holes generated in system II and electrons generated in system I, thereby simultaneously producing O_2_ and ROS, and exhibiting superior performance. For example, Bi_2_S_3_@Bi nanorods designed by Zhang et al. Utilizing Z-scheme heterostructured design to generate ROS under NIR laser irradiation. In this system, Bi_2_S_3_ can utilize the holes in its VB to react with water, producing O_2_, while Bi can drive the electrons in its CB to react with O_2_, producing O2^•−^.^469^ Ji et al. developed a Z-scheme heterostructured 2D pyrite nanosheet (TOPY-PEG NSs) with FeS_2_ as the core and Fe_2_O_3_ as the shell. In this structure, the photoexcited electrons in the CB of Fe_2_O_3_ can transfer to the VB of FeS_2_, thereby delaying the recombination of electron-hole pairs generated in the FeS_2_, allowing the holes in the FeS_2_ CB and the electrons in the Fe_2_O_3_ VB to exhibit stronger redox potentials for the generation of O2^•−^ and •OH.^465^

Small-molecule Type-I PSs can be classified into organic molecules (such as MB and its derivatives^470,471^), which have better biocompatibility and fewer side effects, and transition metal complexes (e.g., those based on Ru(II),^472^ Ir(III),^473^ Zn(II),^474^ and Pd(II)^475^) which possess favorable photo-/physicochemical properties and promote electron transfer and ROS generation. An exceptionally efficient Type-I PS with substantial vascular disruption ability was developed by Chen et al. for tumor-specific PDT in hypoxic and metastatic conditions.^476^ In particular, a bifunctional organic nanoconjugate (named BDPVDA) nanoplatform was coated with self-assembled mPEG-PPDA (PBV NPs). After irradiation, the PBV NPs generated large amounts of O_2_^**•**−^ through efficient core-shell electron transfer. Despite severe hypoxia (2% O_2_), PBV NPs exhibited superior killing power in vitro. In contrast, almost no O_2_^**•**−^ was observed in the group treated with DBV NPs without the mPEG-PPDA coating. Significant cell apoptosis was observed in the PBV + laser group in the study, whereas no apparent apoptosis was observed without irradiation even at a high PBV concentration. Meanwhile, in vivo experiments showed that treatment with PBV+ laser achieved the greatest tumor inhibition compared to control groups. Similarly, Li et al. developed a molecule, COi6-4Cl, supporting the D-A configuration of a π-conjugated small molecule with hypoxia-tolerant PDT Type-I and -II combinations.^477^ COi6-4Cl is a rare, efficient phototheranostic material exhibiting excellent photosensitizing properties after excitation with light through Type-I and Type-II PDT processes. COi6-4Cl NPs can undergo Type-II PDT with O_2_ in addition to undergoing Type-I PDT without O_2_, killing 95 and 90% of cells under normoxic and hypoxic conditions, respectively. Although hypoxia had no adverse effects on the PDT results, severe cell damage was observed under hypoxic conditions.

### Summary

In this chapter, we have summarized the strategies for overcoming the limitations posed by hypoxia in PDT and categorized them into two main approaches: enhancing oxygen content in tumors and employing oxygen-independent PDT modalities. Each strategy has its pros and cons, as outlined in Table 2.

**Table 2:** Overview of strategies for alleviating tumor hypoxia

Strategy		Strengths	Shortcomings
**Enhancement of the oxygen in the tumor**			
Endogenous O_2_ generation in situs			
	Decomposition of H_2_O_2_ to O_2_	1). Preventing premature oxygen leakage during circulation; 2). Tumors overexpress the substrate of H_2_O_2_ for CAT and metal nanozyme, making this method tumor-specific.	1). Efficiency limited by endogenous H_2_O_2_ concentration. 2). Biosafety assessments of metal ions are needed. 3). The ECM and abnormal tumor vasculature make alleviating hypoxia difficult in deep tumor tissue.
	O_2_ production via water splitting	1). The human body contains a large amount of water for producing oxygen; 2). Through water splitting, O_2_ can be continuously produced in cells, allowing chemical drugs to be avoided.	1). Harmful tumor microenvironment that harms the activity of organisms; 2). There is a safety concern due to the presence of bacteria. 3). The micrometer size limits deep tumor penetration; 4). Visible light wavelengths.
Exogenous O_2_ delivery into tumor			
	Hb-Based	1). Hb has reversible O_2_ binding ability and releases oxygen specific to tumors; 2). Hb could be easily released from tumors rich in H^+^ and CO_2_ because of the Bohr effect.	1). Low oxygen loading capacity; 2). Due to its auto-oxidation during circulation, free Hb may cause severe side effects.
	PFC-Based	1). PFC has excellent O_2_ affinity; 2). MRI can utilize PFCs for 19 F imaging; 3). FDA-approved materials such as perfluorohexane, the half-life of ^1^O_2_ is longer than in water and cells.	1). Due to premature oxygen release and thermal instability, tumor-specific oxygen release is relatively weak.
	MOF-Based	1). Multifunctional MOFs are capable of decomposition under acidic TME conditions.	1). Potential toxicity arisen from metal ions.
	Micro-/nanomotors-based carriers	1). Hypoxia may be improved with these motors by promoting oxygen and photosensitizer penetration and diffusion within tumors.	1). Maximum efficiency is limited by oxygen concentration.
Mitochondria inhibition		1). Reduce the cell’s O_2_ consumption; 2). Through inhibition of hypoxia-related signaling pathways (oxidative phosphorylation), to prevent the production of adenosine triphosphate, to inhibit tumor growth.	1). Limited efficacy of respiration inhibitors; 2). The drug resistance of tumor cells may harm therapeutic efficacy.
Inhibiting HIF-1 signal pathway		1). It reconstructs TMEs by reprogramming hypoxia metabolism, decreasing angiogenesis, combating tumor progression, and inhibiting tumor metastasis and migration.	1). The mechanisms for the switch from HIF-1α- to HIF-2α (3α)-dependent transcription need further study. 2). There is a challenge to design HIF-1 inhibitors (such as novel metal-based NPs to heighten the activity of PHDs or ubiquitin ligases for HIF-1α degradation) rationally.
**Oxygen-independent PDT**			
Type-I PDT		1). Type-I PDT is less affected by oxygen and could be triggered under hypoxia 2). Generation of ROS (H_2_O_2_, O_2_- and •OH) with stronger oxidation performance than ^1^O_2_.	1). It is still unclear how type-I mechanisms work, especially how oxygen is involved; 2). The issue of drug-resistant expression associated with hypoxia remains unresolved.
PAGs		1). Photoacid therapy is an oxygen-independent PDT strategy that directly targets the cytosol of cells by a PAG. 2). 1-PA is cheaper and easier, while 2-PA can penetrate tissues with incredible depth.	1). Because of 2-PA’s low penetration probability, 1-PA and 2-PA mostly need to combine.
Fractional PDT therapy		1). The tumor cells can be destroyed simultaneously by traditional PDT methods under conditions of light and by ^1^O_2_ during the period without light in this method of treatment.	1). The amount of ^1^O_2_ production may be restricted to the rapidly diminishing concentration of photosensitizer in tumor cells.
Fenton-reaction-like oxidative therapy		1). CDT utilizes Fenton or Fenton-like reactions, with transition metals as catalysts, to convert H_2_O_2_ to •OH to destroy tumor cells. In contrast with the O_2_, H_2_O_2_ is highly abundant in TME, so CDT is not affected by hypoxia;	1). There are still some concerns about the biocompatibility, tumor-targeting capacity of current studies; 2). The therapeutic performance was still far from satisfactory.
		2). In addition to triggering by the endogenous chemical energy, CDT can modulate the hypoxia and immunosuppressive TME.	

*CAT* catalase, *ECM* extracellular matrix, *TME* tumor microenvironment, *NPs* nanoparticles, *PHD* prolyl hydroxylase domain, *HIF* hypoxia-inducible factor-1α, *PDT* photodynamic therapy, *PA* photon absorption, *CDT* chemodynamic therapy, *Hb* hemoglobin, *PFC* perfluorocarbon, *MOF* metal-organic framework, *PAGs* photoacid generators

The strategy of decomposing H_2_O_2_ and H_2_O to generate in situ O_2_ within tumors leverages the high levels of H_2_O_2_ and the abundance of water in the TME. It also potentially mitigates premature O_2_ leakage during transfers. However, its efficiency is significantly constrained by the endogenous levels of H_2_O_2_ and water, as well as the limited penetration capability of nanomaterials within tumors. These may lead to the heterogeneity of PDT efficacy. The Utilization of Hb, PFC, MOF, and micro-/nanomotors to deliver exogenous O_2_ into tumors has also been demonstrated to enhance the intra-tumoral oxygen content. MOFs, in particular, can decompose in the acidic TME, releasing O_2_ and to some extent reducing O_2_ consumption during transport. The robust tissue penetration capability of micro-/nanomotors can enhance the diffusion of O_2_ and PS within the tumor. Nevertheless, these materials face challenges regarding their actual oxygen-carrying efficiency, oxygen release and consumption during delivery, and potential toxicity, all of which require further optimization.

Inhibiting mitochondrial respiration in tumor cells not only reduces intracellular oxygen consumption but also decreases ATP production, thereby restricting tumor growth. However, the effectiveness of these drugs and the drug resistance urgently need to be addressed. By inhibiting the HIF-1 pathway, it is possible to reprogram the hypoxic metabolism of tumors, reduce tumor angiogenesis, and thereby inhibit their growth and metastasis. The mechanisms of this strategy and appropriate HIF-1 inhibitors still require further in-depth research. For the oxygen-independent PDT applications, Type-I PDT and CDT all utilize different principles to generate cytotoxic ROS without the need for O_2_. However, the efficacy of these treatments is currently unclear, and the biocompatibility of the required materials still needs further discussion.

## Overcoming the light penetration limitations

Light penetration depth is a critical factor influencing phototherapy, including both PDT and PTT. When light penetrates through human tissues, it gets absorbed by various biological molecules (e.g. proteins, lipids, DNA, and RNA), leading to varying degrees of light loss in the delivered to the tumor tissue.^164^ The inadequate penetration of light, a crucial prerequisite for phototherapeutic agents to exert phototherapy effects, has consistently been a significant challenge hindering the progress of phototherapy. Different wavelengths of light have different tissue penetration depths.^478^ For instance, as the wavelength increases from UV (150–380 nm), blue (390–470 nm), green (475–545 nm), yellow (545–600 nm), red (600–650 nm) to near-infrared (NIR, 650–1300), the tissue penetration depth increases from less than 0.1 mm to 3 mm in order.^479^ Most traditional PTs typically exhibit a favorable response to shorter wavelength, higher-energy photons, posing a challenge where light may not be sufficient to excite PTs deep within the tumor.

**Fig. 6:**
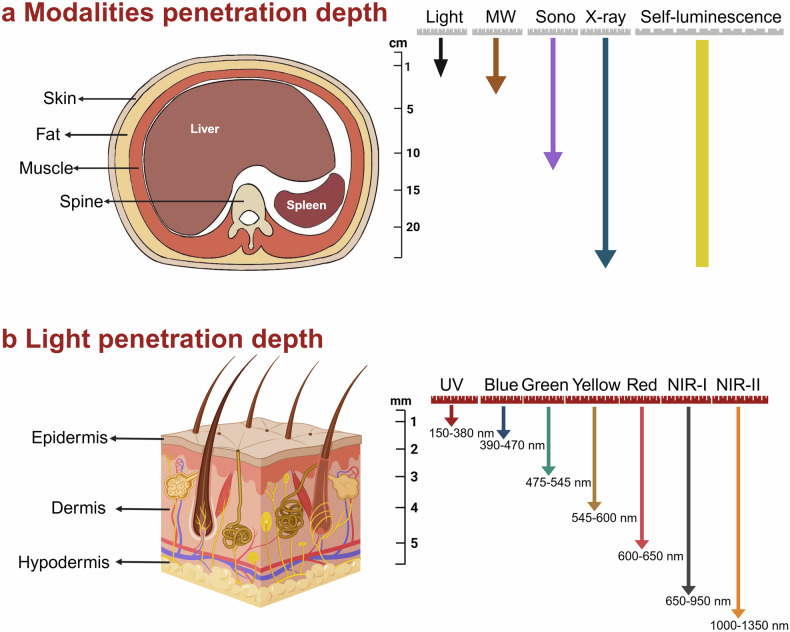
Penetration Comparison. Comparison of the penetration depth of various excitation modalities (**a**) and light penetration depth (**b**). NIR-1 Near-Infrared-I. NIR-II Near-Infrared-II. MW microwave. The figure was created with BioRender.com

The insufficient penetration of light, resulting in poor phototherapeutic outcomes for deep-seated tumors, remains a critical challenge in all current phototherapy approaches. To address this significant limitation, researchers have explored various innovative strategies, which are discussed in this chapter: (1) Using self-luminescent nanomaterials: These materials eliminate the necessity for external light sources. (2) Using of nanoscale PTs excited by NIR light: NIR light offers superior tissue penetration compared to UV and visible light. (3) Non-photoinduced phototherapy strategies: This includes PDT induced by X-ray, ultrasound (sono-induced), and microwave. (4) Implantable wireless systems: These systems can provide localized phototherapy without relying on external light sources, further overcoming light penetration barriers and improving the precision and efficacy of treatment (Fig. 6).

### Spontaneous luminescent nanomaterials for PDT

In contrast to the traditional PSs that rely on external light sources for excitation, some nanomaterials can emit light themselves and serve as internal light sources to activate photosensitizers for PDT. These nanomaterials primarily include chemiluminescence, bioluminescence, and Cherenkov radiation.

#### Chemiluminescent nanomaterials (CL)

CL refers to the phenomenon where certain substances produce light emission through chemical reactions. Specifically, the reactants or intermediate products can be oxidized to form an unstable high-energy intermediate (i.e., excited state). The decomposition of the intermediate releases energy by emitting photons, returning the substance to its ground state, thereby producing chemiluminescence. This method is also called direct chemiluminescence.^480^ Most CL molecules emit light through this mechanism, such as luminol and acridine esters. In contrast, during indirect chemiluminescence, the intermediate does not dissipate energy by emitting photons. Instead, it transfers energy through chemiluminescence resonance energy transfer (CRET) to excite nearby fluorophores or fluorescent molecules, causing them to emit light.^481^ Researchers have constructed CL-PDT systems utilizing the indirect chemiluminescence mechanism. By covalently modifying or non-covalently assembling CL with PS, ROS can be generated by exciting the surrounding PS through CRET when CL is activated by oxidation. This approach achieves the PDT effect without relying on external light irradiation.^482^ Covalent CL-PDT systems exhibit good stability and clear PDT effects due to the chemical bond connection, showing considerable potential in tumor imaging and treatments. Luminol is the most commonly used CL molecule in constructing covalent systems. Various PSs can be combined with luminol to form different nanoplatforms. For example, Zhang’s team designed a lumino-Ce6-PEG nanoplatform, which uses luminol to transfer energy to Ce6 under hydrogen peroxide conditions, thereby generating in situ ^1^O_2_ to kill tumor cells.^483^ However, the primary challenge with covalent CL-PDT systems is their complex synthesis and lack of flexibility. The non-covalent CL-PDT system, formed by the supramolecular self-assembly of CL, PS, and amphiphilic materials, allows for flexible mixing between CL and PSs. Functional molecules can be added as needed to maximize the PDT effect as well.^482^ In recent years, researchers have developed various non-covalent systems coupling different PS with luminol. For instance, Teng et al. used the co-assembly ability of polystyrene and c RGD-polystyrene to construct a metalloporphyrin-polymer dots (FeDP-Pdots) catalyst for the L012 (luminol analog)-H_2_O_2_ CL system. They successfully demonstrated that this system had a tumor inhibition rate of 79% for HeLa cells.^484^

#### Bioluminescent nanomaterials (BL)

BL refers to the phenomenon of light emission that occurs through the enzymatic catalysis of a chemical reaction. This phenomenon is widely observed in bacteria, dinoflagellates, insects, and worms.^485^ In simple terms, the process of BL involves the oxidation of a substrate (i.e. luciferin) by an enzyme (luciferase), resulting in the formation of an excited state. Subsequently, the excited luciferin returns to its ground state, emitting a photon of light at the same time.^486^ Similar to CL, the intermediate of BL can also dissipate energy through bioluminescence resonance energy transfer (BRET), which can excite nearby PSs to generate ^1^O_2_, thereby exhibiting the effect of PDT.^487^ Consequently, BL is widely used in research such as bioimaging and tumor treatment.

Different luciferin-luciferase pairs exhibit distinct luminescent properties. Currently, BL systems used in biological research mainly include the system with D-Luciferin as the substrate (e.g. firefly luciferase-luciferin, Fluc) and the system with coelenterazine as the substrate (e.g. Renilla luciferase-coelenterazine, Rluc). D-Luciferin needs to be activated by ATP before being oxidized to its excited state, differentiating it from coelenterazine. This makes D-Luciferin more stable in solution and less prone to auto-oxidation. Additionally, D-Luciferin exhibits strong water solubility,^488^ low toxicity,^489^ high quantum yield,^490^ and a long emission wavelength (λmax = 558 nm).^491^ However, studies have shown that the performance of the firefly BL system in mediating PDT is not satisfactory. Schipper et al. demonstrated that the firefly BL system is not sufficient to generate enough photons to excite rose Bengal and hypericin in vitro,^492^ and more efforts are needed to promote its application in antitumor PDT. For the Renilla-coelenterazine BL system, Lai et al. coupled the Rluc enzyme with carboxylate-containing quantum dots. The energy was transferred from coelenterazine to the quantum dots via BRET, and the quantum dots excited the PS through a FRET process, thereby exerting PDT effects. This study demonstrated that the Rluc-Qd-Ce6 system not only significantly delayed the growth of primary tumors, but also significantly reduced distant lung metastasis, prolonged the survival time of mice, and demonstrated the strong clinical potential of the BL-QD-PS system.^493^

#### Cerenkov radiation (CR)

CR refers to the phenomenon where charged particles (such as β^+^ or β^−^ particles) moving through a medium at a speed greater than the speed of light in that medium, causing the polarization of the molecules which emit photons during relaxation, and then resulting in the emission of blue light.^494^ When these molecules transfer energy to nearby PS during the relaxation process, PS activates and produces ROS, which is also known as Cerenkov resonance energy transfer (CRET).^495^ The β particles emitted by commonly used radioactive isotopes (such as 64Cu, 18F, 68Ga, 89Zr) in Positron Emission Tomography (PET) often have energies in tissues higher than the Cherenkov threshold, making them an ideal source of CR. The endpoint energy of these β particles in tissues determines their proportion. Nalinikanth Kotagiri et al. tethered the ligand of the TF receptor and a photo-generator based on Tc peroxide to the TiO_2_, which significantly delayed tumor growth and formed necrotic regions inside the tumor in the presence of ^64^Cu or ^18^F.^496^ However, CR is susceptible to attenuation by tissue, therefore, further improvement in CR luminescence is needed for CR-mediated PDT research. To address this attenuation, Wooseung Lee et al. constructed a ^64^Cu-labeled scintillator diethylenetriaminepentaacetic acid (DTPA) chelated Eu^3+^ (Eu-DTPA)/Victoria blue-BO (VBBO) loaded liposome (^64^Cu-Eu/VBBO lipo) nanoplatform and demonstrated that the luminescence efficiency of ^64^Cu-labeled Eu lipids in liposomes was 2 times higher than that of free ^64^Cu, and the energy transfer efficiency was 6 times that of CLET. In addition, significant inhibition of tumor growth was observed in vitro and in vivo experiments.^497^

### NIR light-excited phototherapy

As mentioned earlier, NIR light offers higher tissue penetration, less attenuation, low autofluorescence, and reduced phototoxicity to normal organisms,^498–500^ Thus, PTs with maximum absorption in the NIR window can be excited by NIR light for deep tumor phototherapy. Research over the past decade has predominantly focused on the NIR-I window; however, its maximum penetration depth is only approximately 1 cm, and it is characterized by high background interference and autofluorescence, resulting in a low signal-to-noise ratio. In contrast, the NIR-II window not only achieves tissue penetration depths of up to 3 cm in vivo and 12 cm in vitro but also exhibits minimal background interference and allows for a higher maximum permissible exposure of skin to light (>1.0 W cm^2^), hence offering more favorable optical properties.^501^ This is attributed to the reduction in light scattering as the wavelength increases; NIR-II exhibits lower scattering within tissues compared to NIR-I. The increased tissue penetration and reduced scattering enable the use of higher laser power for NIR-II, which enhances photothermal effects. Furthermore, the longer wavelengths mean each photon carries less energy, thus permitting higher maximum permissible exposure (MPE) power levels for irradiation without causing skin damage.^502^ The NIR-II nanomaterials reported to date include inorganic materials (noble metals, transition metal sulfides, transition metal oxides) and organic materials (small-molecule photosensitizers, semiconductor polymers, carbon-based materials, and quantum dots), some of which are detailed before and will not be reiterated here. This section mainly introduces this strategy using upconversion as an example.

#### Upconversion nanoparticles (UCNPs)

**Fig. 7:**
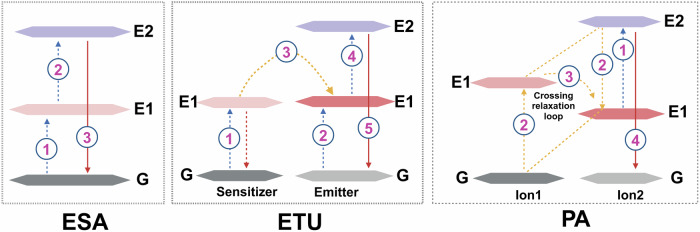
Illustration of the three main mechanisms of UCNPs. The blue, yellow, and red arrows represent the excitation, energy transfer, and emission processes, respectively. ESA, excited-state absorption. ETU, energy transfer upconversion. PA, Photon avalanche. The figure was created with BioRender.com

Another strategy for delivering light to deeper tumors involves using UCNPs that can be excited by NIR as energy donors. Photon upconversion refers to the anti-Stokes process where two or more low-energy photons are sequentially absorbed through real intermediate long-lived electronic states, which excite higher electronic states and emit higher-energy photons.^498^ Researchers categorized the working mechanisms of UCNPs into three core mechanisms: excited-state absorption (ESA), energy transfer upconversion (ETU), and photon avalanche (PA) **(**Fig. 7). Specifically, ESA refers to the process where a single ion absorbs one or more photons sequentially after excitation, transitioning from the ground state (G) to an intermediate energy state 1 (E1). Subsequently, before falling back to G from E1, it absorbs another photon and transitions to an energy state 2 (E2) and then emits a higher-energy photon while relaxing from E2 to G. Therefore, the gaps between G-E1, E1-E2, and the lifetime of E1 state are the critical factors affecting ESA upconversion.^503^ ETU is considered the most efficient mechanism in UCNPs and requires two adjacent ions (usually different ions), one sensitizer (ion 1), and one emitter (ion 2). Ion 1 absorbs a photon and transfers the energy to ion 2 by populating the energy E1, then ion 2 transitions to E2 and emits a high-energy photon through a nonradiative energy transfer method, while ion 1 relaxes to G. The efficiency of ETU is mainly related to the average distance between the sensitizer and the emitter, which is mainly affected by the concentration of doped ions.^504^ Photon avalanche (PA) is not a common upconversion mechanism. When the excitation energy is greater than a certain threshold, the ion jumps to the E2 state through the ESA process. Then, a cross-relaxation procedure occurs between ion 1 (E2) and ion 2 (G) (seen in Eq. 15):

19 $${Ion}\,1\left(E2\right)+{Ion}\,1\left(G\right)\to {Ion}\,1\left(E1\right)+{Ion}\,2\,\left(E1\right)$$

This loop creates two ions in the E1 state, and these two ions can be pumped to the E2 state to further drive the loop, similar to an avalanche process. Therefore, the key to the PA process is the need for a time-length excitation threshold.^498^

So far, research on UCNPs mainly includes several types: lanthanide-doped, transition metal-doped, defects-doped, etc., among which lanthanide-doped UCNPs are currently a hot research topic. Researchers have used lanthanide-doped UCNP as the energy donor and combined them with PTs through silica encapsulation, non-covalent physical adsorption, or covalent bonding.^505^ Zhang et al. coated the surface of UCNPs with a layer of mesoporous silica and then encapsulated the photosensitizer ZnPc molecules in the silica through physical adsorption. It was experimentally verified that under 980 nm NIR light excitation, the nanoparticles continuously generated ^1^O_2_ to kill cancer cells.^506^ Non-covalent physical absorption is a simple synthesis method that uses amphiphilic polyethylene glycol (PEG) polymers to transfer hydrophobic UCNPs to an aqueous phase to obtain water-soluble PEGylated UCNPs, which have a layer of hydrophilic stearic acid in between. The hydrophobic PTs will be absorbed into the hydrophobic layer, thus synthesizing UNCP-PS nanoparticles.^507^ Covalent conjugation is the most stable synthesis method among the three methods. Zhang et al. covalently immobilized the photosensitizer PPa’ on an 808 nm excitable UCNP core-shell to achieve NIR light-triggered cascade reaction in deep tumors. The experiment showed that under 808 nm light excitation, the nanocomb induced cascade reactions to produce ROS, significantly inhibiting tumor growth. They also demonstrated that the covalently linked PPa molecules are not only stable but also retain their spectral properties.^508^ Despite the aforementioned advancements, the excitation wavelength for lanthanide-doped UCNPs predominantly remains confined to the NIR-I region (<1000 nm). Recently, Er^3+^ ions have been identified as novel NIR-II photoreactive sensitizers that can facilitate the excitation of rare earth ions such as Nd^3+^, Eu^3+^, Ho^3+^, Tm^3+^, Tb^3+^ at 1532 nm, thereby extending the excitation window from NIR-I to NIR-II. For example, Bi and colleagues successfully utilized Er^3+^ as a sensitizer to construct Er/Mn co-doped NIR-II responsive UCNPs. The red upconversion luminescence intensity of the Er/Mn co-doped UCNP was enhanced by approximately eightfold, and, when loaded with ZnPc photosensitizers, enabled the PDT of tumor cells at 1532 nm.^509^

### Non-photoinduced dynamic therapy

In recent years, several non-photoinduced ROS generation strategies have been developed, including X-ray-induced (radiodynamic therapy, RDT), ultrasound-induced (sonodynamic therapy, SDT), and microwave-induced (microwavedynamic therapy, MDT) therapies. These dynamic therapies do not require external light irradiation, and their excitation sources, whether X-ray, ultrasound, or microwave, have better tissue penetration than NIR. The energy focused on deep tumor cells and the sensitizer made by these therapies can significantly improve the therapeutic effect of deep-seated tumors.^510^

#### Radiodynamic therapy (RDT)

Although NIR-mediated PDT for deep-seated tumors is feasible, the tissue penetration capability of NIR is still limited to less than 1.5 cm.^35^ X-ray has unlimited penetration capability, which makes it an ideal excitation source for deep-seated tumors. However, none of the traditional photosensitizers can be directly activated by X-rays. Therefore, researchers discovered a class of nanoparticles with high X-ray shielding ability, known as scintillating nanoparticles (ScNPs), which can convert X-rays to UV-Vis fluorescence. By careful assembly, efficient FRET can be achieved between ScNPs and PS, with PS generating ROS upon excitation by X-rays as the source of activation, thus exerting PDT effects. This therapy is also known as X-PDT firstly proposed and demonstrated by Wei Chen.^511–513^ On the other hand, the combination of X-rays and PDT can also lead to radiotherapy effects (currently also known as radiodynamic therapy, RDT) by directly transferring energy in the nanomaterial to produce O_2_^•−^ or •OH, without activating the photosensitizer.^514^ As a widely used cancer treatment modality in clinics, radiotherapy (RT) damages biological structures by directly damaging DNA with X-ray/γ photons or generating O_2_^•−^ or •OH through a reaction with H_2_O_2_ to kill tumor cells which is similar to PDT.^515^ However, the clinical application of RT is still limited to the high dose requirements (45–60 Gy).^516^ Therefore, the X-PDT approach can not only solve the problem of insufficient efficacy of traditional PDT but also enhance the efficacy of traditional RT at relatively low doses of X-rays.

The scintillation process can be divided into three steps: (1) conversion of incoming radiation into electron-hole pairs^517^; (2) transferring the energy of the electron-hole pairs to luminescent ions (such as PS) to excite them to higher energy levels^517^; and (3) relaxation of the luminescent ions from the excited state to the ground state, resulting in the emission of photons.^518^ Currently, the nanoparticles exhibit X-ray-induced luminescence mainly include the rare earth element-based nanoparticles (such as BaFBr:Eu^2+^, BaFBr:Mn,^2+^^[,519^ and LaF3:Tb,^3+^^[,513^ etc.), metal-based nanoparticles (such as ZnO NPs,^520^ copper-cysteamine, etc.), and non-metal-based nanoparticles (such as SiC/SiOx,^521^ etc.). Research has shown that the aforementioned scintillating nanoparticles (ScNPs) can activate PSs and generate a large amount of ROS under X-ray excitation, thus delaying tumor growth. Among them, Cu-Cy particles are a type of ScNP that possess inherent sensitization properties, and their rate of producing ^1^O_2_ is higher than any known X-ray-induced PS. They can still cause significant damage to cancer cells even under 2 Gy irradiation.^522,523^

#### Sonodynamic Therapy (SDT)

Compared to light, ultrasound has higher tissue penetration capability (>10 cm) and is widely used in clinical diagnosis.^524^ Therefore, SDT can affect lesions in deeper tissues. Additionally, SDT can control radiation frequency, time, and intensity, and accurately target deep tumor tissues, thus reducing damage to surrounding normal tissues.^525^ Studies have shown that SDT mainly treats tumors through the ultrasound cavitation effect. The energy produced by ultrasound changes the mechanical pressure of the liquid medium, which generates microbubbles in the tissue fluid and forms cavitation (which can be divided into non-inertial cavitation and inertial cavitation).^526^ When the ultrasound intensity is relatively low, the microbubbles in the tissue fluid do not undergo violent collapse but remain in a stable state and undergo small radius oscillations, which affect the surrounding cells and molecules to increase the cell permeability. This process is known as non-inertial cavitation, which can significantly increase the concentration of drugs within tumor cells.^527^ When the ultrasound intensity is high enough, microbubbles absorb a large amount of energy, undergo violent oscillation, expansion, and collapse, generating high temperature and pressure, and then releasing a large amount of energy. This process is also known as inertial cavitation.^527^ The release of this energy can activate nearby sensitizers from the ground state to the excited state, generating ROS such as ^1^O_2_ through type I and type II reactions. In addition, the energy generated by microbubble rupture can also directly cause the cleavage of surrounding water molecules, producing ROS that can kill tumor cells.^528,529^ Cavitation effect can induce not only typical type I and II reactions but also sonoluminescence and sonoporation. Sonoluminescence is a process in which sensitizers are excited through energy transfer, generating electron-hole pairs and subsequently producing ROS.^530^

Unlike X-rays that require special nanoparticles for excitation, many traditional PSs can be activated by ultrasound, serving as both photosensitizers and sonosensitizers. This includes both inorganic sonosensitizers and organic sonosensitizers. The classical inorganic nano-sonosensitizer is TiO_2_ NPs, which as described in Chapter 5, can generate ROS by transferring electrons from the valence band to the conduction band when exposed to UV radiation. Research has shown that TiO_2_ NPs exhibit high stability under US irradiation. Xi Wang et al. prepared highly dispersed mesoporous TiO_2_ nanoparticles (MTNs) as sonosensitizers, which produced ROS under ultrasound irradiation and exerted antitumor effects. In vivo biocompatibility tests showed no significant toxicity after single and repeated administrations.^531^ Currently, the widely studied inorganic sonosensitizers are mainly represented by Ce6 (one of the second-generation PSs). Studies have shown that Ce6 can be activated by either photo or ultrasound to generate high levels of ^1^O_2_, which reduces the survival rates of various breast cancer cell lines, induces cell apoptosis,^532^ and inhibits tumor cell adhesion and migration.^533^ Sun et al. successfully integrated Ce6 onto protonated g-C_3_N_4_ nanosheets (PCN) that were tightly bound to electrospun polycaprolactone/gelatin (PG) scaffolds pre-coated with graphene oxide (GO), forming Ce6@PCN-GO-PG composite scaffolds. This composite was directly used for sono-photodynamic therapy, which was activated by 808 nm laser and 1 MHz ultrasound to significantly enhance ROS production, resulting in the killing of 95.8% of breast cancer cells.^534^

However, current sonosensitizers are limited by their low ROS production rates. With advances in nanoscience, researchers have subsequently discovered novel and highly efficient sonosensitizers. For example, the novel Cu-Cy PS mentioned in Chapter 5 has been shown to achieve SDT effects. Research indicates that when focused ultrasound is applied at 0.5 W cm^−^^2^ for 1 min, Cu-Cy can enhance ultrasound cavitational effects, and this enhancement shows a Cu-Cy concentration-dependent behavior. After ultrasound induction, Cu-Cy exhibits significant cytotoxicity against MCF-7, 4T1, and MDA-MB-231 cell lines, with a considerable amount of ROS generated. Similarly, in 4T1 tumor-bearing mice, ultrasound-induced Cu-Cy treatment leads to the generation of a substantial amount of ROS in the tumor tissue, resulting in significant inhibition of tumor growth, which is also concentration-dependent.^272^ Additionally, titanium hydride (TiH 1.924), characterized by the presence of Ti in oxidation states Ti^0^, T i^2+^, T i^3+^, and Ti^4+^, is readily activated by external stimuli such as light, ultrasound, and microwaves, and thus has been identified as a potential sonosensitizer. Gong and colleagues produced TiH 1.924 nanodots through liquid-phase exfoliation from powdered form, which has been proven to generate ROS under ultrasound exposure, demonstrating a highly effective sonosensitization effect. Furthermore, these TiH 1.924 nanodots, possessing strong NIR absorption capabilities, also serve as potent PTAs, facilitating synergistic photothermal and sonodynamic therapy.^535^

#### Microwavedynamic therapy (MDT)

Microwave radiation, which has a lower frequency and longer wavelength electromagnetic spectrum than infrared light, is currently used in microwave hyperthermia to destroy tumors. This non-surgical method increases the blood flow within the tumor, thereby enhancing the oxygen content and mitigating the limitations of a tumor’s hypoxic microenvironment on PDT efficacy. In contrast to UV light, microwaves exhibit stronger tissue penetration capabilities. Unlike other energy sources used for hyperthermia, microwaves can easily pass through all types of tissues and non-metallic materials. As a result, microwave-induced PDT has emerged as a novel therapeutic approach, also known as MIPDT, to enhance the effectiveness of PDT for deep-seated tumors.^270^^[,536^ Although compared to UV (with an energy of around 4.1 eV), microwaves energy is too low (10^−^^3^ eV) to cleave chemical bonds and induce the formation of free radicals,^537^ researchers have found that it is possible to generate ROS using microwaves as the sole energy source in appropriate materials. Wu et al. discovered that liquid metal (LM) eutectic gallium-indium (EGaIn) supernanoparticles could act as a microwave sensitizer to generate ROS under microwave excitation.^538^ They synthesized IL-LM-ZrO_2_ supernanoparticles by loading EGaIn alloy containing 75.5% Ga and 24.5% In and an ionic liquid (IL) onto mesoporous ZrO_2_ nanoparticles. Under microwave excitation, LM produced ROS and induced tumor cell apoptosis, while IL generated MW heating effect, thereby achieving synergistic microwave-induced PDT and microwave-induced PTT antitumor effects. Through the experiments, it was demonstrated that the ROS generated by IL-LM-ZrO_2_ under microwave irradiation was 3.7 times higher than that under no microwave and 2.1 times higher than that generated under NIR irradiation. The authors proposed a possible hypothesis for the microwave-induced ROS mechanism. They suggested that under microwave excitation, the solution and IL-LM-ZrO_2_ SNPs rapidly heat up at different rates, leading to the temperature of IL-LM-ZrO_2_ SNPs being higher than that of the solution, resulting in the generation of “hot spots” in the mesopores of the supernanoparticles. In these hot spots, IL-LM-ZrO_2_ SNPs utilize the energy from microwaves to transfer electrons from Ga to adsorbed water and oxygen, resulting in the production of ∙OH and∙O_2_ (as shown in formulas 16–18)

20 $${Ga}-3e={{Ga}}^{3+}$$

21 $${O}_{2}+e=\,\cdot\, {O}_{2}$$

22 $$.{O}_{2}+2{H}_{2}O+3e=4\,\cdot\, {OH}$$

Wei Chen’s team also developed a copper-cysteamine nanoparticle photosensitizer that can be continuously activated by microwave to produce ^1^O_2_ and has time-dependent activity. In vitro and in vivo experiments have demonstrated that cu-cy NPs have a significant tumor-suppressive effect on tumor cells and tumor-bearing mice under microwave irradiation. It has been shown that Cu-Cy NPs inhibit tumor growth by inducing effective ferroptosis. This provides a promising solution for the treatment of deep-seated tumors.^270,539^ Chu et al. reported the use of graphitic carbon nitride (g-C3N4) quantum dots, a well-known photocatalyst for MIPDT, capable of releasing ^1^O_2_ under low-power microwave irradiation.^540^ Experimental demonstrated that the g-C3N4 QD solution under microwave irradiation could continuously generate ^1^O_2_, leading to significant cytotoxicity against the UMR-106 cell line, and this effect was dependent on microwave power and irradiation time. It is noteworthy that in the case of the g-C3N4 QD solution, microwaves are mainly used for heating and ROS generation. As the quantum dots require a large amount of energy to produce ROS, the heating effect of microwaves on them is not as pronounced as in pure water. Therefore, the antitumor mechanism of g-C3N4 QD-mediated MIPDT primarily relies on ROS mediation. In another study, Chu et al. reported on the use of TiO2 nanoparticles for MIPDT in treating osteosarcoma.^541^ TiO_2_ is an efficient photocatalyst that can decompose water under UV irradiation.^542^ Additionally, it can generate ROS under UV excitation, which can be employed for tumor treatment.^543^ The researchers found that a 5 W microwave treatment with TiO_2_ resulted in significant cell death in the UMR-106 cell line. Flow cytometry experiments revealed that TiO_2_ could increase the apoptotic rate of microwave-treated cells from 11% to 30% and 60%, which is 9 times and 6 times higher than g-C3N4 and Cu-Cy, respectively. In vivo experiments using mice with osteosarcoma also demonstrated a significant delay in tumor growth with TiO_2_ treatment.

In addition, other microwave sensitizers have been studied, such as iron metal-organic frameworks^544^ and AIEgens,^259^ but research on MIPDT is still in its early stages, and more effective microwave sensitizers need to be further developed. Furthermore, although direct activation of PTAs via microwave stimulation to achieve PTT effects has not yet been identified, Guo and colleagues first demonstrated the induction of photothermal effects for cancer therapy using graphite carbon nanocages, which were activated by NIR lasers with the assistance of microwave radiation. This combination of microwave and NIR enhances the efficiency of phototherapy.^545^

### Wireless PDT

In addition to replacing external devices to improve tissue penetration, researchers have developed implantable micro-scale photon devices and wireless functional systems to place wireless optical devices directly inside deep tissues, known as wireless photonic phototherapy.^546^ This technology can not only achieve thorough excitation of PTs within deep tissue but also enable multiwavelength light emission to optimize the activation of the PTs. Additionally, it can utilize wireless systems to control the dose of light emitted. Akshaya Bansal et al. assembled a wireless light source device with a volume of about 15 mm and a weight of about 30 mg, using a 3D spiral coil for energy harvesting and a microprinted circuit board integrated with an RF rectifier and light-emitting diode (LED). In vitro experiments demonstrated the ability of the device to generate ROS with different PSs (Ce6, zinc phthalocyanine, and PpIX) under irradiation. The device was also successfully used to activate Ce6 and generate ROS when installed on the surface of the liver in an adult pig model.^546^

However, researchers found that the wireless power source driving the implanted LED in the body requires an energy supply device with a specific working range, which to some extent limits real-time treatment for patients. Implantable PDT devices with sustainable power sources will improve patient treatment compliance. The nanogenerator technology, which converts mechanical energy into electrical energy, provides a new approach for sustainable power sources. Zhuo Liu et al. have developed a wearable and implantable self-powered PDT system (s-PDT) consisting of an energy harvester unit based on a twinning structure piezoelectric nanogenerator (ts-PENG), a power management unit (PMU), m-LED, and PS. The ts-PENG converts the biological energy of joint movement into electrical energy, which is then controlled by the PMU to light up the LED in different modes to activate PS and generate ROS. Through experiments, the s-PDT system was shown to achieve a tumor inhibition rate of 87.46% under continuous irradiation for 12 h.^547^ Although the use of encapsulation technology has improved the biocompatibility of wireless devices, the challenges of replacing devices implanted in deep tissues and immunogenicity have limited the clinical application of this technology. To address this, researchers have proposed another solution to avoid risks such as the need to replace batteries and implant leaks that can cause biological damage, by implanting light-converting materials instead of fiber optics, batteries, and other devices. Daniel Boon Loong Teh et al. have developed, for the first time, a PEGDA-encapsulated UCNP implant that converts NIR into visible light. The UCNP implant is transcranially inserted into the brain, and then NIR light is aimed at the scalp to irradiate the UCNP, which emits visible light to activate the photosensitizer PpIX targeting brain tumors and exerting an antitumor effect.^548^

### Summary

The light penetration depth has always been a major challenge in clinical phototherapy for deep-seated tumors. This chapter systematically discusses the main research directions to address this limitation, including the use of self-luminescent nano-photosensitizers, UCNPs, non-photo excited sensitizers (X-ray, sono, and microwave), as well as the implanted wireless light sources or light-converting nanoparticles. These various technologies and strategies have been shown to make significant progress in overcoming the challenges of light penetration, and their antitumor efficacy has been validated through in vitro and in vivo experiments. However, each of them still has certain drawbacks that need improvement (summarized in Table 3).

**Table 3:** A comprehensive overview of the advantages and disadvantages of strategies aimed at overcoming light penetration challenges in deep tumor phototherapy

Strategies		Laser source	Strengths	Limitations
**Spontaneous luminescent nanomaterials**	**Chemiluminescent NP**	**Not required**	1). Not limited by external excitation sources. 2). Can induce a more prolonged therapeutic process.	1). Weak luminescence intensity, resulting in generally lower PDT efficiency upon excitation. 2). Inability to adjust the power and duration of the light source. 3). Potential damage to normal tissues. 4). Chemical luminescent materials such as luminol may cause damage to biological components like DNA.
	**Bioluminescent NP**	**Not required**		
	**Cerenkov luminescence-excited PDT**	**Not required**		
**NIR light-excited phototherapy**	**Upconversion NP**	**NIR light**	1). NIR has good tissue penetration depth. 2). High chemical stability. 3). Tunable surface chemistries.	1). The penetration limit of NIR does not exceed 1 cm. 2). UCNPs have relatively low quantum yields. 3). NIR irradiation may cause thermal damage. 4). UCNPs are typically non-degradable. 5). The FRET efficiency between UCNPs and PS needs to be further improved.
**Non-photo-induced dynamic therapy**	**RDT**	**x-ray**	1). X-rays have good tissue penetration capability. 2). Combining radiotherapy and PDT can further enhance the treatment efficacy.	1). Low light conversion efficiency and low ROS generation. 2). Repeated X-ray exposure can cause severe radiation damage to normal tissues. 3). Requires special materials. 4). Scintillators have potential cytotoxicity.
	**SDT**	**Ultrasound**	1). Ultrasound has deep tissue penetration (>10 cm). 2). Ultrasound is widely used in clinical settings.	1). Ultrasound parameters are closely related to treatment efficacy, but there is currently no unified standard for ultrasound parameters. 2). The toxicity and biocompatibility of sonosensitizers require further extensive research and confirmation.
	**MDT**	**Microwave**	Microwaves have stronger penetration capability.	1). Microwave energy is too low to effectively induce ROS production. 2). Research is still in its early stages and requires further study to confirm its effectiveness.
**Wireless PDT**		**Light**	1). Facilitating thorough excitation of photosensitizers in deep tissues. 2). Controllable wavelength and dosage of the excitation source.	1). Surgical procedures are required for implantation and removal, which may cause side effects. 2). Long-term biocompatibility needs further validation after implantation. 3). Implantation may induce thermal effects on surrounding normal tissues. 4). Adverse immune reactions in the body may occur.

*PDT* photodynamic therapy, *DNA* deoxyribonucleic acid, *ROS* reactive oxygen species, *UCNPs* upconversion nanoparticles, *FRET* Förster resonance energy transfer

The advantage of the spontaneous luminescence strategy lies in its ability to harness energy transfer to generate ROS on nearby PS, whether using chemical reactions, enzymatic reactions, or Cerenkov radiation. Spontaneous luminescence does not require external stimuli, allowing it to disregard limitations related to tissue depth and exert a powerful anticancer effect on deep tumors. However, it is currently acknowledged that spontaneous luminescence has limited intensity, leading to lower PDT efficiency. Additionally, the absence of external devices implies low controllability. As materials enter the body and undergo pharmacokinetic changes, researchers face challenges in controlling their distribution, the generating duration, and intensity, potentially increasing the risk of toxicity. UCNPs can be excited by NIR light due to their ability to use low-energy photons to excite higher electronic states and emit higher-energy photons. NIR light is currently recognized for its superior tissue penetration depth. Additionally, UCNPs exhibit excellent tunable surface chemistries. However, the complex synthesis and preparation process of UCNPs, along with their potential toxicity, still require further efforts for clinical applications. Microwaves, ultrasounds, and X-rays possess greater tissue penetration capabilities. Utilizing them as external stimuli to excite sensitizers for inducing non-photo-induced phototherapy can significantly alleviate the limitations of insufficient light penetration. These three devices currently play a crucial role in the safe and effective treatment of clinical cancers, and their induction of non-photo-induced phototherapy can also achieve synergistic therapeutic effects. But their efficiency in generating ROS and heat is generally low, and they require specific sensitizers instead of conventional PTs. Additionally, repeated use of X-rays poses the risk of radiation damage. The implantation of wireless devices allows direct illumination of deep tumor, but this novel technology requires surgical implantation or removal and can result in side effects such as pain. Even with highly biocompatible materials for encapsulation or coating, long-term implantation can still cause immunogenic damage to the body. Therefore, implantable wireless light sources need further optimization of materials in terms of size, durability, and biocompatibility.

## Overcoming the inadequate immune response limitations

While PDT and PTT theoretically possess antitumor immunogenic potential, clinical trials have seldom reported effective immune responses induced by traditional phototherapy. Many phototherapies only provide modest protection against tumor recurrence and metastasis without eliciting a systemic antitumor immune response, primarily due to the immunosuppressive TME.^549^ As discussed in Chapter 2, PDT/PTT can activate immune responses but also stimulate immunosuppression. Additionally, the high temperatures may damage surrounding normal tissues, further weakening immune responses. The presence of an immunosuppressive environment significantly hampers the immune-activating potential of standalone PDT/PTT treatments. Consequently, combining PDT/PTT with immunotherapy, termed PIT, has emerged as a novel antitumor strategy. Current design strategies for PIT include: (1) Designing more effective PTs that produce greater heat or ROS, thereby generating a stronger ICD effect to enhance immune responses; and (2) Integrating immunotherapy with phototherapy, involving adjuvants, monoclonal antibodies, cancer vaccines, immune checkpoint inhibitors, adoptive cell therapies, cytokines, and costimulatory receptor agonists.^550^

Previous sections discussed strategies to enhance the antitumor immune response mediated by PIT, focusing on improving the efficacy of PDT and PTT and integration with various immunotherapeutic regimens. These include modulation of immunosuppressive cell populations, immune adjuvants, immune checkpoint blockade, and adoptive cell therapy. While the former has been detailed in other chapters, this section will primarily discuss the advancements in the latter approaches.

### Modulation of immunosuppressive cell populations

Immunosuppressive cells, including Tregs, M2 polarized TAMs, and MDSCs, are pivotal in the formation of the tumor immunosuppressive microenvironment and resistance to tumor therapies. Tregs promote tumor growth by inhibiting the antitumor immune functions of effector T cells, NK cells, and DCs through various pathways: (1) Secreting IL-10, TGF-β, and IL-35, which suppress immune functions via IL-10-dependent pathways. (2) Directly killing effector T cells through granzymes and perforin. 3) Forming positive feedback loops with factors produced by MDSCs, promoting the expansion of each group and further strengthening the immunosuppressive environment.^551^ M2-TAMs facilitate tumor progression and metastasis by: (1) Secreting anti-inflammatory cytokines such as IL-10, expressing co-inhibitory markers like PD-L1, and releasing matrix metalloproteinases (MMPs) and vascular endothelial growth factor.^552^ (2) Promoting abnormal activation of myelopoiesis, leading to differentiation of myeloid progenitor cells into MDSCs.^553^ MDSCs suppress immune responses through various mechanisms: (1) Secreting suppressive and anti-inflammatory cytokines (IL-10, TGF-β, IL-6, IL-28). (2) Producing ROS, expressing inducible nitric oxide synthase (iNOS, also known as NOS2) and arginase 1 (ARG1). (3) Collaborating with Tregs and Th17 cells. (4) Expressing immune checkpoint inhibitors. (5) Depleting certain amino acids in the tumor microenvironment (such as L-Arg and L-Trp), which not only impedes the antitumor function of T cell populations but also contributes to immunosuppression by establishing an oxidative stress environment.^554^

Research indicates that in the presence of tumor-derived factors, such as TGF-β, MDSCs can differentiate into immunosuppressive TAMs, DCs, or TANs.^553^ The crosstalk between MDSCs and Tregs is characterized by a mutually reinforcing positive feedback loop. Specifically, MDSC-derived IL-10 and TGF-β promote the induction, proliferation, and activation of Tregs. In turn, TGF-β and IL-10 secreted by Tregs can enhance the secretion of these cytokines by MDSCs. TGF-β and IL-10 promote the expression of immunosuppressive receptors (such as PD-L1) and enzymes on MDSCs. Tregs facilitate the secretion of IL-35 through the PD-1/PD-L1 pathway, and the subsequently secreted IL-35 enhances the secretion of IL-10 by MDSCs.^555^ Overexpression of indoleamine 2,3-dioxygenase (IDO) or arginase 1 (ARG1) in MDSCs contributes to the expansion mechanisms of Treg cells within the tumor.^556^ Additionally, there exists a bidirectional crosstalk between MDSCs and TAMs, significantly enhancing the immunosuppressive TME. This crosstalk results in increased production of IL-10 by MDSCs, while reducing the secretion of tumor-antagonizing IL-12 by TAMs.^557^

These immunosuppressive cells counteract tumor immune responses in various ways, presenting significant obstacles to immunotherapy and other antitumor treatments, such as chemotherapy. Numerous studies have shown a negative correlation between the abundance of MDSCs and the efficacy of immunotherapy. Preclinical studies indicate that treatment with anti-PD-1 antibodies leads to the activation of the PD-L1-NLRP3 inflammasome signaling pathway in cancer cells, resulting in increased recruitment of PMN-MDSCs to the TME, thereby acting as a mechanism of acquired resistance. Furthermore, M-MDSCs are associated with both primary and acquired resistance to PD-1 antibody therapy in non-small cell lung cancer patients through the galectin-9-TIM3 axis. MDSC-derived insulin-like growth factor-1 (IGF1) can lead to the activation of the IGF1 receptor-mediated PI3K pathway in glioblastoma cells, acting as a resistance mechanism to antagonistic antibody therapy targeting the macrophage colony-stimulating factor-1 receptor (CSF1R) on tumor-infiltrating myeloid cells.^558,559^ Studies have shown that PD-1 expression on eTreg cells is an important mechanism for resistance to PD-(L)1 antibody therapy, with the immunosuppressive activity of PD-1^high^ eTreg cells enhanced after PD-1/PD-L1 blockade, thereby diminishing the efficacy of immunotherapy.^560^ The role of TAMs in tumor growth and drug resistance is controversial, with increased recruitment of immunosuppressive TAMs, promotion of tumor-supportive polarization, reduced cytotoxic T cell responses, and activation of anti-apoptotic programs in malignant cells all being mechanisms by which TAMs exhibit tumor-promoting activity post-chemotherapy and in chemoresistance. Additionally, activated M2 macrophages may mediate chemotherapy resistance through the secretion of growth factors and inhibition of cell death signaling pathways.^561^ The role of TAMs in tumor growth and drug resistance is controversial, with increased recruitment of immunosuppressive TAMs, promotion of tumor-supportive polarization, reduced cytotoxic T cell responses, and activation of anti-apoptotic programs in malignant cells all being mechanisms by which TAMs exhibit tumor-promoting activity post-chemotherapy and in chemoresistance. Additionally, activated M2 macrophages may mediate chemotherapy resistance through the secretion of growth factors and inhibition of cell death signaling pathways.

Therefore, strategies that combine the depletion of immunosuppressive cells within tumors with phototherapy can enhance the antitumor immune response induced by phototherapy while avoiding systemic toxicity. For example, Sun et al. utilized the characteristic constitutive expression of glucocorticoid-induced TNFR-related protein (GITR) on Tregs, successfully developed a PDT and PTT nanoplatform (**PDA-ICG@CAT-DTA-1**) by loading catalase (CAT) and an anti-GITR antibody (DTA-1) onto PDA-ICG photothermal-photosensitizer nanoparticles. This platform not only kills tumor cells through PDT and PTT effects but also targets and depletes Tregs via the anti-GITR antibody, thereby eliminating tumor immunosuppression, inducing T cell activation, and generating a durable antitumor immune response.^562^ Due to the plasticity of the M2-TAMs, reprogramming macrophages from the M2 to the M1 phenotype within tumors represents a promising strategy for cancer therapy. For instance, Wan et al. developed a NIR-II responsive degradable pseudo-conjugated polymer (PSP) based PDT system for delivering a vascular normalization agent (regorafenib) (**NP-PDT@Reg**). This nanodelivery system releases regorafenib under 808 nm laser irradiation, improving tumor hypoxia by normalizing tumor vasculature, thereby enhancing the PDT effect and generating more ROS for antitumor activity. Additionally, the capability of regorafenib to reprogram M2 phenotype TAMs into M1 phenotype TAMs amplifies the PDT-induced ICD and reverses the immunosuppressive tumor microenvironment.^389^ Furthermore, current research has developed a variety of therapeutic strategies targeting MDSCs, including targeted depletion, inhibition of functionality, and prevention of recruitment.^563^ For instance, Chen et al. utilized gemcitabine (GEM), which preferentially reduces the percentage of MDSCs without necessitating the selective blockade of the JAK/STAT3 pathway to eliminate other leukocytes. They loaded GEM onto a tumor-targeted, light-responsive nanoplatform (Apt/PDGs^s@pMOF). This platform induces ICD through PDT, while the concurrent delivery of GEM depletes MDSCs, further reversing the immunosuppressive environment and ultimately enhancing the antitumor immune response.^564^

### Immune checkpoint blockade

Cytotoxic T lymphocyte-associated antigen 4 (CTLA-4) translocates to the surface of T cells from the intracellular environment following T cell receptor engagement, acting as a co-inhibitory factor that hinders T cell proliferation and activation. Blocking the CTLA-4 checkpoint can restore the initiation and activation of T cells to attack cancer cells. Another significant immune checkpoint is PD-1, which is expressed as a co-inhibitory receptor on antigen-specific T cells. When PD-1 binds to its ligand, PD-L1, expressed on cancer cells within the TME, it transmits regulatory signals to effector T cells, leading to T cell exhaustion, sending anti-apoptotic signals to tumor cells, and promoting tumor survival, thereby exacerbating immune suppression.^565^ Indoleamine 2,3-dioxygenase 1 (IDO-1) is a cytosolic enzyme overexpressed in tumor cells and DCs, enhancing immune suppression by increasing the kynurenine to tryptophan ratio, inhibiting the antitumor capacity of NK cells, and promoting Treg infiltration.^566^

Based on these mechanisms, various antibodies and small-molecule inhibitors that block immune checkpoints have been developed to date. Researchers are also exploring strategies to enhance the systemic antitumor immune response induced by phototherapy in conjunction with immune checkpoint inhibitors (ICIs). For example, Qu et al. recently designed a novel, light-responsive nanoplatform targeting pancreatic ductal adenocarcinoma (PDAC) microenvironment using tumor-specific intermediate filament nanobodies (Nbs). This platform efficiently delivers semiconductor polymer nanoparticles to the PDAC tumor TME and locally produces abundant ROS for precise phototherapy. When combined with PD-1 blockade, this targeted PDT platform exhibits optimal antitumor performance.^567^

Huang et al. co-encapsulated an immunomodulator (TGF-β inhibitor) and a PS (IR780) within nanoliposomes (Nano-IR-SB@Lip). This formulation enhances the immunogenicity of the TME by inhibiting the TGF-β pathway, increasing cytotoxic T lymphocyte numbers, and reducing Treg cells. It facilitates concurrent local PTT and systemic immunotherapy. Upon combination with an anti-PD-1 monoclonal antibody, this approach achieves photothermal therapy-induced ICD and a dual relief of immune suppression through TGF-β inhibition and PD-1/PDL-1 blockade.^568^ Additionally, Yang et al. developed erythrocyte membrane-cloaked nanoparticles for the precise delivery of a NIR-II photothermal agent (IR1061) and an IDO-1 inhibitor (1-MT). Local hyperthermia induced by PTT enhances CD^8+^ cytotoxic T lymphocyte presence at the tumor site. Moreover, the inhibition of IDO-1 activity by 1-MT, coupled with in situ-generated nitric oxide, normalizes tumor vasculature, reprogramming the immunosuppressive TME into an immunostimulatory phenotype. This strategy has demonstrated significant therapeutic effects in both primary breast cancer and metastatic tumors.^569^

### Immunoadjuvant therapy

Immunoadjuvants are immune-stimulating molecules that trigger the activation of innate and adaptive immune responses, enhancing the body’s immune response to antigens. They primarily interact with Toll-like receptors (TLRs) on antigen-presenting cells (APCs), such as B cells, macrophages, and notably DCs.^570^ Immunoadjuvants can be categorized based on their mechanism of action into several types, including (1) TLR agonist-based immunoadjuvants. TLRs, a family of pattern recognition receptors, are expressed on DCs, NK cells, macrophages, and epithelial cells, and can recognize and bind to DAMPs and pathogen-associated molecular patterns (PAMPs), such as lipopolysaccharides and heat shock proteins. The binding of pathogens to TLR ligands initiates an inflammatory response-mediated adaptive immune response (also known as TLR signaling transduction).^571^ The primary functions of TLRs include stimulating the maturation of DCs, the delivery and uptake of antigens, and ultimately inducing the differentiation of CD^4+^ T cells (Th1, Th2, and Th17) while inhibiting the function of Treg cells. TLR agonists include those located on the plasma membrane such as TLR1, 2, 4, 5, 6, 10, and 11, as well as those expressed intracellularly such as TLR3, 7, 8, and 9.^572,573^ Of particular note, is the TLR9-based immunoadjuvant, immunostimulatory cytosine-phosphate-guanine (CpG) oligodeoxynucleotides (ODNs). These sequences specifically bind to TLR9 and play a crucial role in recognizing specific DNA sequences and initiating immune responses against pathogens. Upon activation, TLR9 triggers a cascade of signaling pathways that activate immune cells such as dendritic cells and B cells, producing an inflammatory response and promoting adaptive immune responses. This immunoadjuvant is currently among the most promising in the field.^574^ (2) Inorganic immunoadjuvants, such as alum. Alum activates caspase-1 through the NALP3 inflammasome, secreting IL-1β and activating the innate immune system.^575^ (3) Exosomes. Exosomes are cell-derived nanovesicles that serve as carriers for proteins and nucleic acids from the parent cell. Exosomes from different cellular sources have distinct properties. For instance, in certain cancers, exosomes derived from tumor cells or DCs can present antigens to activate the immune system. These derived exosomes express costimulatory molecules MHC I and II on their surfaces, enhancing the activation of T and NK cells as well as the production of CD^4+^ and CD^8+^ T cells. Exosomes from B cells can form complexes with MHC molecules to present antigens.^576^ Li et al. designed a precursor nanoparticle based on chondroitin sulfate for delivering the PS (Ce6) and retinoic acid (RA), aimed at disrupting the Golgi apparatus and blocking immune suppression to enhance PDT-mediated immune responses. Additionally, combining CpG oligodeoxynucleotides as an immunoadjuvant to promote DC maturation, this approach demonstrated excellent antitumor efficacy in a mouse model combining PDT and immunotherapy.^577^

## Synergetic therapy strategies

The integration of phototherapy with other therapeutic modalities, or the combination of phototherapy with additional treatments is a strategy that is currently receiving significant attention, as it allows for the full utilization of the advantages of each therapeutic approach while compensating for their individual limitations. Common synergetic modalities include PDT combined with PTT, phototherapy combined with chemotherapy, and phototherapy combined with radiotherapy. In addition, emerging strategies such as PDT combined with Magnetic Hyperthermia, PDT combined with ion-interference therapy (IIT), and PDT combined with photoacoustic therapy are continually being explored. Due to space limitations, this article will provide a brief introduction to a few of these combined treatment strategies.

### PDT + PTT

**Fig. 8:**
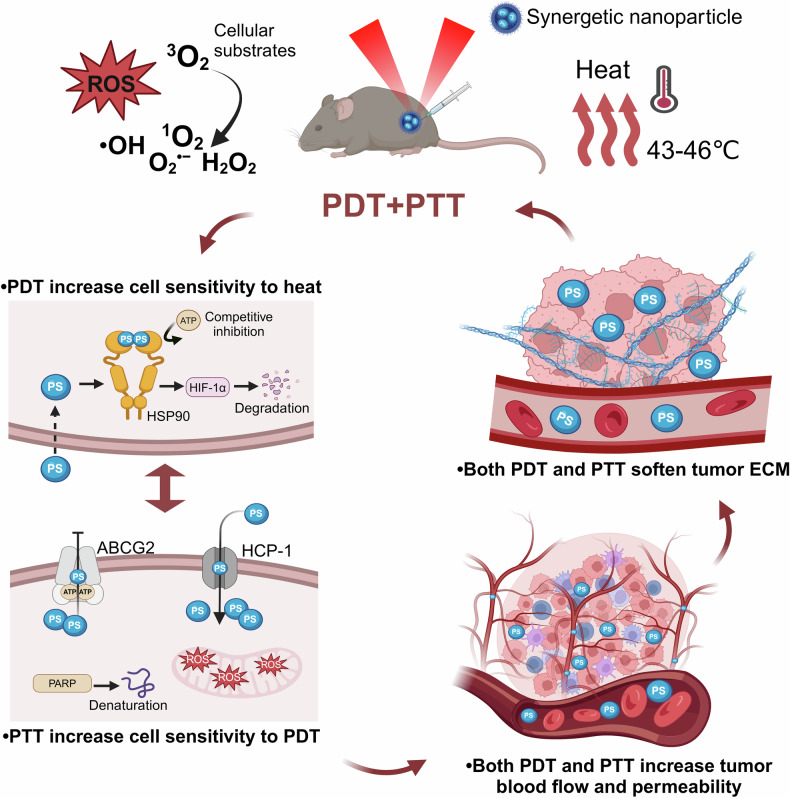
The mechanisms that combined PDT and PTT synergistic. PDT can enhance the sensitivity of cancer cells to heat, while the local hyperthermia induced by PTT increases the cells’ susceptibility to PDT. Both PDT and PTT can increase the permeability of tumor vasculature, improving oxygen saturation within the tumor tissue and enhancing drug distribution. Additionally, both therapies reduce the density of collagen in the ECM of tumors, softening the ECM and allowing for more effective drug penetration into the tumor. PDT photodynamic therapy. PTT photothermal therapy. ROS reactive oxygen species. ATP adenosine triphosphate. ABCG2 ATP-binding cassette subfamily G member 2. HCP-1 heme carrier protein 1. HSP90 heat shock proteins 90. HIF-1, hypoxia-inducible factor-1. PARP, poly-ADP-ribose polymerase. PS, photosensitizers. ECM extracellular matrix. The figure was created with BioRender.com

A substantial body of research indicates that the simultaneous or sequential use of PDT and PTT combined therapy can leverage their respective advantages to synergistically enhance anticancer efficacy.^578,579^ The mechanisms of synergy in the combined treatment can be elucidated by examining their effects on cells, vascular, and the extracellular matrix (ECM) (Fig. 8). A comprehensive review by Marta et al. thoroughly elucidated the mechanisms and applications of the synergistic treatment of PDT and PTT.^111^ In the mechanism of inducing cell death, when PDT depletes the tumor’s oxygen levels and induces tumor acidification due to the Warburg effect, the efficacy of PTT in killing tumor cells is enhanced.^580^ Additionally, PDT can enhance the sensitivity of cancer cells to heat. Tetrapyrrole-based PSs can interact with the ATP-binding pocket of HSP90, inhibiting its binding to other important intermediates (such as HIF-1a).^581^ PDT-generated ROS can directly attack HSP, negating its protective effect against heat damage.^582^ Conversely, the locally elevated temperature induced by PTT can increase cell sensitivity to PDT by denaturing proteins involved in DNA repair (such as poly(ADP-ribose)-polymerase-1, PRAP), increasing mitochondrial ROS levels, upregulating the expression of PS intracellular transport proteins (HCP-1), and downregulating the expression of PS efflux pumps (ABCG2), thereby enhancing PDT efficacy.^583^ In terms of the mechanism affecting blood vessels, mild heating from PTT can increase tumor blood flow, improve tumor tissue O_2_ saturation, and thus enhance the therapeutic effect of O_2_-dependent PDT.^584^ Moreover, both PDT and PTT can increase the permeability of tumor blood vessels, improve the distribution of nanoparticles within tumor tissue, and enhance treatment efficacy.^585^ In the impact on the tumor ECM, both PTT and PDT have been shown to reduce the density of collagen in the tumor ECM, soften the ECM, allowing drugs to penetrate the tumor more effectively, and can be utilized in combination therapy.^586,587^ Although PDT and PTT can each induce an anticancer immune response, the interaction between the two remains unclear and requires further in-depth research.

Research on combined PDT + PTT is typically designed in two ways: one involves using a dual-modal photothermal and photodynamic agent that can simultaneously exert PDT and PTT effects, and the other involves incorporating two photoactive agents within a single nanoparticle. The design rationale for the former usually utilizes the phenomenon of aggregation-induced fluorescence quenching and heat dissipation when assembling nanoparticles with a high concentration of monomeric photosensitizer. This allows the nanoparticles to function as a PTT reagent in the early stage. Once the nanoparticles rupture, the monomers disperse, and the photodynamic activity is restored, with the monomers then acting as PDT reagents. This design approach reduces the number of nanoparticles and synthetic steps, simplifying the treatment process. For example, the accumulation of a high concentration of porphysomes is used as an effective photothermal agent, and when porphyrin is destroyed, its photodynamic activity is restored.^588,589^ Additionally, HSA-ICG nanoparticles designed by Sheng et al., under NIR light irradiation, can simultaneously induce ROS and local hyperthermia (57.2 °C), achieving synergistic PDT + PTT treatment.^590^ The other design approach involves integrating two materials, typically one providing photosensitizing effects and the other having a high photothermal conversion rate. For instance, Mazin et al. designed and synthesized a multifunctional nanosphere composed of a sodium fluoride core doped with rare earth elements and a PTA bismuth selenide (NaYF4:Yb/Er/Gd,Bi2Se3) shell encapsulating the photosensitizer (Ce6). NaYF4:Yb/Er is excited by NIR through upconversion, Ce6 is responsible for generating ROS, and Bi2Se3 effectively converts NIR into heat.^591^ In addition to PTT, Wang et al. reported the synergistic enhancement of antitumor efficacy through the combination of immunogenic nanoparticles-mediated PDT and magnetic hyperthermia. Moreover, when combined with anti-CTLA-4 antibody, these nanoparticles demonstrated a significant capability to eradicate primary and metastatic tumors, while promoting the development of PDT, hyperthermia, and immunotherapy.^592^

### Phototherapy + chemotherapy

Most in vitro and in vivo experiments suggest that phototherapy directly kills tumor cells through apoptosis and necrosis, as well as through antiangiogenic effects. However, tumor cells that survive phototherapy can lead to the regeneration of tumor cells and tumor vasculature. The concurrent introduction of chemotherapy agent further damages tumor cells, preventing their regeneration. Additionally, chemotherapy itself generates oxidative stress, producing hydroxyl radicals. When combined with phototherapy, these hydroxyl radicals may be sufficient to induce cell cycle arrest and subsequent cytotoxic death of cancer cells.^593^ Phototherapy is a localized treatment method, while chemotherapy is a systemic treatment approach. The systemic action of chemotherapy may help eradicate distant micrometastases, akin to a spatial cooperative effect.^594^ The ROS generated by PDT can inhibit the P-glycoprotein (P-gp) pumps in multidrug-resistant cells, thereby enhancing the efficiency of chemotherapy against multidrug resistance.^595^ Several clinical trials have also indicated that, compared to using PDT or chemotherapy alone, combination phototherapy (CPT) has a stronger antitumor response.^596,597^ In CPT studies, chemotherapy drugs such as Doxorubicin (Dox),^598,599^ Oxaliplatin,^587^ Paclitaxel,^600^ Lenvatinib,^601^ and Cisplatin^602^ are commonly used. Shao et al. first coated mesoporous silica on graphene, which was subsequently modified with hyaluronic acid (pRGO@MS-HA), and then loaded with Doxorubicin (Dox), constructing a multifunctional nanoplatform for targeted chemo-photothermal therapy (pRGO@MS(DOX)-HA). This nanoplatform exhibited pH-dependent and NIR laser-triggered Dox release, enhancing the chemo-photothermal therapeutic effect and demonstrating superior synergistic efficacy.^603^ Zeng et al. used gold nanorods (Au NRs) as seed crystals to construct a porphyrin MOF-coated Au NR (Au NR@MOF), which, after loading with the chemotherapy drug camptothecin (CPT), successfully developed into a multifunctional nanoplatform. This platform was proven to possess a high drug loading capacity, NIR-induced drug release, and photothermal activity to facilitate combined chemothermal and PDT.^604^ Zhu et al. designed and fabricated Tirapazamine (TPZ)-PEG nanoparticles that respond to the acidic tumor microenvironment through the cleavage of acrylamide bonds, enabling controlled release of chemotherapy drugs. After encapsulating the semiconductor polymer (TDPP) into TPZ-PEG, the resulting TDPP@PEG–TPZ NPs exhibited high photostability, significant NIR-II absorption, and exceptionally high photothermal conversion efficiency. Therefore, TDPP@PEG–TPZ NPs enhance chemotherapy by activating TPZ under hypoxic tumor conditions and initiating photothermal therapy via NIR-II activation.^605^

## Conclusion and outlooks

This review elaborates in detail on the primary principles and biological effects of PDT, PTT, and PIT in the context of antitumor activity, highlighting its intricate relationship with various cell death modalities. Additionally, we delve into how PDT and PTT activate and inhibit the immune microenvironment. Subsequently, this review systematically categorizes the obstacles hindering the development and application of phototherapy into four: PTs, O_2_, light, and immune response. The focus of the review is on introducing various strategies proposed and implemented by researchers to address these deficiencies individually. Furthermore, the mechanisms underlying the effectiveness of these strategies are detailed. In the section on PTs, we predominantly highlight various nanomaterials with distinctive photoelectric characteristics that can serve as PS or PTA. Furthermore, the high surface area, tunability, and modifiability of nanomaterials make nanocarrier PT systems a focal point in phototherapy research. In addressing tumor hypoxia, this review summarizes existing approaches into two categories: increasing intra-tumoral oxygen concentration and reducing the oxygen dependence of PDT. This review then delves into the mechanisms and research advancements related to enhancing light tissue penetration through spontaneous luminescence, NIR excitation, non-photo-induced phototherapy (such as microwaves, ultrasound, and X-rays), as well as the implantation of wireless light source devices. Subsequently, we discuss strategies to enhance PIT-induced immune responses and reverse the immunosuppressive TME. These strategies include combining treatments with drugs that can modulate immunosuppressive cell populations, as well as integrating approaches with ICIs and immunoadjuvants. Finally, we explore the advantages of synergetic treatments, such as PDT + PTT and phototherapy + chemotherapy, and the potential reasons for their mutual amplification.

The various nanomaterials and strategies mentioned above have demonstrated promising potential in overcoming different obstacles, but most of them are still at the conceptual validation stage, and there are still many uncertain challenges that need further and more in-depth research on nanomaterials and technologies. (1) Biocompatibility and safety of nanomaterials: Currently, research on the biocompatibility and safety of the numerous emerging nanomaterials and composite nanomaterials is not thorough enough, and there lack of standardized validation for the uptake, distribution, pharmacokinetics, and long-term toxicity in biological systems. In particular, the effects of long-term exposure to inorganic nanomaterials (such as metals) on cell and embryonic development, as well as their safety, need further confirmation. (2) Nanoparticle leakage and retention: the leakage of nanoparticles during circulation and their retention in normal tissues (such as the liver) may exacerbate their damage to normal tissues and organs. Tumor microenvironment-responsive nanocomposites may offer a solution to this issue. Tumor tissues have a microenvironment with low pH, low oxygen, and higher levels of glutathione, hydrogen peroxide, and metabolites. By rationally designing and constructing tumor microenvironment-responsive nano-PTs, the specificity, efficacy, and safety of tumor treatment can be greatly improved. (3) Targeting of nanomaterials: Although in recent years, developed nanocarriers can passively accumulate in tumors via the EPR effect or actively target specific receptors-ligands, the penetration of nanomaterials into large and poorly perfused deep-seated tumors remains inadequate. Additionally, the non-uniform distribution of nanocarriers within tumors makes it difficult to achieve uniform delivery of ROS or heat to the tumor. Thus, nanoplatforms which are capable of effectively accumulating and penetrating tumor tissues are of key importance for enhancing the overall efficacy of phototherapy. Morphology- and size-deformable nanoparticles capable of endosomal escape show great promise, as they can trigger ROS and heat generation through spatiotemporal control by deforming themselves to enhance phototherapy efficacy and reduce side effects.

Additionally, with the advancement of nanomaterials and bioengineering, researchers are increasingly employing a combination of different materials and therapeutic modalities to design solutions that simultaneously address multiple drawbacks, thereby significantly enhancing its antitumor efficacy. Ironically, complex designs imply challenging synthetic processes, low yields, uncertain potential toxicity, and difficulties in clinical translation. Regardless of how intricate and interconnected the design may be, it ultimately must confront a set of highly prominent challenges: how to achieve scalable production, how to address biosafety concerns, and how to facilitate clinical translation. Therefore, we contend that understanding the strengths, weaknesses, and principles of each step in phototherapy allows us to strike a balance when contemplating improvements in phototherapy.

It is noteworthy that, in addition to significant advancements in tumor treatment, phototherapy is also favored for its ability to intervene in multiple pathogenic factors of skin diseases. In recent years, advancements in light source selection, photosensitizers, and treatment indications have been observed due to the widespread application of PDT in dermatology. Daylight-mediated photodynamic therapy (DL-PDT), which uses daylight to activate the photosensitizer, has gained significant attention and approval for treating multiple actinic keratosis occurrences in exposed areas.^606^ DL-PDT has also shown promise in treating actinic cheilitis,^607^ Bowen’s disease,^608^ basal cell carcinoma,^609^ and acne.^610^ Portable LED devices have enabled outpatient PDT treatment, contributing to reduced hospitalization rates.^611^ PDT’s application in skin tumors has been a research focus due to its ability to penetrate tumor tissues and induce cytotoxic effects. It has emerged as a potential neoadjuvant treatment method for nonmelanoma skin cancers, especially in cases of large, multiple, or cosmetically and functionally significant tumors.^612^ Beyond treating skin diseases, PDT can assist in disease diagnosis by aiding in tumor margin determination and lesion detection beyond tumor boundaries, particularly in cases with multiple tumors or unclear boundaries.^613^ However, due to phototherapy’s ongoing research stage for many diseases and the lack of standardized treatment protocols, further strengthening of basic and clinical research is necessary to clarify its efficacy in treating diseases, laying the foundation for the future treatment of more refractory skin diseases.
